# 7th drug hypersensitivity meeting: part one

**DOI:** 10.1186/s13601-016-0121-z

**Published:** 2016-08-25

**Authors:** Daniel F. Carr, Wen-Hung Chung, Rosalind E. Jenkiins, Mas Chaponda, Gospel Nwikue, Elena M. Cornejo Castro, Daniel J. Antoine, Munir Pirmohamed, Natascha Wuillemin, Dolores Dina, Klara K. Eriksson, Daniel Yerly, Rebecca Pavlos, Elizabeth Mckinnin, David Ostrov, Bjoern Peters, Soren Buus, David Koelle, Abha Chopra, Craig Rive, Alec Redwood, Susana Restrepo, Austin Bracey, Jing Yuan, Silvana Gaudieri, Mary Carrington, David Haas, Simon Mallal, Elizabeth Phillips, Douwe De Boer, Paul Menheere, Chris Nieuwhof, Judith Bons, Friederike Jonsson, Luc De Chaisemartin, Vanessa Granger, Caitlin Gillis, Aurelie Gouel, Catherine Neukirch, Fadia Dib, Pascale Roland Nicaise, Dan Longrois, Florence Tubach, Sylvie Martin, Pierre Bruhns, NASA Study Group, Kai-Lung Chen, Shu-Ling Liao, Yi-Shuan Sheen, Yung-Tsu Cho, Che-Wen Yang, Jau-Yu Liau, Chia-Yu Chu, Rita Aguiar, Anabela Lopes, Natália Fernandes, Leonor Viegas, M. A. Pereira-Barbosa, Antonia Bünter, Nisha Gupta, Tatjana Pecaric Petkovic, Nicole Wirth, Werner J. Pichler, Oliver Hausmann, Mehtap Yazicioglu, Pinar G. Ozdemir, Gokce Ciplak, Ozkan Kaya, Peter John Cooke, Inês Mota, Ângela Gaspar, Filipe Benito-Garcia, Marta Chambel, Mário Morais-Almeida, Luis Marques, Eva Alcoceba, Silvia Lara, Leonor Carneiro-Leão, Carmen Botelho, Eunice Dias-Castro, Josefina R. Cernadas, Katherine Nicholls, William Lay, Olivia Smith, Christine Collins, Gary Unglik, Kymble Spriggs, Priscilla Auyeung, Jeremy McComish, Jo A. Douglass, Jonny G. Peter, Paul Potter, Fabrícia Carolino, Eunice Dias De Castro, Ana Sofia Moreira, Carmo Abreu, Eva Gomes, Bárbara Kong Cardoso, Elza Tomaz, Sara Correia, Filipe Inácio, Annabelle Arnold, Natasha Bear, Kristina Rueter, Grace Gong, Michael O’Sullivan, Saravanan Muthusamy, Valerie Noble, Michaela Lucas, Neringa Buterleviciute, Odilija Rudzeviciene, Carmo Abreu, Sara May, Thanai Pongdee, Miguel Park, Linas Griguola, Arturas Vinikovas, Simona Kašinskaite, Violeta Kvedariene, Ayse Aktas, Suheyla Rahman, Huseyin Elbi, Beyhan Cengiz Ozyurt, Ozlem Cavkaytar, Betul Karaatmaca, Pinar Gur Cetinkaya, Saliha Esenboga, Umit M. Sahiner, Bulent E. Sekerel, Ozge Soyer, Celia Zubrinich, Bianca Tong, Mittal Patel, Michelle Giles, Robyn O’Hehir, Robert Puy, Luís Amaral, Semra Demir, Asli Gelincik, Muge Olgac, Raif Caskun, Derya Unal, Bahauddin Colakoglu, Suna Buyukozturk, Olga Vega Matute, Amalia Bernad, Gabriel Gastaminza, Roselle Madamba, Carlos Lacasa, M. J. Goikoetxea, Carmen D’Amelio, Jose Rifón, Nicolas Martínez, Marta Ferrer, Carmelita Ribeiro, Emília Faria, Cristina Frutuoso, Anabela Barros, Rosário Lebre, Alice Pego, Ana Todo Bom, Luis Felipe Ensina, Carolina Aranda, Ines Camelo Nunes, Ana Maria Martins, Dirceu Solé, Sevim Bavbek, Resat Kendirlinan, Pamir Çerçi, Seda Tutluer, Sadan Soyyigit, Zeynep Çelebi Sözener, Ömür Aydin, Reyhan Gümüsburun, Marta Almeida, Kimie Sai, Takuya Imatoh, Ryosuke Nakamura, Chisato Fukazawa, Yasushi Hinomura, Yoshiro Saito, Bernardo Sousa-Pinto, Cláudia Correia, Lídia Gomes, Sara Gil-Mata, Luís Araújo, Luís Delgado, Kimie Sai, Yoshimi Okamoto-Uchida, Koji Kajinami, Kayoko Matsunaga, Michiko Aihara, Chuang-Wei Wang, Shih-Chi Su, Shuen-Iu Hung, Hsin-Chun Ho, Chih-Hsun Yang, Maren Paulmann, Ariane Dunant, Maja Mockenhaupt, Peggy Sekula, Martin Schumacher, Sylvia Kardaun, Luigi Naldi, Teresa Bellón, Daniel Creamer, Cynthia Haddad, Bruno Sassolas, Bénédicte Lebrun-Vignes, Laurence Valeyrie-Allanore, Jean-Claude Roujeau, Maren Paulmann, Carmen Kremmler, Maja Mockenhaupt, Roni P. Dodiuk-Gad, Cristina Olteanu, Anthony Feinstein, Rena Hashimoto, Raed Alhusayen, Sonia Whyte-Croasdaile, Yaron Finkelstein, Marjorie Burnett, Shachar Sade, Robert Cartotto, Marc Jeschke, Neil H. Shear, Naoko Takamura, Yumiko Yamane, Setsuko Matsukura, Kazuko Nakamura, Yuko Watanabe, Yukie Yamaguchi, Takeshi Kambara, Zenro Ikezawa, Michiko Aihara, Rena Hashimoto, Hall Chew, Marjorie Burnett, Marc Jeschke, Brittany Knezevic, Una Nic Ionmhain, Allison Barraclough, Matthew Anstey, Toru Usui, Xiaoli Meng, John Farrell, Paul Whitaker, John Watson, Neil French, Kevin Park, Dean Naisbitt, Ana Castro Neves, Susana Cadinha, Ana Moreira, J. P. Moreira Da Silva, Daniela Ledic Drvar, Sandra Jerkovic Gulin, Suzana Ljubojevic Hadzavdic, Romana Ceovic, Ana Montoro De Francisco, Talía De Vicente Jiménez, Amelia García Luque, Natalia Rosado David, José Mª Mateos Galván, Razvigor Darlenski, Dario Gulin, Jozica Sikic, Jasna Cerkez Habek, Edvard Galic, Philip Specht, Doris Staab, Beate Mayer, Jobst Roehmel, Caius Solovan, Anca Chiriac, Paola Djurinec, Kresimir Kostovic, Mirna Bradamante, Jose Pedro Almeida, Joana Caiado, Elisa Pedro, Pedro Canas Da Silva, Manuel Pereira Barbosa, Gador Bogas, Natalia Blanca-López, Diana Pérez-Alzate, Inmaculada Doña, José Augusto Agúndez, Elena García-Martín, José Antonio Cornejo-García, Cristobalina Mayorga, María José Torres, Maria Gabriela Canto, Miguel Blanca, Sengül Aksakal, Aytül Zerrin Sin, Zeynep Peker Koç, Fatma Düsünür Günsen, Ömür Ardeniz, Emine Nihal Mete Gökmen, Okan Gülbahar, Ali Kokuludag, Natalia Pérez-Sánchez, María Salas, Maria Salas, Francisca Gomez, Esther Barrionuevo, Inmaculada Andreu, Miguel Ángel Miranda, Gabija Didžiokaite, Olesia Gaidej, Simona Kašinskaite, Maria Isabel Garcimartin, Maria Luisa Somoza, Gador Bojas, Jose Antonio Cornejo-Garcia, Francisco Javier Ruano Perez, Miguel Angel Miranda, Elina Jerschow, Teresa Pelletier, Zhen Ren, Golda Hudes, Marek Sanak, Esperanza Morales, Victor Schuster, Simon D Spivack, David Rosenstreich, Renato Erzen, Mira Silar, Nissera Bajrovic, Matija Rijavec, Mihaela Zidarn, Peter Korosec, Eunice Castro, Mona Al-Ahmad, Tito Rodriguez, João Pedro Azevedo, Beatriz Tavares, Frederico Regateiro, Ana Todo-Bom, Pablo Andrés Miranda, Bautista De La Cruz Hoyos, Waleed Abuzeid, Nadeem Akbar, Marc Gibber, Marvin Fried, Weiguo Han, Taha Keskin, Robert Tamayev, Simon D. Spivack, David Rosenstreich, Elina Jerschow, Elisa Boni, Marina Russello, Marina Mauro, Marta Ferreira Neto, Lise Brosseron, Daniela Malheiro, Patrícia Barreira, Dustin Sprigg, Michelle Trevenen, Jason Seet, Jason Trubiano, William Smith, Yogesh Jeelall, Sandra Vale, Richard Loh, Andrew Mclean-Tooke, Sabine Müller, Ursula Amstutz, Lukas Jörg, Nikhil Yawalkar, Stephan Krähenbühl, Ana Leblanc, Laura Ribeiro, Arantza Vega, Raquel Gutierrez Rivas, Ana Alonso, Juan Maria Beitia, Belén Mateo, Remedios Cárdenas, Juan Jesus Garcia-Dominguez, Rebecca Pavlos, Kaija Strautins, Ian James, Simon Mallal, Alec Redwood, Rita Aguiar, Anabela Lopes, Ana Neves, Maria Do Céu Machado, Ceyda Tunakan Dalgiç, Emine Nihal Mete Gökmen, Gökten Bulut, Fatma Ömür Ardeniz, Okan Gülbahar, Aytül Zerrin Sin, Shao-Hsuan Hsu, Che-Wen Yang, Young-Min Ye, Gyu-Young Hur, Hae-Sim Park, Seung-Hyun Kim, Syed Ali, Peter N. Hollingsworth, Andrew P. C. Mclean-Tooke, Zohra Chadly, Nadia Ben Fredj, Karim Aouam, Haifa Ben Romdhane, Naceur A. Boughattas, Amel Chaabane, Marina Lluncor Salazar, Beatriz Pola, Ana Fiandor, Elena Ramírez, Javier Domínguez Ortega, Santiago Quirce, Rosario Cabañas, Krasimira Baynova, Marina Labella, Manuel Prados, Agne Ramonaite, Ieva Bajoriuniene, Brigita Sitkauskiene, Raimundas Sakalauskas, Jae-Woo Kwon, Shinyoung Park, Diana Silva, Leonor Carneiro Leão, Eunice Castro, Maria Garcimartin, Maria Vazquez De La Torre, Francisco Javier Ruano Pérez, Elisa Haroun, Gabriela Canto Diez, Katinka Ónodi-Nagy, Ágnes Kinyó, Lajos Kemény, Zsuzsanna Bata-Csörgo, Joana Sofia Pita, Rosa Anita Fernandes, Ana Moura, Nuno Sousa, Carlos Loureiro, Wolfgang Pfützner, Nadine Marrouche, Clive Grattan, Yu-En Chen, Chun-Bing Chen, Yu-Ping Hsiao, Maria Isabel Garcimartin, Francisco Javier Ruano

**Affiliations:** 1 University of Liverpool, Liverpool, UK; 2 Chang Gung Memorial Hospital, Taipei, Taiwan; 3 University Hospital Bern, Bern, Switzerland; 4 Murdoch University, Perth, Australia; 5 University of Florida, Gainesville, FL USA; 6 La Jolla Institute for Allergy and Immunology, La Jolla, CA USA; 7 University of Copenhagen, Copenhagen, Denmark; 8 University of Washington, Seattle, WA USA; 9 Boehringer Ingelheim Inc, Ridgefield, CT USA; 10 University of Western Australia, Perth, Australia; 11 Ragon Institute, Cambridge, MA USA; 12 Vanderbilt University, Nashville, TN USA; 13 Central Diagnostic Laboratory, MUMC+, Maastricht, The Netherlands; 14 Internal Medicine, MUMC+, Maastricht, The Netherlands; 15 Institut Pasteur, Paris, France; 16 Hopital Bichat, Paris, France; 17 Institut Pasteur & Hopital Bichat, Paris, France; 18 Department of Dermatology, National Taiwan University Hospital and National Taiwan University College of Medicine, Taipei, Taiwan; 19 Department of Pathology, National Taiwan University Hospital and National Taiwan University College of Medicine, Taipei, Taiwan; 20 Immunoallergology Department, Hospital de Santa Maria-Centro Hospitalar Lisboa Norte, Lisbon, Portugal; 21 ADR-AC GmbH, Bern, Switzerland; 22 Teleflex Incorporated, Bern, Switzerland; 23 Dep. of Rheumatology, Immunology and Allergology, University Hospital and University of Bern, Bern, Switzerland; 24 Department of Pediatric Allergy, Trakya University, Edirne, Turkey; 25 Department of Pediatrics, Trakya University, Edirne, Turkey; 26 Trakya University, Edirne, Turkey; 27 Auckland City Hospital, Auckland, New Zealand; 28 Immunoallergy Department, CUF Descobertas Hospital, Lisbon, Portugal; 29 Hospitals Universitaris Santa Maria - Arnau de Vilanova, Lleida, Spain; 30 Serviço de Imunoalergologia, Centro Hospitalar de São João, E.P.E., Porto, Portugal; 31 Department of Immunology and Allergy, The Royal Melbourne Hospital, Parkville, Australia; 32 Department of Medicine, University of Melbourne, Parkville, Australia; 33 University of Cape Town, Cape Town, South Africa; 34 Centro Hospitalar Vila Nova Gaia e Espinho, Vila Nova Gaia, Portugal; 35 Centro Hospitalar do Porto, Porto, Portugal; 36 Hospital de S.Bernardo - Centro Hospitalar de Setúbal, Setúbal, Portugal; 37 Department of Immunology, Princess Margaret Hospital, Perth, Australia; 38 Telethon Kids Institute, Department of Clinical Research and Education, Princess Margaret Hospital, Perth, Australia; 39 Department of Immunology, Department of Clinical Research and Education, Princess Margaret Hospital, Telethon Kids Institute, Perth, Australia; 40 Department of Immunology, PathWest Laboratory Medicine WA, Princess Margaret Hospital, Perth, Australia; 41 Department of Immunology, Princess Margaret Hospital, PathWest Laboratory Medicine WA, Fiona Stanley Hospital, School of Pathology and Laboratory Medicine, University of Western Australia, Perth, Australia; 42 Department of Immunology, Princess Margaret Hospital, Sir Charles Gairdner Hospital, PathWest Laboratory Medicine WA, School of Medicine and Pharmacology, School of Pathology and Laboratory Medicine, University of Western Australia, Institute of Immunology and Infectious Diseases, Murdoch University, Perth, Australia; 43 Faculty of Medicine Centre of Children Pulmonology and Allergology, Vilnius University, Vilnius, Lithuania; 44 University of Nebraska Medical Center, Omaha, NE USA; 45 Mayo Clinic, Jacksonville, FL USA; 46 Mayo Clinic, Rochester, NY USA; 47 Faculty of Medicine, Vilnius University, Vilnius, Lithuania; 48 Center of Pulmonology and Allergology, Clinic of Infectious, Chest diseases, Dermatology and Allergology, Vilnius University Hospital Santariskiu Klinikos, Vilnius, Lithuania; 49 Celal Bayar University, Manisa, Turkey; 50 Department of Pediatric Allergy, Hacettepe University School of Medicine, Ankara, Turkey; 51 Alfred Health, Melbourne, Australia; 52 Istanbul Faculty of Medicine, Istanbul University, Istanbul, Turkey; 53 Department of Allergy and Immunology, Clínica Universidad de Navarra, Pamplona, Spain; 54 Department of Pharmacy, Clínica Universidad de Navarra, Pamplona, Spain; 55 Department of Hematology, Clínica Universidad de Navarra, Pamplona, Spain; 56 Allergy and Clinical Immunology Department, Coimbra University Hospital Center, Coimbra, Portugal; 57 Oncology Department, Coimbra University Hospital Center, Coimbra, Portugal; 58 Federal University of São Paulo, São Paulo, Brazil; 59 Ankara University, Ankara, Turkey; 60 Serviço de Pediatria, Instituto Português de Oncologia do Porto Francisco Gentil, Porto, Portugal; 61 National Institute of Health Sciences, Tokyo, Japan; 62 Japan Pharmaceutical Information Center, Tokyo, Japan; 63 Immunology Laboratory - Basic and Clinical Immunology, Faculty of Medicine, Porto, Portugal; 64 Kanazawa Medical University, Ishikawa, Japan; 65 Fujita Health University, Aichi, Japan; 66 Yokohama City University, Kanagawa, Japan; 67 Department of Dermatology, Drug Hypersensitivity Clinical and Research Center, Chang Gung Memorial Hospital, Linkou, Keelung, Taiwan; 68 Department and Institute of Pharmacology, School of Medicine, Infection and Immunity Research Center, National Yang-Ming University, Linkou, Taiwan; 69 Dokumentationszentrum schwerer Hautreaktionen, University Medical Center, Freiburg, Germany; 70 Department of Biostatistics and Epidemiology Unit, Institut Gustave-Roussy, Villejuif, France; 71 Institute of Medical Biometry and Medical Informatics, University Medical Center, Freiburg, Germany; 72 Reference Center for Cutaneous Adverse Reactions, University Medical Center, Groningen, The Netherlands; 73 Department of Dermatology, Papa Giovanni XXIII Hospital, Bergamo, Italy; 74 Institute for Health Research, University Hospital La Paz–IdiPAZ, Madrid, Spain; 75 Department of Dermatology, King’s College Hospital, London, UK; 76 Reference Center for Toxic and Autoimmune Blistering Diseases, Hopital Henri Modor, University Paris-Est, Créteil, France; 77 Department of Internal Medicine and Respiratory Diseases, Hôpital Cavale Blanche, Brest, France; 78 Department of Pharmacology, Hôpital Pitié-salpétrière, Paris, France; 79 Department of Dermatology, Ha’emek Medical Center, Afula, Israel; 80 Faculty of Medicine, University of Toronto, Toronto, Canada; 81 Department of Psychiatry, Sunnybrook Health Sciences Centre, Toronto, Canada; 82 Division of Dermatology, Department of Medicine, Sunnybrook Health Sciences Centre, Toronto, Canada; 83 SJS and TENS Group Canada-CAST International, Toronto, Canada; 84 Paediatric Emergency Medicine, Clinical Pharmacology and Toxicology, The Hospital for Sick Children, Toronto, Canada; 85 Ross Tilley Burn Centre, Sunnybrook Health Sciences Centre, Toronto, Canada; 86 Department of Pathology, Sunnybrook Health Sciences Centre, Toronto, Canada; 87 Department of Environmental Immuno-Dermatology, Yokohama City University, Yokohama, Japan; 88 Department of Dermatology, Yokohama City University Medical Center, Yokohama, Japan; 89 Department of Ophthalmology and Vision Sciences, Sunnybrook Health Sciences Centre, Toronto, Canada; 90 Sir Charles Gairdner Hospital, Perth, Australia; 91 Pathwest Laboratory Medicine, Queen Elizabeth II Medical Centre, Perth, Australia; 92 MRC Centre for Drug Safety Science, Dept Molecular & Clinical Pharmacology, University of Liverpool, Liverpool, UK; 93 Centro Hospitalar de Vila Nova de Gaia/Espinho, Vila Nova De Gaia, Portugal; 94 Department of Dermatology and Venereology, University Hospital Center Zagreb and School of Medicine, Zagreb, Croatia; 95 Department of Dermatology and Venereology, General Hospital Sibenik, Sibenik, Croatia; 96 Hospital Central de la Defesa, IMIDEF, Madrid, Spain; 97 Tokuda Hospital Sofia, Sofia, Bulgaria; 98 University Hospital Sveti Duh, Zagreb, Croatia; 99 General Hospital Sibenik, Sibenik, Croatia; 100 Division of Cystic Fibrosis, Pediatric Pneumology and Immunology, Charité-Universitätsmedizin Berlin, Berlin, Germany; 101 Institute for Transfusion Medicine, Charité - Universitätsmedizin Berlin, Berlin, Germany; 102 Department of Dermatology, University of Medicine and Pharmacy, Tamisoara, Romania; 103 Dermatology Department, Nicolina Medical Centre, Apollonia University, “P.Poni” Research Institute of Macromolecular Chemistry, Iasi, Romania; 104 Department of Dermatology and Venereology, University Hospital Center Zagreb and School of Medicine Zagreb, Zagreb, Croatia; 105 Immunoallergology Department, Centro Hospitalar Lisboa Norte/Hospital Santa Maria, Lisbon, Portugal; 106 Cardiology Department, Centro Hospitalar Lisboa Norte/Hospital Santa Maria, Lisbon, Portugal; 107 Allergy Unit, IBIMA, Regional University Hospital of Malaga, UMA, Malaga, Spain; 108 Allergy Service, Infanta Leonor University Hospital, Madrid, Spain; 109 Department of Pharmacology, University of Extremadura, Caceres, Spain; 110 Research Laboratory and Allergy Unit, IBIMA, Regional University Hospital of Malaga, UMA, Malaga, Spain; 111 Medical Faculty Department of Clinical Immunology, Karadeniz Technical University, Trabzon, Turkey; 112 Medical Faculty Department of Allergy and Clinical Immunology, Ege University, Izmir, Turkey; 113 Allergy Unit, Malaga Regional University Hospital-IBIMA, Malaga, Spain; 114 Allergy Service, Infanta Leonor Hospital, Madrid, Spain; 115 Regional Hospital of Málaga-IBIMA, Málaga, Spain; 116 Infanta Leonor Hospital, Madrid, Spain; 117 Regional Hospital of Málaga, Málaga, Spain; 118 Dpto. Química /Instituto de Tecnología Química –UPV-CSIC, Valencia, Spain; 119 Centre of Internal Medicine, Vilnius University Hospital Santariskiu Klinikos, Vilnius, Lithuania; 120 Allergy Unit, Infanta Leonor University Hospital, Madrid, Spain; 121 Allergy Unit, Regional University Hospital of Malaga, Malaga, Spain; 122 Research Laboratory and Allergy Unit, IBIMA, Regional University Hospital of Malaga, Malaga, Spain; 123 Universidad Politécnica de Valencia, Valencia, Spain; 124 Albert Einstein College of Medicine/Montefiore Medical Center, Bronx, NY USA; 125 Albert Einstein College of Medicine, Bronx, NY USA; 126 Jacobi Medical Center, New York, NY USA; 127 Albert Einstein College of Medicine, Montefiore Medical Center, Bronx, NY USA; 128 Jagiellonian University Medical College, Bronx, Poland; 129 Ferkauf Graduate School of Psychology, Yeshiva University, New York, NY USA; 130 Albert Einstein College of Medicine/Montefiore Medical Center, Krakow, WI USA; 131 University Hospital for Respiratory Diseases and Allergy Golnik, Golnik, Slovenia; 132 Microbiology Department, Faculty of Medicine, Kuwait University, Kuwait, Kuwait; 133 Al Rashed Allergy Center, Kuwait, Kuwait; 134 Centro Hospitalar e Universitário de Coimbra, Coimbra, Portugal; 135 Universidad Nacional de Colombia, Cartagena, Colombia; 136 Centro de Especialistas Santo Domingo - Alergologia, Cartagena, Colombia; 137 Regional Adult Cystic Fibrosis Unit, St James’s Hospital, Leeds, UK; 138 Hospital Sant’Anna, Como, Italy; 139 CHLN-HSM, Lisbon, Portugal; 140 Austin Health, Melbourne, Australia; 141 Royal Adelaide Hospital, Adelaide, Australia; 142 Australian Society of Clinical Immunology and Allergy, Balgowlah, Australia; 143 Princess Margaret Hospital, Perth, Australia; 144 Department of Dermatology, Drug Hypersensitivity Clinical and Research Center, Chang Gung Memorial Hospitals, Taipei, Taiwan; 145 Division of Dermatology, Department of Medicine, Sunnybrook Health Sciences Centre, Linkou, Canada; 146 Department of Clinical Pharmacology and Regional Pharmacovigilance Center, Inselspital, University Hospital Bern, Bern, Switzerland; 147 University Institute of Clinical Chemistry, Inselspital, University Hospital Bern and University of Bern, Bern, Switzerland; 148 Department of Rheumatology, Clinical Immunology and Allergology, Inselspital, University Hospital Bern, Bern, Switzerland; 149 Department of Dermatology, Inselspital, University Hospital Bern, Bern, Switzerland; 150 Allergy and Clinical Immunology Department, Centro Hospitalar S. João EPE, Porto, Portugal; 151 Biochemistry Department. Medical Education and Simulation Department. Faculty of Medicine, University of Porto, Porto, Portugal; 152 Hospital Universitario de Guadalajara, Guadalajara, Spain; 153 Universidad de Alcalá, Alcalá De Henares, Spain; 154 Pediatrics Department, Hospital de Santa Maria-Centro Hospitalar Lisboa Norte, Lisbon, Portugal; 155 Department of Allergy and Clinical Immunology, Ege University Medical Faculty, Izmir, Turkey; 156 Department of Allergy & Clinical Immunology, Ajou University School of Medicine, Suwon, South Korea; 157 Department of Immunology, Perth, Australia; 158 Department of Immunology, Pathwest, QE2 Medical Centre, Perth, Australia; 159 SMP, PALM, UWA, IIID, Murdoch University, Perth, Australia; 160 Faculty of Medicine/University hospital, University of Monastir, Monastir, Tunisia; 161 Allergy Department, La Paz Hospital Institute for Health Research (IdiPAZ), Madrid, Spain; 162 Allergy Department, La Paz Hospital Institute for Health Research (IdiPAZ), Consorcio Piel en RED, Madrid, Spain; 163 Immunology Department, La Paz Hospital Institute for Health Research (IdiPAZ), Consorcio Piel en RED, Madrid, Spain; 164 Department of Clinical Pharmacology, Hospital La Paz Health Research Institute (IdiPAZ), School of Medicine, Consorcio Piel en RED, Universidad Autónoma de Madrid, Madrid, Spain; 165 HUVR Seville, Seville, Spain; 166 Department of Pulmonology and Immunology, Lithuanian University of Health Sciences, Kaunas, Lithuania; 167 Department of Allergy and Clinical Immunology, Kangwon National University School of Medicine, Chuncheon, Korea; 168 The Research Department, Kangwon Regional Cancer Center, Kangwon National University Hospital, Chuncheon, Korea; 169 Allergy and Clinical Immunology Department, São João Medical Center, Porto, Portugal; 170 Laboratory of Basic & Clinical Immunology, Faculty of Medicine, Porto University, Porto, Portugal; 171 Hospital Universitario Infanta Leonor, Madrid, Spain; 172 Department of Dermatology and Allergology, Szeged, Hungary; 173 Department of Dermatology, Venereology and Oncodermatology, Pécs, Hungary; 174 Centro Hospitalar de Leiria, Leiria, Portugal; 175 Department of Dermatology and Allergology, Philipps University Marburg, Marburg, Germany; 176 Norfolk and Norwich University Hospital, Norwich, UK; 177 College of Medicine, Chung Shan Medical University Hospital and Chung Shan Medical University, Taichung, Taiwan; 178 Department of Dermatology, Drug hypersensitivity clinical and research center, Chang Gung Memorial Hospitals, Linkou, Taipei, Keelung, Taiwan; 179 Department of Dermatology, Chung Shan Medical University Hospital and Chung Shan Medical University College of Medicine, Taichung, Taiwan; 180 Hospital Infanta Leonor, Madrid, Spain

## Abstract

Oral Abstracts

O1 Functionally distinct HMGB1 isoforms correlate with physiological processes in drug-induced SJS/TEN

Daniel F. Carr, Wen-Hung Chung, Rosalind E. Jenkiins, Mas Chaponda, Gospel Nwikue, Elena M. Cornejo Castro, Daniel J. Antoine, Munir Pirmohamed

O2 Hypersensitivity reactions to beta-lactams, does the t cell recognition pattern influence the clinical picture?

Natascha Wuillemin, Dolores Dina, Klara K. Eriksson, Daniel Yerly

O3 Specific binding characteristics of HLA alleles associated with nevirapine hypersensitivity

Rebecca Pavlos, Elizabeth Mckinnin, David Ostrov, Bjoern Peters, Soren Buus, David Koelle, Abha Chopra, Craig Rive, Alec Redwood, Susana Restrepo, Austin Bracey, Jing Yuan, Silvana Gaudieri, Mary Carrington, David Haas, Simon Mallal, Elizabeth Phillips

O4 Do we need to measure total ige for the interpretation of analytical results of ImmunoCAP dnd 3gAllergy specific IgE?

Douwe De Boer, Paul Menheere, Chris Nieuwhof, Judith Bons

O5 Neutrophil activation in systemic anaphylaxis: results from the multicentric NASA study

Friederike Jonsson, Luc De Chaisemartin, Vanessa Granger, Caitlin Gillis, Aurelie Gouel, Catherine Neukirch, Fadia Dib, Pascale Roland Nicaise, Dan Longrois, Florence Tubach, Sylvie Martin, Pierre Bruhns, NASA Study Group

O6 Purpuric drug eruptions due to epidermal growth factor receptor tyrosine kinase inhibitors (EGFR-TKIs) for non-small-cell lung cancer (NSCLC): a clinic-pathological study of 32 cases

Kai-Lung Chen, Shu-Ling Liao, Yi-Shuan Sheen, Yung-Tsu Cho, Che-Wen Yang, Jau-Yu Liau, Chia-Yu Chu

Poster presentations: Poster Walk 1—Anaphylaxis (P01–P09)

P1 Anaphylactic reactions during anaesthesia and the perioperative period

Rita Aguiar, Anabela Lopes, Natália Fernandes, Leonor Viegas, M. A. Pereira-Barbosa

P2 Anaphylaxis to chlorhexidine: is there a cross-reactivity to alexidine?

Antonia Bünter, Nisha Gupta, Tatjana Pecaric Petkovic, Nicole Wirth, Werner J. Pichler, Oliver Hausmann

P3 Cefotaxime-induced severe anaphylaxis in a neonate

Mehtap Yazicioglu, Pinar G. Ozdemir, Gokce Ciplak, Ozkan Kaya

P4 Clinical features and diagnosis of anaphylaxis resulting from exposure to chlorhexidine

Peter John Cooke

P5 Drug-induced anaphylaxis: five-year single-center survey

Inês Mota, Ângela Gaspar, Filipe Benito-Garcia, Marta Chambel, Mário Morais-Almeida

P6 Intraoperative severe anaphylactic reaction due to patent blue v dye

Luis Marques, Eva Alcoceba, Silvia Lara

P7 Kounis syndrome in the setting of anaphylaxis to diclofenac

Leonor Carneiro-Leão, Carmen Botelho, Eunice Dias-Castro, Josefina Cernadas

P8 Perioperative anaphylaxis audit: Royal Melbourne Hospital

Katherine Nicholls, William Lay, Olivia Smith, Christine Collins, Gary Unglik, Kymble Spriggs, Priscilla Auyeung, Jeremy McComish, Jo A. Douglass

P9 Recurrent peri-operative anaphylaxis: a perfect storm

Jonny G. Peter, Paul Potter

Poster Walk 2: DH regions and patient groups (P10–P19)

P10 A rare presentation of amoxicillin allergy in a young child

Fabrícia Carolino, Eunice Dias De Castro, Josefina R. Cernadas

P11 Adverse drug reactions in children: antibiotics or virus?

Ana Sofia Moreira, Carmo Abreu, Eva Gomes

P12 Allergic reactions in invasive medical procedures

Bárbara Kong Cardoso, Elza Tomaz, Sara Correia, Filipe Inácio

P13 Antibiotic allergy in children: room for improvement

Annabelle Arnold, Natasha Bear, Kristina Rueter, Grace Gong, Michael O’Sullivan, Saravanan Muthusamy, Valerie Noble, Michaela Lucas

P14 Drug hypersensitivity reactions in children and results of diagnostic evaluation

Neringa Buterleviciute, Odilija Rudzeviciene

P15 Nonimmediate cutaneous drug reactions in children: are skin tests required?

Ana Sofia Moreira, Carmo Abreu, Eva Gomes

P16 Pediatric patients with a history of penicillin allergy and a positive penicillin skin test may not be at an increased risk for multiple drug allergies

Sara May, Thanai Pongdee, Miguel Park

P17 Proved hypersensitivity to drugs according data of Vilnius University Hospital Santariskiu Klinikos

Linas Griguola, Arturas Vinikovas, Simona Kašinskaite, Violeta Kvedariene

P18 Self-reported prevalence of drug hypersensitivity reactions among students in Celal Bayar University, Turkey

Ayse Aktas, Suheyla Rahman, Huseyin Elbi, Beyhan Cengiz Ozyurt

P19 Severe drug hypersensitivity reactions in pediatric age

Ozlem Cavkaytar, Betul Karaatmaca, Pinar Gur Cetinkaya, Saliha Esenboga, Umit M. Sahiner, Bulent E. Sekerel, Ozge Soyer

Poster Walk 3: Desensitisation (P20–P28)

P20 A protocol for desensitisation to valaciclovir

Celia Zubrinich, Bianca Tong, Mittal Patel, Michelle Giles, Robyn O’Hehir, Robert Puy

P21 A rare case of desensitization to modafinil

Josefina Cernadas, Luís Amaral, Fabrícia Carolino

P22 A sixteen-day desensitization protocol in delayed type hypersensitivity reactions to oral drugs

Semra Demir, Asli Gelincik, Muge Olgac, Raif Caskun, Derya Unal, Bahauddin Colakoglu, Suna Buyukozturk

P23 Desensitization to intravenous etoposide using a 12 and a 13-step protocol. Two cases report

Olga Vega Matute, Amalia Bernad, Gabriel Gastaminza, Roselle Madamba, Carlos Lacasa, M. J. Goikoetxea, Carmen D’Amelio, Jose Rifón, Nicolas Martínez, Marta Ferrer

P24 Drug desensitisation in oncology: the experience of an immunoallergology department for 5 years

Carmelita Ribeiro, Emília Faria, Cristina Frutuoso, Anabela Barros, Rosário Lebre, Alice Pego, Ana Todo Bom

P25 Filgrastim anaphylaxis: a successful desensitization protocol

Luis Amaral, Josefina Cernadas

P26 Galsulfase hypersensitivity and desensitization of a mucopolysaccharidosis VI patient

Luis Felipe Ensina, Carolina Aranda, Ines Camelo Nunes, Ana Maria Martins, Dirceu Solé

P27 Rapid drug desensitization with biologicals: one-center experience with four biologicals

Sevim Bavbek, Resat Kendirlinan, Pamir Çerçi, Seda Tutluer, Sadan Soyyigit, Zeynep Çelebi Sözener, Ömür Aydin, Reyhan Gümüsburun

P28 Successful desensitization to a high dose of methotrexate in a delayed type hypersensitivity reaction

Josefina Cernadas, Leonor Carneiro-Leão, Fabrícia Carolino, Marta Almeida

Poster Walk 4: SJS (P29–P38)

P29 Assessment of impact of infection on drug-induced severe cutaneous adverse reactions and rhabdomyolysis using the Japanese adverse drug event report database

Kimie Sai, Takuya Imatoh, Ryosuke Nakamura, Chisato Fukazawa, Yasushi Hinomura, Yoshiro Saito

P30 Characterization of erythema multiforme and severe cutaneous adverse reactions hospitalizations

Bernardo Sousa-Pinto, Cláudia Correia, Lídia Gomes, Sara Gil-Mata, Luís Araújo, Luís Delgado

P31 Effects of infection on incidence/severity of SJS/TEN and myopathy in Japanese cases analyzed by voluntary case reports

Ryosuke Nakamura, Kimie Sai, Takuya Imatoh, Yoshimi Okamoto-Uchida, Koji Kajinami, Kayoko Matsunaga, Michiko Aihara, Yoshiro Saito

P32 Efficacy of tumor necrosis factor—a antagonists in Stevens–Johnson syndrome and toxic epidermal necrolysis: a randomized controlled trial and immunosuppressive effects evaluation

Chuang-Wei Wang, Shih-Chi Su, Shuen-Iu Hung, Hsin-Chun Ho, Chih-Hsun Yang, Wen-Hung Chung

P33 Evolution of drug causality in Stevens–Johnson syndrome and toxic epidermal necrolysis in Europe: analysis of 10 years RegiSCAR-Study

Maren Paulmann, Ariane Dunant, Maja Mockenhaupt, Peggy Sekula, Martin Schumacher, Sylvia Kardaun, Luigi Naldi, Teresa Bellón, Daniel Creamer, Cynthia Haddad, Bruno Sassolas, Bénédicte Lebrun-Vignes, Laurence Valeyrie-Allanore, Jean-Claude Roujeau

P34 Long-term sequelae in patients with Stevens–Johnson syndrome and toxic epidermal necrolysis: a 5-year analysis

Maren Paulmann, Carmen Kremmler, Peggy Sekula, Laurence Valeyrie-Allanore, Luigi Naldi, Sylvia Kardaun, Maja Mockenhaupt

P35 Major emotional complications and decreased health related quality of life among survivors of Stevens–Johnson syndrome and toxic epidermal necrolysis

Roni P. Dodiuk-Gad, Cristina Olteanu, Anthony Feinstein, Rena Hashimoto, Raed Alhusayen, Sonia Whyte-Croasdaile, Yaron Finkelstein, Marjorie Burnett, Shachar Sade, Robert Cartotto, Marc Jeschke, Neil H. Shear

P36 Retrospective analysis of Stevens–Johnson syndrome and toxic epidermal necrolysis in Japanese patients: treatment and outcome

Naoko Takamura, Yumiko Yamane, Setsuko Matsukura, Kazuko Nakamura, Yuko Watanabe, Yukie Yamaguchi, Takeshi Kambara, Zenro Ikezawa, Michiko Aihara

P37 Severe physical complications among survivors of Stevens–Johnson syndrome and toxic epidermal necrolysis

Roni P. Dodiuk-Gad, Cristina Olteanu, Rena Hashimoto, Hall Chew, Raed Alhusayen, Sonia Whyte-Croasdaile, Yaron Finkelstein, Marjorie Burnett, Shachar Sade, Robert Cartotto, Marc Jeschke, Neil H. Shear

P38 Stevens–Johnson syndrome/toxic epidermal necrolysis combined with haemophagocytic lymphohistiocytosis: a case report

Brittany Knezevic, Una Nic Ionmhain, Allison Barraclough, Michaela Lucas, Matthew Anstey

Poster Walk 5: Other organs/unexpected immune reactions (P39–P47)

P39 A case report of patient with anti-tuberculosis drug-related severe liver failure

Toru Usui, Xiaoli Meng, John Farrell, Paul Whitaker, John Watson, Neil French, Kevin Park, Dean Naisbitt

P40 Acute interstitial nephritis induced by ibuprofen

Ana Castro Neves, Susana Cadinha, Ana Moreira, J. P. Moreira Da Silva

P41 Cetuximab induced acneiform rash—two case reports

Daniela Ledic Drvar, Sandra Jerkovic Gulin, Suzana Ljubojevic Hadzavdic, Romana Ceovic

P42 Enteropathy associated with losartan

Ana Montoro De Francisco, Talía De Vicente Jiménez, Amelia García Luque, Natalia Rosado David, José Mª Mateos Galván

P43 Granuloma annulare after therapy with canakinumab

Razvigor Darlenski

P44 Hypersensitivity eosinophilic myocarditis or acute coronary syndrome? Case report

Dario Gulin, Jozica Sikic, Jasna Cerkez Habek, Sandra Jerkovic Gulin, Edvard Galic

P45 Piperacillin-induced immune haemolytic anaemia: a severe and frequent complication of antibiotic treatment in patients with cystic fibrosis

Philip Specht, Doris Staab, Beate Mayer, Jobst Roehmel

P46 Progesterone triggered pemphigus foliaceus: case report

Sandra Jerkovic Gulin, Caius Solovan, Anca Chiriac

P47 Ramipril: triggered generalized pustular psoriasis

Paola Djurinec, Kresimir Kostovic, Mirna Bradamante, Sandra Jerkovic Gulin, Romana Ceovic

Poster Walk 6: NSAIDs (P48–P56)

P48 Aspirin desensitization in cardiovascular disease—Portuguese experience

Jose Pedro Almeida, Joana Caiado, Elisa Pedro, Pedro Canas Da Silva, Manuel Pereira Barbosa

P49 Asthma and/or rhinitis to NSAIDs with good tolerance to ASA

Gador Bogas, Natalia Blanca-López, Diana Pérez-Alzate, Inmaculada Doña, José Augusto Agúndez, Elena García-Martín, José Antonio Cornejo-García, Cristobalina Mayorga, María José Torres, Gabriela Canto, Miguel Blanca

P50 Clinical characteristics of 196 patients with non-steroidal anti-inflammatory drug (NSAIDs) hypersensitivity

Sengül Aksakal, Aytül Zerrin Sin, Zeynep Peker Koç, Fatma Düsünür Günsen, Ömür Ardeniz, Emine Nihal Mete Gökmen, Okan Gülbahar, Ali Kokuludag

P51 Development of immediate hypersensitivity to several NSAIDs maintaining good tolerance to ASA

Natalia Pérez-Sánchez, Natalia Blanca-López, Diana Pérez-Alzate, Gador Bogas, Inmaculada Doña, María Salas, María José Torres, Miguel Blanca, Gabriela Canto

P52 Diagnosis of hypersensitivity reactions to paracetamol in a large series of cases

Inmaculada Doña, Maria Salas, Francisca Gomez, Natalia Blanca-Lopez, Diana Perez-Alzate, Gador Bogas, Esther Barrionuevo, Maria Jose Torres, Inmaculada Andreu, Miguel Ángel Miranda, Gabriela Canto, Miguel Blanca

P53 Hypersensitivity to paracetamol according to the new classification of hypersensitivity to NSAIDs

Gabija Didžiokaite, Olesia Gaidej, Simona Kašinskaite, Violeta Kvedariene

P54 Ibuprofen and other aryl propionic derivates can induce immediate selective hypersensitivity responses

Diana Perez-Alzate, Natalia Blanca-López, Maria Isabel Garcimartin, Inmaculada Doña, Maria Luisa Somoza, Cristobalina Mayorga, Maria Jose Torres, Gador Bojas, Jose Antonio Cornejo-Garcia, Maria Gabriela Canto, Miguel Blanca

P55 Subjects developing immediate responses to several NSAIDs can be selective with good tolerance to ASA

Natalia Blanca-Lopez, Diana Pérez-Alzate, Francisco Javier Ruano Perez, Inmaculada Doña, Maria Luisa Somoza, Inmaculada Andreu, Miguel Angel Miranda, Cristobalina Mayorga, Maria Jose Torres, Jose Antonio Cornejo-Garcia, Miguel Blanca, Maria Gabriela Canto

P56 Utility of low-dose oral aspirin challenges for diagnosis of aspirin exacerbated respiratory disease

Elina Jerschow, Teresa Pelletier, Zhen Ren, Golda Hudes, Marek Sanak, Esperanza Morales, Victor Schuster, Simon D. Spivack, David Rosenstreich

Poster Walk 7: NSAID 2 (P57–P65)

P57 Alternate regulation of cyclooxygenase-2 (COX-2) MRNA expression may predispose patients to aspirin-induced exacerbations

Renato Erzen, Mira Silar, Nissera Bajrovic, Matija Rijavec, Mihaela Zidarn, Peter Korosec

P58 Anaphylaxis to diclofenac: what about the underlying mechanism?

Leonor Carneiro-Leão, Fabrícia Carolino, Luís Amaral, Carmen Botelho, Eunice Dias-Castro, Josefina Cernadas

P59 COX-2 inhibitors: are they always a safe alternative in hypersensitivity to nonsteroidal anti-inflammatory drugs?

Luis Amaral, Fabricia Carolino, Eunice Castro, Josefina Cernadas

P60 Management of patients with history of NSAIDs reactions prior to coronary angioplasty

Mona Al-Ahmad, Tito Rodriguez

P61 Oral drug challenge with non-steroidal anti-inflammatory drug under spirometric control: clinical series of 110 patients

João Pedro Azevedo, Emília Faria, Beatriz Tavares, Frederico Regateiro, Ana Todo-Bom

P62 Prevalence and incidence of analgesic hypersensitivity reactions in Colombia

Pablo Andrés Miranda, Bautista De La Cruz Hoyos

P63 Recent endoscopic sinus surgery lessens reactions during aspirin challenge in patients with aspirin exacerbated respiratory disease

Teresa Pelletier, Waleed Abuzeid, Nadeem Akbar, Marc Gibber, Marvin Fried, Weiguo Han, Taha Keskin, Robert Tamayev, Golda Hudes, Simon D. Spivack, David Rosenstreich, Elina Jerschow

P64 Safe use of imidazole salycilate in a case of multiple NSAIDs induced urticaria-angioedema

Elisa Boni, Marina Russello, Marina Mauro

P65 Selective hypersensitivity reactions to ibuprofen—seven years experience

Marta Ferreira Neto

Poster Walk 8: Epidemiological methods (P66–P72)

P66 Allopurinol hypersensitivity: a 7-year review

Lise Brosseron, Daniela Malheiro, Susana Cadinha, Patrícia Barreira, J. P. Moreira Da Silva

P67 Antibiotic allergy labelling is associated with increased hospital readmission rates in Australia

Brittany Knezevic, Dustin Sprigg, Michelle Trevenen, Jason Seet, Jason Trubiano, William Smith, Yogesh Jeelall, Sandra Vale, Richard Loh, Andrew Mclean-Tooke, Michaela Lucas

P68 Experts’ opinions on severe cutaneous adverse drug reactions-report of a survey from the 9th international congress on cutaneous adverse drug reactions 2015

Roni P. Dodiuk-Gad, Cristina Olteanu, Wen-Hung Chung, Neil H. Shear

P69 HLA-A*31-positive AGEP with carbamazepine use and other severe cutaneous adverse drug reactions (SCARs) detected by electronic medical records screening

Sabine Müller, Ursula Amstutz, Lukas Jörg, Nikhil Yawalkar, Stephan Krähenbühl

P70 Patients with suspected drug allergy: a specific psychological profile?

Eunice Dias-Castro, Ana Leblanc, Laura Ribeiro, Josefina R. Cernadas

P71 Use of an electronic device and a computerized mathematic algorithm to detect the allergic drug reactions through the analysis of heart rate variability

Arantza Vega, Raquel Gutierrez Rivas, Ana Alonso, Juan Maria Beitia, Belén Mateo, Remedios Cárdenas, Juan Jesus Garcia-Dominguez

P72 Variation in ERAP influences risk for HLA-B*57:01 positive abacavir hypersensitivity

Rebecca Pavlos, Kaija Strautins, Ian James, Simon Mallal, Alec Redwood, Elizabeth Phillips

Poster Walk 9: DRESS/AGEP (P73–P81)

P73 A clinical case of DRESS syndrome in a child after administration of amoxicillin-clavulanic acid

Rita Aguiar, Anabela Lopes, Ana Neves, Maria Do Céu Machado, M. A. Pereira-Barbosa

P74 Acute generalized exanthematous pustulosis (AGEP) induced by mesalazine, reliable and oftenly used drug to treat inflammatory bowel disease

Ceyda Tunakan Dalgiç, Emine Nihal Mete Gökmen, Fatma Düsünür Günsen, Gökten Bulut, Fatma Ömür Ardeniz, Okan Gülbahar, Ali Kokuludag, Aytül Zerrin Sin

P75 Changes of blood plasmacytoid dendritic cells, myeloid dendritic cells, and basophils during the acute stage of drug reaction with eosinophilia and systemic symptoms (DRESS) and other drug eruptions

Shao-Hsuan Hsu, Yung-Tsu Cho, Che-Wen Yang, Kai-Lung Chen, Chia-Yu Chu

P76 Characterization of isoniazid/rifampicin-specific t-cell responses in patients with DRESS syndrome

Young-Min Ye, Gyu-Young Hur, Hae-Sim Park, Seung-Hyun Kim

P77 DRESS syndrome secondary to sulfasalazine with delayed TEN: a case presentation

Syed Ali, Michaela Lucas, Peter N. Hollingsworth, Andrew P. C. Mclean-Tooke

P78 Drug rash with eosinophilia and systemic symptoms (DRESS) features according to the culprit drug

Zohra Chadly, Nadia Ben Fredj, Karim Aouam, Haifa Ben Romdhane, Naceur A. Boughattas, Amel Chaabane

P79 Drug reaction with eosinophilia and systemic symptoms induced by allopurinol: not always easy to diagnose

Marina Lluncor Salazar, Beatriz Pola, Ana Fiandor, Teresa Bellón, Elena Ramírez, Javier Domínguez Ortega, Santiago Quirce, Rosario Cabañas

P80 Drug reaction with eosinophilia and systemic symptoms syndrome induced by two drugs simultaneously: a case report

Krasimira Baynova, Marina Labella, Manuel Prados

P81 The drug reaction with eosinophilia and systemic symptoms (DRESS) induced by the second-line antituberculosis drugs and Epstein–Barr virus infection

Agne Ramonaite, Ieva Bajoriuniene, Brigita Sitkauskiene, Raimundas Sakalauskas

Poster Walk 10: Miscellaneous drug hypersensitivity (P82–P91)

P82 A case of cycloserine-induced lichenoid drug eruption confirmed with a lymphocatye transformation test

Jae-Woo Kwon, Shinyoung Park

P83 Allergic reaction to topical eye drops: 5 years’ retrospective study in a drug allergy unit

Diana Silva, Leonor Carneiro Leão, Fabricia Carolino, Eunice Castro, Josefina Cernadas

P84 Allergy to heparins

Diana Perez-Alzate, Natalia Blanca-López, Maria Luisa Somoza Alvarez, Maria Garcimartin, Maria Vazquez De La Torre, Francisco Javier Ruano Pérez, Elisa Haroun, Gabriela Canto Diez

P85 Allopurinol-induced adverse drug reactions

Katinka Ónodi-Nagy, Ágnes Kinyó, Lajos Kemény, Zsuzsanna Bata-Csörgo

P86 Analysis of a population with immediate hypersensitivity to corticosteroids: an 11 year review

Joana Sofia Pita, Emília Faria, Rosa Anita Fernandes, Ana Moura, Nuno Sousa, Carmelita Ribeiro, Carlos Loureiro, Ana Todo Bom

P87 Anaphylaxis against mivacurium in a 12-months old boy at first-time exposure

Wolfgang Pfützner

P88 Antihistamine-exacerbated chronic spontaneous urticaria: a paradox?

Nadine Marrouche, Clive Grattan

P89 Anti-osteoporotic agents-induced cutaneous adverse drug reactions in Asians

Yu-En Chen, Chun-Bing Chen, Wen-Hung Chung, Yu-Ping Hsiao, Chia-Yu Chu

P90 Diagnosis of allergic reactions to eye drops

Maria Vazquez De La Torre, Natalia Blanca-Lopez, Diana Perez-Alzate, Maria Isabel Garcimartin, Francisco Javier Ruano, Maria Luisa Somoza, Elisa Haroun, Gabriela Canto

P91 Diagnostic approach in suspected hypersensitivity reactions to corticosteroids

Fabrícia Carolino, Eunice Dias De Castro, Josefina R. Cernadas

## Oral Abstracts

### O1 Functionally distinct HMGB1 isoforms correlate with physiological processes in drug-induced SJS/TEN

#### Daniel F. Carr^1^, Wen-Hung Chung^2^, Rosalind E. Jenkiins^1^, Mas Chaponda^1^, Gospel Nwikue^1^, Elena M. Cornejo Castro^1^, Daniel J. Antoine^1^, Munir Pirmohamed^1^

##### ^1^University of Liverpool, Liverpool, United Kingdom; ^2^Chang Gung Memorial Hospital, Taipei, Taiwan

**Correspondence:** Daniel F. Carr

*Clinical and Translational Allergy* 2016, **6(Suppl 3)**:O1

**Background**: Stevens Johnson syndrome (SJS) and toxic epidermal necrolysis (TEN) are serious, life threatening severe immune-mediated cutaneous reactions with mortality ranging from 10 to 30 %. The commonest causes are drugs. SJS/TEN is characterised by widespread epidermal detachment due to keratinocyte cell death. Increased concentrations of cytotoxic molecules may act as potential serum biomarkers of SJS/TEN. However, to date no mechanism based biomarker has been validated for diagnostic utility in this field. HMGB1 is a well-validated biomarker of cell death and inflammation. This study investigated whether HMGB1 represents a valid, utilisable biomarkers for drug-induced SJS/TEN.

**Materials and methods**: Serum samples from nevirapine-treated Malawian HIV patients (27 MPE, 12 DRESS, 12 SJS/TEN cases and matched tolerant controls) were analysed for total HMGB1 by ELISA. Novel mass-spectrometric protocols were also used to analyse post-translationally modified forms of HMGB1. In addition serum from 20 Taiwanese SJS patients (five carbamazepine, eight allopurinol, five phenytoin, two sulfamethoxazole) both during and post-reaction were analysed for HMGB1 isoforms.

**Results**: There was a significant elevation of mean total serum HMGB1 at time of reaction in patients with nevirapine-induced MPE (6.0 ng/ml), HSS (6.3 ng/ml) and SJS/TEN (15.9 ng/ml) compared to tolerant controls at weeks (1.3 ng/ml) (p < 0.001). Analysis of post-translationally modified isoforms of the HMGB1 in the different phenotypes (Fig. 1) showed patients with MPE and DRESS had elevation the acetylated form of HMGB1 which is a marker of innate immunity. By contrast, SJS/TEN patient sera contained comparable levels of acetylated HMGB1, but also had very high levels of the non-acetylated form, which is associated with cell death/tissue injury. The tolerant control patients had low levels of the unacetylated form. This pattern of HMGB1 isoform elevation was replicated in the Taiwanese SJS cohort. As patients recovered, the total HMGB1 concentrations went down, although there was still significant elevation of the sulphonyl (partially reduced) HMGB1 isoform which has no known immune function and may represent a marker of innate immunity returning to “steady state”.

**Fig. 1:**
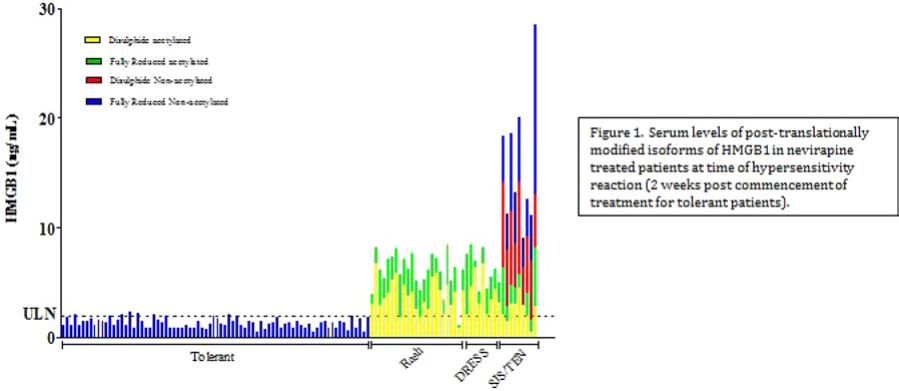
Serum levels of post-translationally modified isoforms of HMGB1 in nevirapine treated patients at time of hypersensitivity reaction (2 weeks post commencement of treatment for tolerant patients)

**Conclusions**: In conclusion, our data suggest that post-translationally modified HMGB1 may represent mechanism-based diagnostic and prognostic markers for drug-induced SJS/TEN. This needs to be studied in more patients.

**Keywords**: Stevens Johnson syndrome; HMGB1; Biomarker; Hypersensitivity

### O2 Hypersensitivity reactions to beta-lactams, does the t cell recognition pattern influence the clinical picture?

#### Natascha Wuillemin, Dolores Dina, Klara K. Eriksson, Daniel Yerly

##### University Hospital Bern, Bern, Switzerland

**Correspondence**: Dolores Dina

*Clinical and Translational Allergy* 2016, **6(Suppl 3)**:O2

**Background**: Worldwide, beta-lactam antibiotics can commonly cause hypersensitivity reactions (HR) with various clinical pictures from minor affections like maculopapular exanthema (MPE) and urticaria to severe cutaneous adverse reactions (SCAR) and anaphylaxis. Currently, two different concepts provide rational explanations how a drug can initiate a drug HR by activating human T cells—the hapten concept and the pharmacological interaction with immune receptor (p–i) concept. In this study, we investigated the relationship between the reactivity pattern of drug-reacting T cells found in the peripheral blood of allergic patients and their clinical picture.

**Materials and methods**: We expanded beta-lactams reacting T cells from drug allergic individuals, including patients with typically IgE mediated hypersensitivity reactions such as urticaria or anaphylaxis as well as patients with T cell mediated reactions such as MPE and SCAR. The drug-reacting T cells were analyzed in terms of their phenotype (CD4^+^/CD8^+^) and the recognition pattern of AMX, e.g. hapten or p–i.

**Results**: From patients with type I HR to amoxicillin, T cell clones (TCC) could be generated and analysed. They showed amoxicillin reactivity according to the hapten mechanism: antigenic complexes were stably presented and antigen presentation machinery was essential for T cell activation. TCC from patients suffering from MPE showed similar features. Three patients with DRESS to amoxicillin or ceftriaxone could be included. Drug reacting T cells from those patients showed exclusively reactivity according to pi-concept. Stimulatory antigenic complexes were not stably presented for T cell activation and addition of drug to TCC in the presence of antigenic presenting cells lead to immediate activation, measured by calcium intake.

**Conclusions**: We conclude that T cells from type I HR and MPE patients recognize beta-lactams according to the hapten mechanism. In contrast, in patients with SCAR, the p–i concept might also be relevant for beta-lactams recognition. Consequently, the current preclinical risk evaluation of new drugs to cause severe HR, which is solely based on their ability to form haptens, might be insufficient.

**Keywords**: Hapten; PI; Amoxcillin

### O3 Specific binding characteristics of HLA alleles associated with nevirapine hypersensitivity

#### Rebecca Pavlos^1^, Elizabeth Mckinnin^1^, David Ostrov^2^, Bjoern Peters^3^, Soren Buus^4^, David Koelle^5^, Abha Chopra^1^, Craig Rive^1^, Alec Redwood^1^, Susana Restrepo^6^, Austin Bracey^6^, Jing Yuan^7^, Silvana Gaudieri^8^, Mary Carrington^9^, David Haas^10^, Simon Mallal^10^, Elizabeth Phillips^10^

##### ^1^Murdoch University, Perth, Australia; ^2^University of Florida, Gainesville, USA; ^3^La Jolla Institute for Allergy and Immunology, La Jolla, USA; ^4^University of Copenhagen, Copenhagen, Denmark; ^5^University of Washington, Seattle, USA; ^6^Univesrity of Florida, Gainesville, USA; ^7^Boehringer Ingelheim Inc, Ridgefield, USA; ^8^Univesrity of Western Australia, Perth, Australia; ^9^Ragon Insitute, Cambridge, USA; ^10^Vanderbilt University, Nashville, USA

**Correspondence**: Rebecca Pavlos

*Clinical and Translational Allergy* 2016, **6(Suppl 3)**:O3

**Background**: Nevirapine (NVP) is a non-nucleoside reverse transcriptase inhibitor (NNRTI) associated with a hypersensitivity syndrome (HSR) in approximately 5 % of patients. Multiple class I/II HLA associations have been described in association with NVP hypersensitivity reaction (HSR) phenotypes. Based on the established models of drug HSR which highlight the significance of the HLA peptide binding groove for specific drug interactions, we compared NVP HSR-associated alleles across ethnic groups for similarities in peptide binding specificities and HLA binding pocket structure.

**Materials and methods**: HLA typing was performed on DNA from ClinicalTrials.gov NCT00310843. Univariate and multivariate analyses stratified for race were performed according to HLA class I/II alleles, MHCcluster groups and key HLA peptide binding groove amino acids.

**Results**: Examination of HLA allele peptide binding characteristics, together with structure of the B and F pockets in the peptide binding cleft identified a group of HLA-C alleles with common binding properties, and the same F pocket structure as HLA-C*04:01 that were predictive of cutaneous NVP HSR (HLA-C*04:(03/06/07), -C*05:(01/09), -C*18:01) (OR [95 % CI] 2.9 [1.6–5.23], p = 0.005). Similarly, a group of protective HLA-B alleles with a characteristic B pocket was identified (HLA-B*15:(01/12/24/25/27/32/35), -B*52:01) (OR [95 % CI] = 0.2 [0.07–0.5], p = 0.0003). HLA-DRB1 alleles DRB1*01:(01/02/03), DRB1*04:(04/05/08/10), -DRB1*14:02) which share the DRB1-P4 pocket were found to associate with cutaneous NVP HSR (OR [95 % CI] 2.15 [1.23–3.24], p = 0.0013). Molecular docking suggests that NVP is able to bind to the B pocket in both HLA-B and HLA-C as well as the P4 residues in HLA-DRB1.

**Conclusions**: The cutaneous phenotypes of NVP HSR associates with different HLA-C, HLA-B and DR-alleles respectively that share peptide binding characteristics and binding pocket structure. Models suggest that NVP may bind directly to multiple HLA within the antigen binding cleft in the site normally occupied by peptide. The identified HLA-NVP interactions will have consequences for peptide binding and T cell receptor recognition in NVP HSR.

**Keywords**: Nevirapine; HLA; Peptide binding groove

### O4 Do we need to measure total ige for the interpretation of analytical results of ImmunoCAP dnd 3gAllergy specific IgE?

#### Douwe De Boer^1^, Paul Menheere^1^, Chris Nieuwhof^2^, Judith Bons^1^

##### ^1^Central Diagnostic Laboratory, MUMC+, Maastricht, The Netherlands; ^2^Internal Medicine, MUMC+, Maastricht, The Netherlands

**Correspondence**: Douwe De Boer

*Clinical and Translational Allergy* 2016, **6(Suppl 3)**:O4

**Background**: Non-specific binding of IgE in in vitro IgE allergy tests contributes to false-positive results. For ImmunoCAP it is stated that very low levels of allergen specific IgE (sIgE) should be evaluated with caution when total IgE (tIgE) >1000 kU/l. For β-lactams and chlorhexidine sIgE the warning limit is 500 kU/l. For 3gAllergy no such alerts are known. These warnings imply to measure tIgE for an interpretation of analytical results of at least ImmunoCAP sIgE. Goal of this study is to verify such warning limits for ImmunoCAP as well as 3gAllergy.

**Materials and methods**: Relationship between sIgE and tIgE was investigated for ImmunoCAP (Thermo Fisher) and 3gAllergy (Siemens) penicillin V sIgE as well as for ImmunoCAP chlorhexidine sIgE. Ves versus 5 sIgE was taken as the 1000 kU/l limit control. All tests were performed according the manufacturers’ instructions. Because ImmunoCAP and 3gAllergy tIgE have a very strong correlation (r = 0.995), sIgE of both tests were graphically plotted against ImmunoCAP tIgE only. An iterative polynomial regression procedure, which excluded outliers when those after fitting were outside the 95 % confidence interval, was applied to check for the nature of a relationship.

**Results**: For ImmunoCAP penicillin V (r = 0.900) and chlorhexidine (r = 0.888) strong polynomial relations were observed, while for ImmunoCAP Ves versus five only very weak relations (r < 0.500) were noticed. For tIgE > 500 kU/l most of the penicillin V and chlorhexidine sIgE values were >0.10 kU/l and with increasing tIgE the number of sIgE values >0.35 kU/l increased. For 3gAllergy some results were >0.35 kU/l, but the majority of results was <0.10 kU/l and consequently insufficient data points were obtained for adequate regression. ImmunoCAP is based on high-capacity binding cellulose and at relatively high concentrations of tIgE, the frequency of possible false-positive results increases, especially for penicillin V and chlorhexidine sIgE. 3gAllergy is based on non-specified polymers attached to a bead, which is also subjected to false-positive results for penicillin V sIgE, but only at lower levels and frequency.

**Conclusions**: The warning limit of 500 kU/l of tIgE for ImmunoCAP penicillin V and chlorhexidine sIgE is valid and above the limit false-positive results are likely. For 3gAllergy penicillin V sIgE a limit is also needed but at a higher level. Consequently, for the respective tests we do need to measure tIgE for any sIgE result >0.10 kU/l.

**Keywords**: Total IgE; Penicillin; Chlorhexidine; ImmunoCAP; 3gAllergy

### O5 Neutrophil activation in systemic anaphylaxis: results from the multicentric NASA Study

#### Friederike Jonsson^1^, Luc De Chaisemartin^2^, Vanessa Granger^2^, Caitlin Gillis^1^, Aurelie Gouel^1^, Catherine Neukirch^2^, Fadia Dib^2^, Pascale Roland Nicaise^2^, Dan Longrois^2^, Florence Tubach^2^, Sylvie Martin^2^, Pierre Bruhns^1^, NASA Study Group^3^

##### ^1^Institut Pasteur, France, France; ^2^Hopital Bichat, France, France; ^3^Institut Pasteur & Hopital Bichat, France, France

**Correspondence**: Pierre Bruhns

*Clinical and Translational Allergy* 2016, **6(Suppl 3)**:O5

**Background**: Anaphylaxis is a severe systemic allergic reaction that can be life-threatening. In about 85 % of cases evidence for the activation of the classical anaphylaxis pathway involving IgE and IgE receptors can be detected. Recently, an alternative pathway involving IgG and IgG receptors (FcγRs) on neutrophils has been suggested by animal models. We hypothesized that such a mechanism may also exist in humans and studied this possibility in a multicentric prospective cohort of patients suspected of perioperative anaphylaxis to neuromuscular blocking agents (NMBA).

**Materials and methods**: Consecutive patients suspected of perioperative anaphylaxis (n = 86 cases) were recruited and paired with 86 control patients. Blood samples were collected for cases and controls promptly after anesthesia induction. Extensive allergological testing was performed 6–8 weeks after the reaction for cases. Circulating elastase, neutrophil extracellular traps (NETs), tryptase, histamine, and IgG and IgE anti-NMBA were measured by ELISA. FcγR expression on the major cell populations in the blood was analyzed by flow cytometry.

**Results**: We found higher circulating NETs and elastase levels during an anaphylactic reaction compared to controls. IgG anti-NMBA were found in both cases and controls, however for cases the IgG titer was associated with anaphylaxis severity. Finally, we show a significant decrease of FcγR expression specifically on neutrophils, pointing towards their engagement by immune complexes. This decrease not only correlated significantly with NET release but also with the severity of the anaphylactic reaction. Together, our results strongly suggest an activation of neutrophils by NBMA-IgG complexes during anaphylaxis.

**Conclusions**: We reveal for the first time the existence of an IgG-dependent neutrophil activation pathway during anaphylaxis in human. This additional mechanism opens potential applications in anaphylaxis diagnostics and treatment.

**Keywords**: Anaphylaxis; Drug; Curare; IgG; Neutrophils

### O6 Purpuric drug eruptions due to epidermal growth factor receptor tyrosine kinase inhibitors (EGFR-TKIs) for non-small-cell lung cancer (NSCLC): a clinic-pathological study of 32 cases

#### Kai-Lung Chen^1^, Shu-Ling Liao^1^, Yi-Shuan Sheen^1^, Yung-Tsu Cho^1^, Che-Wen Yang^1^, Jau-Yu Liau^2^, Chia-Yu Chu^1^

##### ^1^Department of Dermatology, National Taiwan University Hospital and National Taiwan University College of Medicine, Taipei, Taiwan, Taipei, Taiwan; ^2^Department of Pathology, National Taiwan University Hospital and National Taiwan University College of Medicine, Taipei, Taiwan, Taipei, Taiwan

**Correspondence**: Kai-Lung Chen

*Clinical and Translational Allergy* 2016, **6(Suppl 3)**:O6

**Background**: Epidermal growth factor receptor tyrosine kinase inhibitors (EGFR-TKIs) have been widely used to treat non-small-cell lung cancer (NSCLC). Skin toxicities related to EGFR-TKIs are common, such as acneiform eruptions, pruritus, xerosis, and paronychia. However, purpuric eruptions are rarely seen and only few cases reported. We conducted this study to classify the purpuric drug eruptions due to EGFR-TKIs (gefitinib, erlotinib, or afatinib) for NSCLC with clinic-pathological correlations.

**Materials and methods**: During January 2012 to August 2015, 32 patients were included in this study. We recorded the characteristic, lag period, and also the peripheral platelet counts while biopsy. Skin biopsies with tissue culture were undertaken in every patients. DIF studies were performed in most of them.

**Results**: We classified the clinical presentations into four different types: purpura only (n = 7, 21.9 %), eczema craquelé-like (n = 5, 15.6 %), pustulosis (n = 16, 50 %), and necrolytic migratory erythema (NME)-like patches (n = 8, 25 %). Different types could be presented on the same patient concomitantly. 93 % (15/16) of pustulosis type specimens grew *Staphylococcus aureus*, whereas only 25 % (5/20) among other types. Most of the histopathology showed parakeratosis, hypogranulosis, perivascular lymphocytic and neutrophilic infiltration, endothelial cell swelling and RBC extravasation. Typical leukocytoclastic vasculitis (LCV) was found in 13–29 % of patients. Most of the DIF showed negative finding. Most of the patients were responsive to one-week systemic cefazolin treatment with or without discontinuing the EGFR-TKI.

**Conclusions**: There are four types of the purpuric drug eruptions due to EGFR TKIs: purpura, eczema craquelé-like, pustulosis, and NME-like patches. No significant histopathological differences between each group, and less than one-third of patients presented typical LCV. *Staphylococcus aureus* was the most common pathogen identified. Most of the patients showed dramatic improvement by the treatment of systemic antibiotics, especially those with pustulosis type.

**Keywords:** EGFR-TKIs; Purpura; Vasculitis; Staphylococcus

## Poster Presentations: Poster Walk 1—Anaphylaxis (P01–P09)

### P1 Anaphylactic reactions during anaesthesia and the perioperative period

#### Rita Aguiar, Anabela Lopes, Natália Fernandes, Leonor Viegas, M. A. Pereira-Barbosa

##### Immunoallergology Department, Hospital de Santa Maria-Centro Hospitalar Lisboa Norte, Lisbon, Portugal

**Correspondence**: Rita Aguiar

*Clinical and Translational Allergy* 2016, **6(Suppl 3)**:P1

**Background**: Anaphylaxis incidence in the perioperative setting varies between 1:10,000 and 1:20,000. Although this value is yet to be determined in Portugal, an increase in the number of reactions has been reported. Clinical evaluation is important in order to identify risk factors and drugs that cause anaphylaxis, so that alternative options can be found.

The aim of this study was to characterize the reactions of patients (pts) with perioperative anaphylaxis and for conducting medical techniques requiring sedation.

**Materials and methods**: Retrospective analysis of medical records of 57 pts with perioperative anaphylaxis and anaphylaxis undergoing medical procedures with sedation, observed in the immunoallergologic outpatient clinic (2009–2015).

The diagnostic investigation was carried out 6–8 weeks after the reaction, included detailed medical history, specific IgE assay to betalactams and latex, skin tests (ST) with the culprit drug and evidence of provocation when necessary, according to the recommendations of the SFAR/ENDA.

**Results**: We studied 56 pts (41 females) with mean age of 50 ± 18 years.

Regarding the severity of anaphylaxis and according to Mertes classification, 24 cases (42.8 %) had stage I reaction and 24 cases (42.8 %) had II reaction, 7 cases (12.5 %) grade III and 1 case (1.8 %) grade IV.

An IgE-mediated mechanism has been established in 34 pts (60.7 %). The major etiologic agents causing IgE-mediated reactions were muscle relaxants in seven pts (12.5 %), antibiotics in six cases (10.7 %; four cefazolin, one aminopenicillin, one ciprofloxacin), metamizole in three pts (5.4 %), two pts latex (3.6 %), seven pts (12.5 %) reacted with less representative agents.

On 10 pts (17.8 %) drugs responsible for the reactions were associated with non-IgE-mediated mechanisms, anti-inflammatory (NSAIDs) are the most frequent agents (five pts).

In 14 pts (25 %) it was not possible to determine a pharmacological aetiology of the reaction.

**Conclusions**: More than half of perioperative events (60.7 %) have an IgE-mediated mechanism. Muscle relaxants, antibiotics and patent blue dye were the most frequently identified agents. In 17.8 % of the reactions was involved a non-IgE-mediated mechanism, namely NSAIDs. It is important to determine the aetiology of perioperative reactions for guidance in future surgery, either because the identified agents are often used outside the perioperative setting.

**Keywords**: Anaphylatic reactions; Anaesthesia

### P2 Anaphylaxis to chlorhexidine: is there a cross-reactivity to alexidine?

#### Antonia Bünter^1^, Nisha Gupta^2^, Tatjana Pecaric Petkovic^1^, Nicole Wirth^1^, Werner J. Pichler^1^, Oliver Hausmann^3^

##### ^1^ADR-AC GmbH, Bern, Switzerland; ^2^Teleflex Incorporated, Bern, Switzerland; ^3^Dep. of Rheumatology, Immunology and Allergology, University Hospital and University of Bern, Bern, Switzerland

**Correspondence**: Antonia Bünter

*Clinical and Translational Allergy* 2016, **6(Suppl 3)**:P2

**Background**: Chlorhexidine (CHX) and its diacetate derivative (CHA) are two forms of the same disinfectant for skin and mucosal surfaces as well as medical devices. CHX/CHA are bivalent compounds with biguanide groups and chlorophenyl endings. CHX/CHA can cause IgE-mediated anaphylaxis. Alexidine (ALX), a related biguanide without aromatic end groups, has similar bactericidal properties and represents a potential substitute for CHX/CHA. The allergic potential of ALX is unknown.

**Materials and methods**: We investigated whether patients sensitized to CHX/CHA also react with ALX. We used blood of CHX/CHA-allergic donors for basophil activation testing (BAT for CD63 and CD203a as activation markers) with CHA and ALX. In addition, we performed inhibition assays with CHA, chlorguanide (CG) and ALX using a commercial IgE assay for CHX (ImmunoCAP, ThermoFisher Scientific, Uppsala).

**Results**: 13 patients from a single tertiary care center with allergic reactions to CHX within the last 8 years were included. 9/13 patients had still elevated CHX-specific IgE (>0.35 kU/l), although mostly substantially lower compared to the time of anaphylaxis. In 6/13 patients with CHX-specific IgE >0.7 kU/l CAP inhibition studies were performed. CG showed a strong inhibitory effect in 6/6 and CHA in 3/6 tested sera. ALX induced partial inhibition of CHX positivity in 2/6 sera but at much higher concentrations compared to CHA. 3/13 patients showed a positive BAT with CHA, one of them with additional positivity to ALX.

**Conclusions**: Both, CAP inhibition studies as well as BAT analysis show that patients with documented CHX/CHA allergy do not or only partially react with ALX in these IgE-based assays. Based on this limited cross-reactivity of CHX/CHA and ALX, ALX may be potential alternative for CHX-allergic patients. More patients need to be included to substantiate this assumption. An ALX-specific IgE assay would help to differentiate CHX/CHA- and ALX-specific sensitization.

### P3 Cefotaxime-induced severe anaphylaxis in a neonate

#### Mehtap Yazicioglu^1^, Pinar G. Ozdemir^1^, Gokce Ciplak^2^, Ozkan Kaya^3^

##### ^1^Trakya University Department of Pediatric Allergy, Edirne, Turkey; ^2^Trakya University Department of Pediatrics, Edirne, Turkey; ^3^Trakya Hospital, Edirne, Turkey

**Correspondence**: Mehtap Yazicioglu

*Clinical and Translational Allergy* 2016, **6(Suppl 3)**:P3

**Background**: Anaphylaxis is defined as a serious generalized allergic or hypersensitivity reaction that is rapid in onset and might cause death. It is very rare in infancy. In this case report, we present a neonate who developed anaphylaxis during infusion of first dose of cefotaxime.

**Report**: A-1 month infant was referred to our Pediatric Emergency Department because of anaphylactic reaction with rash, cyanosis and respiratory arrest developed after first dose of cefotaxime treatment. She was intubated immediately, then extubated after 10 min. Physical examination on admission showed prolonged expirium bilaterally, and rales at the right lung base. Other findings were unremarkable. She was hospitalized and treatment with salbutamol nebules (0.15 mg/kg/dose, q 6 h) was started. Vital findings were observed closely. On the 4th day of admission, before discharge, she was consulted with our pediatric allergy department. She was born full term by Cesarean delivery, weighing 3180 g. Her personal and family history was unremarkable, except her mother described rash and swelling of the lips and face after spraying cologne. Laboratory investigations on admission: CBC with white blood cell differential was within normal range; C-reactive protein (CRP): 1.35 mg/dl (0–0.34); serum total IgE: 2.4 U/ml (0–170). Serum tryptase: 17.7 ng/ml (on the 4th day of admission); serum tryptase 12.3 ng/ml (after 2 months). Skin prick tests with cefotaxime (2 mg/ml), and cefotaxime (10 mg/ml) were both negative. We did not perform intradermal tests and drug provocation test with cefotaxime. The patient was discharged to be followed with prescription of epinephrine autoinjector to use in case of anaphylactic emergency, and the family was educated about about signs and symptoms of anaphylaxis and about when and how to use the EpiPen.

**How this report contributes to current knowledge**: Anaphylaxis in newborn period is very rare. Our case was interesting that to our knowledge this is the first case of anaphylaxis in a newborn induced by first dose of an antibiotic. We wished to attract attention that severe drug reactions can also be seen in early life. 

### P4 Clinical features and diagnosis of anaphylaxis resulting from exposure to chlorhexidine

#### Peter John Cooke

##### Auckland City Hospital, Auckland, New Zealand

**Correspondence**: Peter John Cooke

*Clinical and Translational Allergy* 2016, **6(Suppl 3)**:P4

**Background**: The Auckland Anaesthetic Allergy Clinic is jointly provided by the Departments of Anaesthesia and Immunology. All patients referred following perioperative anaphylaxis are skin prick tested with chlorhexidine 2 %. This review describes the clinical features of the cases in which a diagnosis of allergic anaphylaxis to chlorhexidine was made during the period January 2012 to December 2015.

**Materials and methods**: Patient data and clinic test results were archived on a simple Excel spreadsheet. These were reviewed by the author and a chart review of the chlorhexidine anaphylaxis cases was undertaken.

**Results**: A total of 14 patients were diagnosed with chlorhexidine allergy during the period (11 male and 3 female).

The following manifestations of anaphylaxis were documented: hypotension (12 patients), tachycardia (9), flushing (7), facial swelling (4), urticaria (3), bronchospasm (2), piloerection (2), agitation (1), generalized swelling (1). Four patients had CPR during their anaphylaxis, nine patients had a grade 3 reaction and one patient had a grade 1 reaction. Twelve of the 14 patients received adrenaline. Anaphylaxis followed the insertion of a chlorhexidine impregnated central venous line in seven cases, urethral catheterization with chlorhexidine containing gel in three and following topical exposure only in four cases.

Skin testing was performed 30–130 days after the anaphylaxis event.

All of the patients diagnosed with chlorhexidine allergy developed a wheal >3 mm after a s prick test with 2 % chlorhexidine (range 3–20 mm). The specific IgE for Chlorhexidine was obtained in 11 of the 14 cases and in 10 of the 11 it was elevated.

During the same period 219 other patients were seen at our clinic and all received skin prick tests for chlorhexidine 2 %. All of these patients had completely negative tests with a no wheal and no flare.

**Conclusions**: This data shows that patients with chlorhexidine anaphylaxis demonstrate typical signs but that hypotension is the most common manifestation. Convincing skin testing results along with specific IgE testing and the clinical history provided the basis of the diagnosis. Chlorhexidine is used in our region for skin preparation prior to anaesthetic and surgical procedures and four anaphylaxis cases resulted from topical exposure to chlorhexidine.

Our data also suggests that a skin prick test with 2 % chlorhexidine in alcohol is a satisfactory method of skin testing.

**Keywords**: Chlorhexidine; Skin prick testing; Specific IgE; Anaphylaxis

### P5 Drug-induced anaphylaxis: five-year single-center survey

#### Inês Mota, Ângela Gaspar, Filipe Benito-Garcia, Marta Chambel, Mário Morais-Almeida

##### Immunoallergy Department, CUF Descobertas Hospital, Lisbon, Portugal

**Correspondence**: Inês Mota

*Clinical and Translational Allergy* 2016, **6(Suppl 3)**:P5

**Background**: Drug-induced anaphylaxis (DIA) is the most common cause of fatal anaphylaxis. Anaphylaxis related to nonsteroidal anti-inflammatory drugs (NSAID) is typically drug-specific or class-specific, as well as with beta-lactams (BL) antibiotics (AB). Although skin testing and drug provocation tests (DPT) can confirm the diagnosis, in severe cases the diagnosis is mostly based on clinical history. The aim of this study was to characterize patients (pts) with DIA and their drug allergy work-up.

**Materials and methods**: Systematic review of all pts with clinical history compatible with DIA reported to our drug allergy center in the last 5 years. All pts were investigated according to ENDA/EAACI recommendations, through skin testing and in vitro tests (whether standardized tests available) and DPT (when indicated).

**Results**: A total of 114 pts were included: mean age 41.5 (SD ± 16.8) years, 10 % <18 years, 68 % female, 72 % atopic and 23 % had asthma. Median age of first anaphylactic episode was 36.5 years [1;74], and 19 pts had recurrent DIA. The main causes were NSAID (50 pts) [acetylsalicylic acid (15), ibuprofen (13), metamizol (13), diclofenac (9), paracetamol (3), etodolac, ketorolac and clonixin (one each)] and AB (46 pts) [BL (37), quinolones (4), macrolides (3), fosfomycin (1) and minocycline (1)]. Other drug agents found: neuromuscular blocking drugs (five pts), *proton pump inhibitors* (five pts)*, carboplatin* (three pts), corticosteroids (two pts), *local* anesthetics (two pts), ranitidine, midazolam and patent blue (1pt each). There was a predominance of mucocutaneous manifestations (96 %), followed by respiratory (80 %) and cardiovascular (45 %) symptoms. In 25 % of pts the reaction occurred in hospital setting and 12 % had intraoperative anaphylaxis. DIA was supported in 72 pts (63 %), through skin tests in 62 and the remaining by in vitro tests or DPT. Considering the severity of reactions and the lack of standardized tests for some drugs, patients whose DIA was based on clinical history were successfully challenged with alternative drugs.

**Conclusions**: NSAID and AB were responsible for the majority of DIA. Anaphylactic reactions were reported at any age. The heterogeneity of mechanisms involved, the severity of clinical reactions and the lack of standardized in vivo and/or in vitro tests do not allow to confirm the diagnosis in all cases. Patients with DIA should be evaluated in specialized centers in order to perform accurate diagnosis, to prevent recurrence and to find safe alternatives.

**Keywords**: Anaphylaxis; Drug allergy; NSAID; Antibiotics

### P6 Intraoperative severe anaphylactic reaction due to patent blue v dye

#### Luis Marques, Eva Alcoceba, Silvia Lara

##### Hospitals Universitaris Santa Maria - Arnau de Vilanova, Lleida, Spain

**Correspondence**: Luis Marques

*Clinical and Translational Allergy* 2016, **6(Suppl 3)**:P6

**Background**: Intraoperative anaphylactic reactions are a diagnostic challenge. The chronology of the administration of the multiple drugs and the beginning of the reaction are important in identifying the culprit drug.

Patent blue V is a well known cause of perioperative anaphylaxis

This dye is a member of the triarylmethane family, which also includes isosulfan blue and methylene blue. Is used for staging breast cancer, identifying sentinel lymph nodes. The frequency of reactions is around 0.24–1.1 %. It can be also found in food (food additive E-131) and cosmetics.

**Report**: We describe the case of a woman 65 years-old with a ductal carcinoma of the left breast, who suffered hypotension (85/60), bronchospasm with hypoxemia, urticaria, angioedema of the face, tongue and epiglottis after the administration through the nipple of colorant patent blue V. Anaesthetic induction was done with fentanyl, propofol and rocuronium. Adrenaline, bronchodilators, antihistamines, corticosteroids and vasopressive drugs were administered, being admitted in the ICU. The levels of tryptase were 8.5 µg/ml 20 min after the beginning of the reaction, 16.6 µg/ml at 2 h and 3.23 µg/ml at 48 h.

One month after the reaction cutaneous tests were done, being positive for patent blue V (intradermal reaction at 1/10) and negative for suxamethonium, cis-atracurium, rocuronium, fentanyl, propofol, midazolam, povidone-iodine and latex.

It was the first time the patient received this dye or any similar dye as a drug.

Intraoperative anaphylactic shock due to allergy to patent blue V was diagnosed.

The future use of this dye was prohibited and an advise to avoid stuff which contains this product was given to the patient.

A suspicion of sensitization through foods or cosmetics is possible as the patient reacted the first time she received this drug.

**How this report contributes to current knowledge**: This case confirms previous descriptions of reactions with patent blue V: reaction with the first exposition and severe allergic reactions, with hypotension and raise in tryptase levels. Cutaneous test are useful in the diagnostic and standardized concentrations have been described by ENDA.

**Consent**: Written informed consent was obtained from the patient for publication of this abstract and any accompanying images.

### P7 Kounis syndrome in the setting of anaphylaxis to diclofenac

#### Leonor Carneiro-Leão, Carmen Botelho, Eunice Dias-Castro, Josefina Cernadas

##### Serviço de Imunoalergologia, Centro Hospitalar de São João, Porto, Portugal

**Correspondence**: Leonor Carneiro-Leão

*Clinical and Translational Allergy* 2016, **6(Suppl 3)**:P7

**Background**: Kounis Syndrome (KS), the occurrence of acute coronary events as consequence of allergic or hypersensitivity reactions has been described for years. It is still relatively unknown and consequently underdiagnosed, leading to inadequate treatment and subsequent morbidity. There are three subtypes of KS: type I which occurs in patients without predisposing cardiovascular factors; type II which occurs in patients with cardiovascular risk factors; and type III by stent thrombosis.

**Report**: A 55 year old man, a smoker with type II Diabetes, was admitted to our hospital for myocardial infarction (MI) after taking two pills of diclofenac 75 mg for left leg pain. He complained of immediate generalized pruritus, malaise, constrictive radiating chest pain, dyspnea, dizziness and sweating. He was assessed by the mobile medical team on site as being agitated, hypotensive (BP: 96/74 mmHg) and with generalized wheezing on chest auscultation. Aggressive fluid resuscitation, nebulized salbutamol and IV corticosteroids improved his status on route to the ER; he was also treated with acetylsalicylic acid 250 mg and sublingual isosorbide dinitrate 5 mg. Epinephrine was not given. ECG confirmed NSTEMI with an elevated troponin I (0.59 ng/ml). Serum tryptase was not measured. He was admitted to the Coronary ICU and cardiac catheterization showed mild coronary artery disease. He recalled a previous reaction to diclofenac, with immediate generalized pruritus. The patient recovery was uneventful. He was then referred to our Drug Allergy Unit. Oral challenge with meloxicam 15 mg was negative and he was instructed to avoid NSAID’s other than meloxicam.

**How this report contributes to current knowledge**: MI in the setting of anaphylaxis is underreported. Clinicians should be aware of this possible complication even in patients without cardiovascular risk factors in order to diagnose and treat it early. Also, the WAO Anaphylaxis guidelines recommend a minimum 4 h observation period after anaphylaxis, 8–10 h if there is respiratory or cardiovascular compromise during the reaction. This allows not only for detection and treatment of biphasic reactions, but also of secondary cardiovascular events.

**Consent**: Written informed consent was obtained from the patient for publication of this abstract and any accompanying images.

### P8 Perioperative anaphylaxis audit: Royal Melbourne Hospital

#### Katherine Nicholls^1^, William Lay^2^, Olivia Smith^2^, Christine Collins^1^, Gary Unglik^1^, Kymble Spriggs^1^, Priscilla Auyeung^1^, Jeremy McComish^1^, Jo A. Douglass^1^

##### ^1^Department of Immunology and Allergy, The Royal Melbourne Hospital, Parkville, Australia; ^2^Department of Medicine, University of Melbourne, Parkville, Australia

**Correspondence**: Katherine Nicholls

*Clinical and Translational Allergy* 2016, **6(Suppl 3)**:P8

**Background**: Perioperative anaphylaxis (PA) is a medical emergency, with potential for mortality. Skin testing (ST) of agents used in the perioperative period is considered the gold standard for the identification of likely causative agents [1]. Neuromuscular blocking agents (NMBA) (58 %), antibiotics (12–15 %) and latex (16–19 %) are the most commonly implicated causative agents [1, 2]. Chlorhexidine allergy is also described although its prevalence is not well established and likely underreported [3, 4].

We conducted a retrospective audit of all patients who underwent ST and specific IgE testing (SpIgE) for investigation of a perioperative allergic event. We sought the proportion of patients who had a likely agent detected and the prevalence of likely causative agents in our cohort.

**Materials and methods**: Medical histories were reviewed for all patients referred for PA who underwent testing in our centre over a 2-year period from September 2013 to August 2015. Data collected included severity grading [5], acute elevation in Mast cell tryptase (MCT) (>12 ng/ml or >135 % basal level), and causative agent indicated by the presence of a positive ST or SpIgE to NMBA, latex, antibiotics or chlorhexidine. Results are expressed as n (%) and comparisons performed by Fisher’s exact test.

**Results**: Of 47 patients identified during this period, ST was positive in 22 (47 %), with NMBA and beta-lactam antibiotics accounting for 10 (42 %) and 7 (29 %) of positive results respectively. Of the 27 patients with MCT results, 14/19 (73 %) patients with an acute MCT rise had positive skin or SpIgE tests, compared with only 1 (13 %) of 8 patients with no reported MCT rise (P < 0.01). Over the same period, four patients (18 %) had positive SpIgE to chlorhexidine (range 0.43–55.8kUa/l). Reactions graded as severe (Grade 3) were associated with an increased proportion of positive ST (16/31, 52 %) compared with Grades 1 and 2 (6/16, 38 %), however this result did not reach statistical significance.

**Conclusions**: The prevalence of causative agents reflects current literature, with an increased proportion of reactions to antibiotics, and decreased proportion to latex. Our audit indicated a significantly higher proportion of positive ST in those with acutely elevated MCT. In our cohort, chlorhexidine appeared to be a common allergen.

**References**

1. Mertes, et al. JACI 2011;128:366–73.
2. Ebo, et al. Allergy 2007;62:471–87.
3. Garvey, et al. JACI 2007;120:409–15.
4. Calogiuri, et al. J Allergy Ther. 2013;4.
5. Brown, et al. JACI 2004;114:371–6.

### P9 Recurrent peri-operative anaphylaxis: a perfect storm

#### Jonny G. Peter, Paul Potter

##### University of Cape Town, Cape Town, South Africa

**Correspondence**: Jonny G. Peter

*Clinical and Translational Allergy* 2016, **6(Suppl 3)**:P9

**Background**: Allergy work-up to identify the causative agent in patients experiencing peri-operative anaphylaxis is challenging. Patients have multiple exposures over a short time period; Paper-based records from remote hospitals are often unavailable to the allergist; and diagnostic testing for many drugs is either unavailable or sub-optimal.

**Report**: A 56-year-old man with debilitating osteoarthritis required hip replacements in order to work. He had no chronic medical co-morbidities, but labeled penicillin allergy following a childhood reaction during prolonged antibiotics for osteomyelitis. He was referred for testing after three operations in a remote private South African hospital. Peri-operative anaphylaxis, confirmed with serial tryptase measurement, occurred in the 1st and 3rd surgeries. Possible offending agents included: propofol, midazolam, bupivacaine, fentanyl, clindamycin and cyclokapron. Apparently, in the uneventful 2nd surgery, the drugs were the same except cyclokapron was omitted. Cleaning agents used were unknown. *In vitro* specific IgE testing was negative to latex but elevated (5.49 kUA/l) for chlorhexidine; Skin testing was positive with chlorhexidine and intradermal testing generated systemic symptoms and required treatment. CAST ELISA testing was negative to propofol and bupivacaine. Clindamycin in vitro testing was unavailable and in vivo testing is not recommended. The likely offending agent was chlorhexidine, and the patient was anxious to return to work, thus, although each anesthetic chart was not available for detailed review, repeat surgery proceeded. Unfortunately, he experienced recurrent anaphylaxis despite a chlorhexidine free theatre. Several months later the anesthetic charts from all four operations were acquired, and it was clear that an additional offending agent was likely clindamycin. Avoidance of both clindamycin and chlorhexidine resulted in a safe surgery. Subsequent testing allowed the patient to be ‘de-labeled’ as penicillin allergic.

**How this report contributes to current knowledge**: Dual sensitivity is described in about 2 % of peri-operative anaphylaxis cases. Thus, even if a possible causative agent is identified, it remains mandatory a conduct a detailed review of all anesthetic charts; however, this can pose a major challenge in countries were records remain paper-based. The use of alternative drugs in patients carrying a penicillin allergy label can carry significant morbidity.

**Consent**: Written informed consent was obtained from the patient for publication of this abstract and any accompanying images.

## Poster Walk 2: DH regions and patient groups (P10–P19)

### P10 A rare presentation of amoxicillin allergy in a young child

#### Fabrícia Carolino, Eunice Dias De Castro, Josefina R. Cernadas

##### Serviço de Imunoalergologia, Centro Hospitalar São João E.P.E., Porto, Portugal

**Correspondence**: Fabrícia Carolino

*Clinical and Translational Allergy* 2016, **6(Suppl 3)**:P10

**Background**: Fixed drug eruptions (FDE) are characterized by well-demarcated skin and/or mucosal lesions beginning a few minutes to several hours after drug exposure and that reappear at the exact location on re-exposure to the offending drug. Aminopenicillins are among those drugs most commonly associated with FDE. Non-pigmenting FDE (NPFDE) is an FDE type that leaves no residual pigmentation.

**Report**: The authors report the case of a non-atopic 5 years-old boy, presenting two reproducible episodes of cutaneous lesions (erythematous pruritic plaques) located to the genital area, occurring a few hours after medication with amoxicillin for upper airways infection. The antibiotic was changed to a non-beta-lactam drug with progressive symptoms resolution, leaving no residual skin pigmentation. There was a previous exposure to amoxicillin with tolerance. After the second episode, the child has already been medicated with a second generation cephalosporin (cefaclor) and tolerated this drug. Following the first evaluation in our Allergy Department, the child was assessed in the Drug Allergy Unit. No skin tests (intradermal with late reading) were performed due to age-related constraints. A controlled re-challenge with oral amoxicillin in the age-recommended intake-dose was performed. Five hours after the end of oral challenge, the child began to develop a cutaneous exanthema and returned to the hospital for medical assessment as it was recommended. On physical examination, we observed swelling and erythematous plaques affecting the inferior part of penis, the scrotum and the perianal region; the child’s mother confirmed that the lesions presented the same location as that of the two previous episodes. Taking these findings into account, the final diagnosis was multiple fixed drug eruption. The child was treated with oral antihistamine and corticosteroid, with complete resolution in 3 days.

**How this report contributes to current knowledge**: To the authors’ knowledge, this is the third case reported of this type of drug eruption in the paediatric age.

**Consent**: Written informed consent was obtained from the patient for publication of this abstract and any accompanying images.

### P11 Adverse drug reactions in children: antibiotics or virus?

#### Ana Sofia Moreira^1^, Carmo Abreu^2^, Eva Gomes^2^

##### ^1^Centro Hospitalar Vila Nova Gaia e Espinho, Vila Nova Gaia, Portugal; ^2^Centro Hospitalar do Porto, Porto, Portugal

**Correspondence**: Ana Sofia Moreira

*Clinical and Translational Allergy* 2016, **6(Suppl 3)**:P11

**Background**: About 10 % of Portuguese children report to have had at least one adverse reaction to drugs and 6 % to be allergic to at least one drug, being the antibiotics the most frequent medication implicated. The diagnostic investigation reveals that in 90 % of cases drug hypersensitivity is not confirmed, what is thought to be related to the high prevalence of infectious/viral rashes in childhood. Our aim was to evaluate the main reasons for referral to our pediatric drug allergy clinic, characterize the reactions mentioned by patients, particularly with regard to their sazonality and to analyse the results of the diagnostic investigation performed.

**Materials and methods**: Retrospective analysis of medical records from the pediatric patients followed in our department, in the last 6 years, due to suspected drug hypersensitivity.

**Results**: We studied 364 children of whom 200 (55 %) were male. The median age at the time of the suspected reaction was 3.5 years (6 months–17 years). Antibiotics were the suspected drug in 84 % of cases (n = 305). In 75 % (n = 274) of patients the symptoms reported were only cutaneous (maculopapular eruptions or urticaria) and in 72 % (n = 262) of cases the reaction was nonimmediate. Drug hypersensivity was confirmed in 10 patients (3 %). The month in which the drug reaction occurred was identified by 51 % (n = 184) of patients. Sixty percent of the reactions occurred during winter–spring months. Viral serologies were performed on 74 patients who reported a recent reaction and positive results for at least one of the viruses studied were obtained in 14 patients (19 %—IgM) and 48 patients (65 %—IgG).

**Conclusions**: The majority of children studied due to suspected drug hypersensitivity reported cutaneous symptoms and nonimmediate reactions. Most reactions occurred before 3 years of age, which according to the literature, concerns the age when infectious rashes are more frequent. We verified a seasonal pattern in the occurrence of the suspected drug reactions that is similar to that described for the most common viral infections in childhood, while the peak for antibiotic consumption has been reported in autumn. Confirmation of hypersentisitivity to the suspected drug was possible in 10 of 364 patients, while a possible viral etiology was documented in 14 of 74 patients. Our findings reinforce the idea that many of the cutaneous reactions which motivate the study in our department are probably caused by infections and not by drug hypersensitivity.

**Keywords**: Children; Drug reactions

### P12 Allergic reactions in invasive medical procedures

#### Bárbara Kong Cardoso, Elza Tomaz, Sara Correia, Filipe Inácio

##### Hospital de S.Bernardo - Centro Hospitalar de Setúbal, Setúbal, Portugal

**Correspondence**: Bárbara Kong Cardoso

*Clinical and Translational Allergy* 2016, **6(Suppl 3)**:P12

**Background**: Surgery associated allergic reactions have been extensively studied and culprit agents have been pointed on several reports. Nevertheless last decade changes in pharmacological protocols resulted in the increase of possible implicated drugs. In the other hand, other invasive procedures (diagnostic/therapeutic) have been increasing in frequency and are also related to allergic events. Our aim was to seek for new agents involved in perioperative allergy as well as characterize reactions occurring due to other invasive procedures

**Materials and methods**: We reviewed the medical records of 28 patients referred to our clinic for allergic reaction associated to an invasive procedure in the last 2 years regarding administered drugs, type of reaction and results of the allergological workup.

**Results**: In our study group, 21 were female and 7 male, mean age was 54.7 years (2–84).

Fourteen were studied for procedures with general anesthesia related adverse events: six anaphylatic reactions, six urticaria/angioedema, one bronchospasm and one hypotension. Positive relevant results were obtained in 13 patients: one skin prick test (SPT) to metamizol, two intradermal skin tests (IDT) to midazolam, one to tramadol, three to ondansetron, one to rocuronium, one to atracurium, two to vecuronium, two to metamizol, one basophil activation test (BAT) to metamizol. One patient was positive to both to atracurium and vecuronium and one patient had no positive tests.

In nine patients reactions were associated to invasive procedures with local anesthesia. Positive results were one IDT and BAT to verapamil (anaphylaxis) and one patch test to iodixanol (maculopapular rash) in two patients submitted to coronary angiography. Six patients with adverse reactions during dental treatment and one during a carpal tunnel surgery had no positive tests.

Five patients had been submitted to procedures without use of any anesthetic: one with local erythema after an esteroid intra-articular infiltration had a positive patch test to betamethasone dipropionate; one with an anaphylactic reaction during a colonoscopy had a positive BAT to metamizol used as analgesic; one with acute urticaria after contrast injection for a thyroid TC had IDT positive to iomeprol. Two patients had a negative workup.

**Conclusions**: Comparing to previous published series ondansetron seems to be emerging as an important agent in perioperative allergy.

Invasive procedures other than major surgeries are associated to allergic reactions both immediate and non-immediate, some of them being severe.

**Keywords**: Perioperative allergy; Invasive medical procedures; Drug hypersensitivity

### P13 Antibiotic allergy in children: room for improvement

#### Annabelle Arnold^1^, Natasha Bear^2^, Kristina Rueter^3^, Grace Gong^4^, Michael O’Sullivan^5^, Saravanan Muthusamy^1^, Valerie Noble^1^, Michaela Lucas^6^

##### ^1^Department of Immunology, Princess Margaret Hospital, Perth, Australia; ^2^Telethon Kids Institute, Department of Clinical Research and Education, Princess Margaret Hospital, Perth, Australia; ^3^Department of Immunology, Department of Clinical Research and Education, Princess Margaret Hospital, Telethon Kids Institute, Perth, Australia; ^4^Department of Immunology, PathWest Laboratory Medicine WA, Princess Margaret Hospital, Perth, Australia; ^5^Department of Immunology, Princess Margaret Hospital, PathWest Laboratory Medicine WA, Fiona Stanley Hospital, School of Pathology and Laboratory Medicine, University of Western Australia, Perth, Australia; ^6^Department of Immunology, Princess Margaret Hospital, Sir Charles Gairdner Hospital, PathWest Laboratory Medicine WA, School of Medicine and Pharmacology, School of Pathology and Laboratory Medicine, University of Western Australia, Institute of Immunology and Infectious Diseases, Murdoch University, Perth, Australia

**Correspondence**: Annabelle Arnold

*Clinical and Translational Allergy* 2016, **6(Suppl 3)**:P13

**Background**: Beta-lactam antibiotics remain one of the most effective treatments of bacterial infections and are the most frequently prescribed antibiotic in children. Allergic reactions to these antibiotics are common. Nevertheless, strategies for peadiatric antibiotic allergy testing and management remain poorly defined, including issues around the need for skin testing prior to oral provocation challenges (OPC; 2-dose) and the value of prolonged courses with the culprit antibiotic after the successful administration of an initial supervised dose.

**Materials and methods**: To address these issues, we performed a retrospective cross-sectional analysis of children (6 months–16 years) with an antibiotic allergy label who presented to a tertiary pediatric hospital in Western Australia from 2006 to 2015. Data collection included results of skin prick (SPT), intradermal testing (IDT) and OPCs, outcome of 5-day antibiotic courses, type of initial reaction, confounding illnesses and co-existing allergies. The data was analysed using Mann–Whitney U for continuous data and Fishers exact test for categorical data.

**Results**: We performed 207 beta lactam antibiotic tests in 172 children. In 82 (39.6 %) cases OPCs were performed without preceding skin testing (Group 1); three OPCs (3.7 %) were unsuccessful with one child with anaphylaxis. In 125 (60.4 %) cases skin testing was performed (Group 2). Of these, 22 cases (17.6 %) were positive by SPT or IDT; these children were deemed to have confirmed antibiotic allergy. The remaining 103 cases proceeded to OPC; 4 (3.8 %) reacted to OPC, including two children with anaphylaxis. Finally, 152 cases (from Group 1 or 2) received a 5 day course with the culprit antibiotic. This resulted in a rash in nine children (5.9 %) during the course. In both groups confounding illnesses, initial reaction, gender and co-existing allergies did not predict testing outcome.

**Conclusions**: In our cohort, the rate of allergy confirmed by skin testing was significantly higher than for those who underwent a direct challenge (17.6 vs. 3.7 %). The rate of reactivity to OPC was comparable for Group 1 and 2. These data further supports that performing direct supervised OPC with the culprit drug in children may be safe and potentially may avoid the need for resource intensive skin testing. Extended courses with the culprit drug should be considered, allowing confirmation of non-immediate reactions such as cutaneous reactions.

**Keywords**: Allergy; Antibiotic; Anaphylaxis

### P14 Drug hypersensitivity reactions in children and results of diagnostic evaluation

#### Neringa Buterleviciute, Odilija Rudzeviciene

##### Vilnius University Faculty of Medicine Centre of Children Pulmonology and Allergology, Vilnius, Lithuania

**Correspondence**: Neringa Buterleviciute

*Clinical and Translational Allergy* 2016, **6(Suppl 3)**:P14

**Background**: Patients or parents reported drug allergy in children is more common than the true drug allergy incidence. The aim of our study was to evaluate the clinical pattern of patient/parent reported drug hypersensitivity reactions and results of diagnostic evaluation.

**Materials and methods**: 15 children who were tested for drug allergy in 2015 were included in the study: eight boys (53.3 %) and seven girls (46.7 %), age range 1–15 years. We analysed causes, clinical pattern of reported drug hypersensitivity reactions and results of diagnostic evaluation.

**Results**: 26 drug hypersensitivity reactions were reported. Three drug hypersensitivity reactions were reported in four children, two reactions—in three children. The main suspected drugs were antibiotics 17 (65.4 %), NSAIDs—5 (19.2 %) cases, local anaesthetics—4 (15.4 %) cases. Amoxicillin was the most frequently suspected drug (six (23.1 %) cases). 8 (30.8 %) reactions appeared during 1 h. Skin symptoms were reported in 24 (92.3 %) cases: maculopapular rash—20 (76.9 %), angioedema—5 (19.2 %) cases. Respiratory and cardiovascular symptoms were reported in two cases (7.7 %). Drug provocation test was positive only for one child, who experienced angioedema after nimesulide and ibuprofen intake, and drug provocation test was positive to ibuprofen.

**Conclusions**: The most common suspected drugs were antibiotics, especially amoxicillin. Skin was the most frequently affected and maculopapular rash was the most common symptom. Drug provocation test was positive only for one patient.

**Keywords**: Drug hypersensitivity; Chidren; Clinical pattern; Diagnostics

### P15 Nonimmediate cutaneous drug reactions in children: are skin tests required?

#### Ana Sofia Moreira^1^, Carmo Abreu^2^, Eva Gomes^2^

##### ^1^Centro Hospitalar Vila Nova Gaia e Espinho, Vila Nova Gaia, Portugal; ^2^Centro Hospitalar do Porto, Porto, Portugal

**Correspondence**: Ana Sofia Moreira

*Clinical and Translational Allergy* 2016, **6(Suppl 3)**:P15

**Background**: Delayed urticaria and maculopapular eruptions associated with antibiotics are the most common reasons for referral to our pediatric drug allergy clinic. In this context, several studies point to the limited usefulness of skin tests, even to b-lactam antibiotics, with some authors advocating the use of drug provocation test without the need for other previous diagnostic procedures. Our aim was to describe the cases followed in our department in which this diagnostic approach was used (exclusive drug provocation test).

**Materials and methods**: Retrospective analysis of medical records from children referred to our drug allergy clinic due to suspected hypersensitivity to antibiotics, who reported nonimmediate cutaneous reactions (urticaria or maculopapular eruption) without signs of severity or systemic involvement. All children underwent provocation test with the suspected drug without conducting previous skin tests. The drug provocation test was extended to include the number of days of treatment reported at index reaction. Data was collected regarding gender of patients, age at the drug reaction, drug involved, symptoms reported on the reaction and results of the drug provocation test.

**Results**: We evaluated 213 children of which 50 % (n = 107) were male, with a median age of 3 years (6 months–17 years) at the time of reaction. In 97 % (n = 206) of the cases the involved antibiotics were b-lactam (amoxicillin-clavulanic acid in 92 patients, amoxicillin in 89 and cephalosporins in five cases). Only three children had positive drug provocations tests. In all cases the symptoms were similar to those previously reported and easily controlled with oral antihistamine.

**Conclusions**: Performing an exclusive drug provocation test in children with suspected hypersensitivity to antibiotics, who present with nonimmediate cutaneous reactions without signs of severity, proved to be a safe and effective approach.

**Keywords**: Skin tests; B-Lactams; Diagnosis

### P16 Pediatric patients with a history of penicillin allergy and a positive penicillin skin test may not be at an increased risk for multiple drug allergies

#### Sara May^1^, Thanai Pongdee^2^, Miguel Park^3^

##### ^1^University of Nebraska Medical Center, Omaha, USA; ^2^Mayo Clinic, Jacksonville, USA; ^3^Mayo Clinic, Rochester, USA

**Correspondence**: Miguel Park

*Clinical and Translational Allergy* 2016, **6(Suppl 3)**:P16

**Background**: Patients with a sulfonamide allergy or penicillins (PCN) may be at increased risk for reactions to other drugs. However, the studies were conducted in adults without PCN skin testing (PST) to confirm a PCN allergy. We conducted a study to determine if pediatric patients with a history of PCN allergy and a positive PST were at an increased risk for multiple drug allergies.

**Materials and methods**: Children (<18 years) with a history of PCN allergy were evaluated with PST and reviewed for basic demographic, PST results, and other medication allergies listed in the allergy module in the electronic medical record. A univariate logistic regression analysis was employed to calculate the odds ratio (OR) and the 95 % confidence interval (CI). *P* value of 0.05 or less was considered statistically significant. The Institutional Review Board (IRB) approved the study.

**Results**: 778 children underwent penicillin skin test. 703 (90.4 %) of 778 patients had a negative PST, 66 (8.5 %) were positive, and 9 (1.1 %) were equivocal. The overall mean ± standard deviation (SD) age of the study group was 5 ± 3.5 years. Three hundred and sixty-seven (47.1 %) were females. 703 children (90.4 %) had a negative PST, 66 patients (8.5 %) positive PST, and 9 (1.1 %) equivocal PST. 181 (23 %) of 778 patients reported a history of multiple drug allergies. Among the 181 patients reporting a history of multiple drug allergies, 81 (45 %) were female and 100 (55 %) were male. Males were 1.6 times (95 % CI 0.8–1.6, p = 0.5) more likely than females to report multiple drug allergies, although not statistically significant. 14 (21 %) of 66 patients with a positive PST reported multiple drug allergies compared to 167 (23 %) of 712 (p = 0.76) patients with a negative PST. Those patients with multiple drug allergies and a history of PCN allergy, cephalosporin [15 % (114 of 778)] was the most common medication listed in the allergy module. Other medication listed in the medication allergy modules were 10 % (79) macrolide antibiotics, 9 % (71) sulfonamides, 0.4 % (3) quinolones, and 1 % (9) nonsteroidal anti-inflammatory drugs (NSAIDS).

**Conclusions**: PCN allergic pediatric patients may not be at an increased risk for multiple drug allergies. Among patients with a history of PCN allergy, cephalosporin, macrolide antibiotics and sulfonamide antibiotics were the most common drug allergies listed in the medication allergy module.

**Keywords**: Penicillin allergy; Multiple drug allergies; Risk

### P17 Proved hypersensitivity to drugs according data of Vilnius University Hospital Santariskiu Klinikos

#### Linas Griguola^1^, Arturas Vinikovas^1^, Simona Kašinskaite^2^, Violeta Kvedariene^2^

##### ^1^Vilnius University, Faculty of Medicine, Vilnius, Lithuania; ^2^Center of Pulmonology and Allergology, Clinic of Infectious, Chest Diseases, Dermatology and Allergology, Vilnius University Hospital Santariskiu Klinikos, Vilnius, Lithuania

**Correspondence**: Linas Griguola

*Clinical and Translational Allergy* 2016, **6(Suppl 3)**:P17

**Background**: Adverse reactions to drugs are common problem, but true hypersensitivity to drug is rare.

**Aim of the study:** to investigate true hypersensitivity reactions among female and male with suspicion of drug allergy.

**Materials and methods**: 755 patients with suspicion of allergic reactions to drugs were addressed to consultations in Pulmonology and Allergology center by general practitioners. Patients were divided into groups by sex and suspected drugs: I—Antibiotics (AB), II—NSAID, III—Other. Each category was divided into subcategories: IA beta-lactam AB, IB Other AB; IIA ASA, IIB acetaminophen, IIC other NSAID; IIIA local anesthetics (LA), IIIB iodine containing contrasts (ICC), IIIC Other.

**Results**: 755 patients with 980 cases of suspected drug allergy were investigated: 618(81.85 %) females with average age 48.4(SD ± 15.2) and 137(18.15 %) males with average age 47.6(SD ± 18).

Female group had 821(83.8 %) adverse reactions to drugs. 199(24.2 %) reactions to I (AB): 151(75.9 %) were to beta-lactams, from all tested 36(16.9 %) were true hypersensitivity; 46(23.1 %) reactions were to other-AB, from all tested reactions 4(9.1 %) were proved. 2(1 %) cases were not tested. Accordingly: II (NSAID) had 306(37.3 %) reactions: to aspirin—56(18.3 %), 4(9.6 %) were proved; to acetaminophen—47(15.3 %), 12(40 %) were proved; IIC (Other-NSAID)—203 (66.3 %), 29(19.2 %) were proved. III (Other) had 316(38.5 %) reactions: IIIA (LA)—99(31.3 %), 1(1.5 %) was proved; IIIB (ICC)—20(6.3 %), 2(15.5 %) were proved; IIIC (Other)—197(24 %), proved—19(15.4 %).

Male group had 159(16.2 %) adverse reactions to drugs. I (AB) had 30(18.8 %) reactions: IA (beta-lactam)—25(83.3 %), from all tested reactions 7(21.2 %) were proved. Accordingly: IB (other-AB)—5(16.6 %), all negatives. II category had 77(48.4 %) reactions: to aspirin—17(22 %), 2(16.6 %) were proved; IIB (acetaminophen)—18(23.4 %), 2(11.8 %) were proved; IIC (other-NSAID)—42(54.5 %), DPTs proved 2(6.9 %). III category had 48(30.2 %) reactions: IIIA (LA)—16(33.3 %), 1(7.7 %) was proved; IIIB (ICC)—6(12.5, all negatives; IIIC (Other)—26(54.2 %), DPTs proved 4(22.2 %). NA—4(2.5 %).

**Conclusions**: Of all reactions true hypersensitivity were proved for: antibiotics—15.6 %, NSAIDs—20.2 %, other drugs—11.1 % in female group and 20 % for AB, 10.3 % for NSAIDs, 13.8 % for other drugs in male group.

**Keywords**: Drug; Hypersensitivity

### P18 Self-reported prevalence of drug hypersensitivity reactions among students in Celal Bayar University, Turkey

#### Ayse Aktas, Suheyla Rahman, Huseyin Elbi, Beyhan Cengiz Ozyurt

##### Celal Bayar University, Manisa, Turkey

**Correspondence**: Ayse Aktas

*Clinical and Translational Allergy* 2016, **6(Suppl 3)**:P18

**Background**: Drug hypersensitivity reactions (DHR) is defined “unwanted and harmful reactions occurring to drugs prescribed dose” by World Health Organization (WHO). DHR is an important health problem because of causing life-threatening condition, extending the length of stay in hospital and increasing the cost of treatment.

The aim of this study was to determine the prevalence of self-reported DHR among students at the university.

**Materials and methods**: A structured questionnaire was carried out to students of Celal Bayar University in Manisa, Turkey.

**Results**: 2086 students have participated in our study. 1217 (58.3 %) of them were women, 869 (41.7 %) were male. The mean prevalance of self-reported DHR was 5.3 % (111/2086). Drug allergy incidence of students (male/female) was 3.5 and 6.7 %, respectively (p < 0.001). The most common allergic reactions were rash 52.2 %, cardiovascular reactions 12.6 % and respiratory reactions 11.7 %. Aforementioned two systems involvement were 23.4 %. The most frequently involved drugs were antibiotics 52.2 % (n: 58) and analgesics 24.3 % (n: 27).

**Conclusions**: The diagnosis of drug allergy is based on a detailed history of the onset of symptoms/signs’ relationship between the appearance of symptoms and drug use. Misinterpretations just based on the DHR story can effect the individual treatment options. A definitive diagnosis can be easily reachable with a complete clinical history, standardized skin tests and drug provocation tests. Therefore we can recommend that doctors should be more informed about the managements of DHR due to self-reported DHR is highly prevalant just like shown in this study.

**Keywords**: Drug hypersensitivity; Prevalence

### P19 Severe drug hypersensitivity reactions in pediatric age

#### Ozlem Cavkaytar, Betul Karaatmaca, Pinar Gur Cetinkaya, Saliha Esenboga, Umit M. Sahiner, Bulent E. Sekerel, Ozge Soyer

##### Hacettepe University School of Medicine Department of Pediatric Allergy, Ankara, Turkey

**Correspondence**: Ozge Soyer

*Clinical and Translational Allergy* 2016, **6(Suppl 3)**:P19

**Background**: Epidemiologic data on drug induced anaphylaxis (DIA) in pediatric age is lacking. The aim of this study is to define the actual rate of drug induced anaphylactic reactions in childhood together with the severity and culprit drug patterns.

**Materials and methods**: Patients with a history of drug hypersensitivity reaction (DHR) referred between January 2012-December 2015 were included. After filling out an European Network for Drug Allergy (ENDA) questionnaire, initial skin tests and/or provocation tests were performed for the offending drug. The severity of anaphylactic reactions was determined as mild, moderate and severe according to EAACI guidelines on anaphylaxis in childhood.

**Results**: Among 627 children and adolescents referred due to a DHR, diagnostic work-up was completed in 532 patients. 103 (19.3 %) of them [54.4 % male, median age (interquartile range; 9.6 years (5.3–13.3)] had anaphylaxis in the history and diagnostic tests revealed that 75 (14.1 % of all evaluated patients, 72.8 % of all patients with a DIA) of them were actually hypersensitive to the offending drug. The culprit drugs responsible from actual DIA were antibiotics (36 %), NSAIDs (22.7 %), chemotherapeutics (20 %), biologicals (6.7), anesthetic agents (5.3 %), enzyme therapy (4 %) and others. Majority of the patients with actual DIA reported moderate (38.7 %) and severe (37.3 %) drug induced anaphylactic reactions. History of chronic disease (44 vs. 17.9 %, p = 0.014), concomitant intake of other drugs regularly (40 vs. 7.1 %, p = 0.001), cyanosis (23 vs. 3.6 %, p = 0.022) and hospitalization (56.2 vs. 17.9, p = 0.001) due to the suspected DIA and use of chemotherapeutics as the culprit drug (20 vs. 0 %, p = 0.01) were more frequent, whereas use of antibiotics was less frequent (71.4 vs. 36 %, p = 0.001) in patients with actual DIA compared to nonhypersensitive patients. Atopic disease, atopy or family history of atopy or drug hypersensitivity did not have an impact on actual DIA. It is important to note that actual DIA was more frequent in children younger than 10 years of age compared to older adolescents (81.5 vs. 63.3 %, p = 0.038).

**Conclusions**: During childhood, any history of DIA reaction may not be an actual DHR, however young age, existence of chronic disease and use of chemotherapeutics might point out the actual drug induced anaphylaxis in children and adolescents.

**Keywords**: Drug hypersensitivity; Anaphylaxis; Child; Pediatric

## Poster Walk 3: Desensitisation (P20–P28)

### P20 A protocol for desensitisation to valaciclovir

#### Celia Zubrinich, Bianca Tong, Mittal Patel, Michelle Giles, Robyn O’Hehir, Robert Puy

##### Alfred Health, Melbourne, Australia

**Correspondence**: Celia Zubrinich

*Clinical and Translational Allergy* 2016, **6(Suppl 3)**:P20

**Background**: We report a protocol for desensitisation to valaciclovir.

**Report**: A woman in her 40 s developed generalised urticaria on the second or third day of her initial course of acyclovir (administered five times daily) for newly diagnosed genital herpes. The urticaria continued to cause distress despite substitution to valaciclovir for a further few days. Antiviral medication was ceased. The rash resolved over 2 weeks with the use of anti-histamines. For more than a decade since then, she has experienced frequent and prolonged confirmed herpetic recurrences, more often present than not. The attacks had a marked viral prodrome and significantly affected her lifestyle.

Recurrent genital herpes may be treated with suppressive antiviral therapy if frequent. Supervised direct challenge to valaciclovir was an option but desensitisation was favoured because it required a single clinic visit and, of relevance to the patient, likely entailed less risk. We identified reports of desensitisation to acyclovir but not valaciclovir. The final treatment dose of acyclovir (usually 400 mg) differs from that of valaciclovir (500 mg). Acyclovir therapy requires multiple doses per day, whereas valaciclovir requires only a single daily dose. We developed and present a 14-dose oral desensitisation protocol that was administered over 3.5 h (Fig. 1). The first 10 doses were valaciclovir in suspension and the final four doses were prepared from a 500 mg tablet. The patient tolerated the procedure with no ill effect. There has not been a single herpetic recurrence in the 12 months since the treatment commenced.

**How this report contributes to current knowledge**: Desensitisation to valaciclovir may be performed where suppressive therapy for herpes is indicated.

**Consent**: Written informed consent was obtained from the patient for publication of this abstract and any accompanying images.

### P21 A rare case of desensitization to modafinil

#### Josefina Cernadas, Luís Amaral, Fabrícia Carolino

##### Serviço de Imunoalergologia, Centro Hospitalar de São João E.P.E., Porto, Portugal

**Correspondence**: Luís Amaral

*Clinical and Translational Allergy* 2016, **6(Suppl 3)**:P21

**Background**: Modafinil is a 2-benzhydrylsulfinylethanamide molecule, belonging to a class of medications called wakefulness promoting agents used to treat excessive sleepiness caused by narcolepsy (a condition that causes excessive daytime sleepiness) or shift work sleep disorder. Another use is to prevent excessive sleepiness caused by obstructive sleep apnea/hypopnea syndrome. It acts as a blocker of a type of molecule called the dopamine transporter. Rare occurrences including severe cutaneous adverse reactions, as erythema multiforme, SJS, TEN and DRESS, affecting adults and children and probably allergic, have been reported. Rare cases of angioedema and multi-organ hypersensitivity reactions have also been described.

**Report**: The authors describe a case of a 27 year-old male with the diagnosis of narcolepsy medicated with modafinil 100 mg in an increasing dosage. Four days after beginning the medication, he complained of generalized pruritus, maculopapular exanthema and angioedema of upper and lower limbs. No analytical changes were found in the acute phase. He was treated with intravenous corticosteroid and antihistamines and, 4 days after complete recovery, the drug was re-introduced with reproducible signs and symptoms arising 12 h after the intake of 100 mg of modafinil. Because this was the only therapeutic option for the patient he was referred to our Allergy Department for desensitization. A suspension of 1 mg/ml (100 mg/100 ml) was prepared and a “tailor made” desensitization protocol was started with an initial dose of 1 mg, followed by doubling doses, taken with 1 h intervals. The cumulative dose reached on the first day was 15 mg, 85 mg on the second and 100 mg on the third and fourth days. For safety reasons, 100 mg/day were taken in the first week followed by an increase, to the intended doses (Neurology prescription) in the following weeks: 100 + 100 mg on the second week, 100 + 200 mg on the third week and 200 + 200 mg on the fourth and on, as chronic treatment. After we have achieved tolerance to a dose of 100 mg, the slowly increase to the targeted dose of 200 mg twice a day was well tolerated. The patient is still on the same therapeutic scheme with no further reactions.

**How this report contributes to current knowledge**: As far as we know this is the first case describing a desensitization protocol to modafinil.

**Consent**: Written informed consent was obtained from the patient for publication of this abstract and any accompanying images.

### P22 A sixteen-day desensitization protocol in delayed type hypersensitivity reactions to oral drugs

#### Semra Demir, Asli Gelincik, Muge Olgac, Raif Caskun, Derya Unal, Bahauddin Colakoglu, Suna Buyukozturk

##### Istanbul University, Istanbul Faculty of Medicine, Istanbul, Turkey

**Correspondence**: Semra Demir

*Clinical and Translational Allergy* 2016, **6(Suppl 3)**:P22

**Background**: Although desensitization in immediate type hypersensitivity reactions due to chemotherapeutics is well described and standardized for many drugs, common protocols in delayed type hypersensitivity reactions (DHR) are not standardized. Our aim is to evaluate the usefulness of the 16-day desensitization protocol which we had recently described for capasitabine in DHRs caused by various oral chemotherapeutic drugs.

**Materials and methods**: We applied our slow desensitization protocol which started with 1/100 of the target dose and was completed in 16 days with slow incremental doses in patients who had experienced DHR due to various oral chemotherapeutic drugs.

**Results**: Four patients (two female, two male) who were referred to our clinic for desensitization were included. The mean age was 55 ± 22.25 years. The culprit drugs were pazopanib, nilotinib and lenalidomide. The mean reaction time after the initiation of the drug was 7.5 ± 2.08 days and the reaction types were maculopapular rush (MPR) in two patients and generalized morbiliform rushes (GMR) in two patients. Two patients who had experienced MPR caused by lenalidomide completed the protocols without any reactions. However, the other two patients developed pruritus and rush on the 9th and the 15th days of the desensitization protocols. After the reactions were treated with methylprednisolone, the doses were decreased to the last tolerated doses and gradually increased afterwards. One of them completed the desensitization in 28 days. However, the second patient could tolerate almost the ¾ of the targeted dose.

**Conclusions**: This sixteen-day protocol seems to be a useful guide for the desensitization in delayed type hypersensitivity reactions especially in MPR in oral chemotherapeutics. However, the protocol can be modified in some patients.

**Keywords**: Delayed type hypersensitivity reactions; Desensitization

### P23 Desensitization to intravenous etoposide using a 12 and a 13-step protocol. Two cases report

#### Olga Vega Matute^1^, Amalia Bernad^1^, Gabriel Gastaminza^1^, Roselle Madamba^1^, Carlos Lacasa^2^, M. J. Goikoetxea^1^, Carmen D’Amelio^1^, Jose Rifón^3^, Nicolas Martínez^3^, Marta Ferrer^1^

##### ^1^Departament of Allergy and Immunology Clínica Universidad de Navarra, Pamplona, Spain; ^2^Department of Pharmacy Clínica Universidad de Navarra, Pamplona, Spain; ^3^Departament of Hematology Clínica Universidad de Navarra, Pamplona, Spain

**Correspondence**: Amalia Bernad

*Clinical and Translational Allergy* 2016, **6(Suppl 3)**:P23

**Background**: Etoposide is a chemotherapy agent used to treat malignant conditions. Because of the repeated use of this drug in within time intervals for cancer treatment, there is a high chance as with any chemotherapeutic agent to induce hypersensitivity reactions (HSR). It is reported to induce HSR in 6 % of the patients.

**Report**: We present two cases. First is a 7 year old female patient with acute myeloid leukemia (AML). She had previously received two courses of treatment with etoposide. In the second course, with the first administration she had a reaction consisted with facial erythema and severe dyspnea. When evaluated in our hospital, she needed treatment with etoposide for autologous stem-cell transplantation. The second patient was an 8 year old male with refractory acute lymphoid leukemia (ALL-B) that had received six doses of treatment with etoposide. In the last dose, he reported pharyngeal obstruction and chest erythema. When evaluated in our hospital, he needed salvage chemotherapy containing etoposide followed by allogenic stem-cell transplantation.

We performed skin prick test with Etoposide, using commercially available drug solution for intravenous use. In both patients the test was negative.

Because Etoposide was the most suitable chemotherapeutic option for both patients, we decided to administer the drug using a desensitization protocol. Castells (2) developed a protocol for desensitization to drugs with a 12 steps infusion of the target dose. In the first case we had to add one step to this protocol because of the dose needed. She received premedication treatment and 400 mg of Etoposide in a 13 steps protocol, twice in consecutive days. She had no HSR, the transplantation went well and she is now in complete remission. In the second patient we followed the 12 step protocol with premedication treatment and the patient was able to receive the target dose (150 mg). We did three desensitization protocols with the total dose, in three consecutive days. He had no HSR and he continues in follow up in our hospital.

**How this report contributes to current knowledge**: Patients with HSR to etoposide, in which this drug is the most adequate chemotherapeutic option for such a severe disease, the desensitization protocol is a treatment that proved to be effective in order to administer the treatment.

**Consent**: Written informed consent was obtained from the patient for publication of this abstract and any accompanying images.

### P24 Drug desensitisation in oncology: the experience of an immunoallergology department for 5 years

#### Carmelita Ribeiro^1^, Emília Faria^1^, Cristina Frutuoso^2^, Anabela Barros^2^, Rosário Lebre^2^, Alice Pego^2^, Ana Todo Bom^1^

##### ^1^Allergy and Clinical Immunology Department, Coimbra University Hospital Center, Coimbra, Portugal; ^2^Oncology Department, Coimbra University Hospital Center, Coimbra, Portugal

**Correspondence**: Carmelita Ribeiro

*Clinical and Translational Allergy* 2016, **6(Suppl 3)**:P24

**Background**: Any cancer drug can potentially trigger a hypersensitivity reaction (HSR), particularly chemotherapy agents and monoclonal antibodies. The more frequent drugs inducing HSR are the platins (IgE-mediated reactions) and taxanes (non-immunological reactions). Desensitisation protocols must be considered when there is no valid and effective alternative treatment. This is especially relevant for cancer patients who are thus able to continue their first line treatment. The aim was to describe the experience of an Immunoallergology Department with desensitization to chemotherapy agents.

**Materials and methods**: Retrospective review of charts of oncology patients desensitized in the Immunoallergology Department of CHUC in the last 5 years.

**Results**: There were a total of 72 desensitisation procedures corresponding to 15 patients treated (11 female) during the period in question, with a mean age of 56 years (range 28–73 years). The patients had ovarian cancer (eight patients), lung cancer (three patients), colon cancer (2), rectal cancer (1) and breast cancer (1). The HSR were all with moderate to severe immediate reactions: rash, urticaria, laryngeal stridor, bronchospasm, syncope and anaphylaxis (six patients). Eleven patients were desensitized to platins (carboplatin n = 6, cisplatin = 3, oxaliplatin n = 2), three to taxanes (docetaxel n = 2, nabpaclitaxel n = 1) and one to monoclonal antibodies (panitumumab). In the total of 72 desensitisation procedures, the range of desensitisation procedures for patient was 17 (maximum) to 2 (minimum) treatmens with carboplatin. There were five desensitisation procedures in 2011, 12 in 2012, 19 in 2013, 18 in 2014 and also in 2015. In the 72 desensitisation did not have any reaction in 59 procedures (82 %) and in the others, the reactions were milder than the initial HSR (only one patient had anaphylaxis in the third desensitization with cisplatin). All patients received their daily programmed dose. Five patients discontinued the desensitization programme (three patients due to progression of the oncological disease, one patient due to neurological toxicity and another for anaphylaxis during de desensitization).

**Conclusions**: In the majority of patients, desensitization procedures allowed safe reBackground of chemotherapy agents in Immunoallergology centers with experience. This approach must be considered by Oncologic doctors in the treatment of specific oncologic patients with previous history of HSR to these drugs.

**Keywords**: Drug desensitisation; Oncology; Chemotherapy agents; Platins; Taxanes

### P25 Filgrastim anaphylaxis: a successful desensitization protocol

#### Luis Amaral, Josefina Cernadas

##### Serviço de Imunoalergologia, Centro Hospitalar de São João E.P.E., Porto, Portugal

**Correspondence**: Luis Amaral

*Clinical and Translational Allergy* 2016, **6(Suppl 3)**:P25

**Background**: Filgrastim is a recombinant human granulocyte colony-stimulating factor (G-CSF) and is used to prevent or treat neutropenia that is generally associated with chemotherapy. Anaphylatic reactions to filgrastim are rarely reported and there is only one published successful desensitization protocol in a patient with immediate hypersensitivity to filgrastim.

**Report**: We describe a case of a 47-year-old woman with ductal breast carcinoma receiving chemotherapy with doxorubicin, cyclophosphamide and docetaxel. She developed chemotherapy induced neutropenia for which she was started on SC filgrastim [300 mcg] days after the chemotherapy cycle, given once a day, for four consecutive days. Ten minutes after the 4th dose of filgrastim, she developed facial erythema, labial angioedema, dyspnea and colicky abdominal pain. These symptoms quickly reversed spontaneously in 48 h. Subsequently, filgrastim was withdrawn and anaphylaxis work-up was done.

A 5-step desensitization protocol was performed using sequential doses intravenously: 15, 30, 60, 80, 115 to a cumulative dose of 300 mcg diluted in 20 ml of saline over approximately 3 h. In the following day she received 300 mcg in two steps with success and in 3rd day in one without any reaction.

**How this report contributes to current knowledge**: In summary, we present a successful desensitization protocol for filgrastim in a patient with a previous life-threatening immediate hypersensitivity reaction.

**Consent**: Written informed consent was obtained from the patient for publication of this abstract and any accompanying images.

### P26 Galsulfase hypersensitivity and desensitization of a mucopolysaccharidosis VI patient

#### Luis Felipe Ensina, Carolina Aranda, Ines Camelo Nunes, Ana Maria Martins, Dirceu Solé

##### Federal University of São Paulo, São Paulo, Brazil

**Correspondence**: Luis Felipe Ensina

*Clinical and Translational Allergy* 2016, **6(Suppl 3)**:P26

**Background**: Mucopolysaccharidosis VI or Maroteaux-Lamy Syndrome (MPS VI) is a lysosomal storage disorder caused by deficiency of arylsulfatase B. The enzyme replacement therapy (ERT) with galsulfase is so far the only specific treatment for MPS VI. Infusion-related reactions (IRR) to ERT can occur and can be severe, precluding further ERT. However, the interruption of ERT can accelerate the disease progression and precipitate earlier death. The aim of this study is to report IRR to galsulfase in a MPS VI Brazilian patient, and his treatment reestablishiment after desensitization.

**Materials and methods**: a 7 year-old male started presenting symptoms such as dyspnea and itch during his 44th infusion. No improvement was observed with premedication (corticosteroids and antihistamines) in the folowing infusion. ERT were stopped for 4 weeks and the patient underwent skin tests (prick and intradermal). Skin prick test was performed using the straight concentration of galsulfase and Intradermal tests with progressive dilutions. Skin tests were also performed in 10 healthy subjects and in a MPS VI patient without IRR, to rule out a skin irritant effect, since skin tests with galsulfase have not been standardized.

**Results**: Patient presented a positive skin prick test, suggesting an IgE-mediated reaction. Therefore, a rapid desensitization protocol was generated. Three solutions (each 250 ml 0.9 % sodium chloride—1:100, 1:10 and 1:1 respectively) were delivered in 12 consecutive steps at an increasing infusion rate. Pre-medications were used to block different allergy pathways. This protocol has succeeded for 6 months, when during the 22th infusion he started to present wheals in the beginning of the third bag. A new protocol was developed (four bags—16 steps), but the patient still had symptoms. A 75 mg omalizumab (Omab) monthly was introduced. Ten days after the Omab, a new infusion using the same desensitization protocol was performed with no reaction.

**Conclusions**: To our knowledge, we are the first group to use an adapted 12-step desensitization protocol to galsufase with omalizumab. The protocol has shown to be safe and effective for the patient receiving the enzyme in the recommended dose. Management of these reactions with desensitization can provide first line therapy and permit continued ERT.

**Keywords**: Omalizumab; Desensitization

**Consent**: Written informed consent was obtained from the patient for publication of this abstract and any accompanying images.

### P27 Rapid drug desensitization with biologicals: one-center experience with four biologicals

#### Sevim Bavbek, Resat Kendirlinan, Pamir Çerçi, Seda Tutluer, Sadan Soyyigit, Zeynep Çelebi Sözener, Ömür Aydin, Reyhan Gümüsburun

##### Ankara University, Ankara, Turkey

**Correspondence**: Sevim Bavbek

*Clinical and Translational Allergy* 2016, **6(Suppl 3)**:P27

**Background**: Rapid drug desensitization (RDD) induces a temporary tolerance to biological drugs inducing hypersensitivity reactions (HSRs) but data are limited regarding use of this outside of the USA. Therefore in this study we aimed to report our data with RDD to rituximab, infliximab, cetuximab and trastuzumab.

**Materials and methods**: The study was conducted as a retrospective chart review of patients with symptoms of HSRs to biological agents to which RDD have been performed between January 2012 and January 2016. HSRs were classified as Grade I, II and III based on their severity. Skin prick/intradermal tests were performed with implicated biologicals. The 12-step RDD protocol developed at Brigham and Women Hospital was used

**Results**: Study group consisted of 11 female and four men (mean age: 54, 21 ± 11 years). Majority of the study subjects (66.6 %) were using the biological agents for the treatment of malign diseases. Twelve subjects had experienced HSR to rituximab, three had HSR to cetuximab, infliximab and trastuzumab respectively. HSR to cetuximab, infliximab and trastuzumab occurred during first infusion and all were Grade III, 11 of 12 subjects with rituximab hypersensitivity had reaction during first infusion, nine had Grade II and three had Grade III. Of the 15 patients, 86.6 % had respiratory symptoms, 66.6 % had cardiovascular, 60 % had cutaneous symptoms, and 40 % had gastrointestinal symptoms. Skin tests with rituximab were done on eight patients, only three resulted in positive in 1/100 dilutions of intradermal tests, and skin tests were negative on either prick or intradermal tests with other biologicals. Of 88 RDD, 81 desensitizations with rituximab, five desensitizations with cetuximab, and one desensitization with infliximab and one desensitization with trastuzumab were done. There were nine breakthrough reactions (10.1 %) in seven subjects, which were all associated with rituximab and less severe than initial reactions. Of breakthrough reactions, 55.5 % were cutaneous, followed by cardiovascular (33 %), respiratory (22.2 %), and gastrointestinal (11.1 %) symptoms. Reactions mainly occurred at step 12, and all were successfully under-controlled and except single desensitization, all desensitization procedures were completed with full target dose.

**Conclusions**: We found RDD is safe and effective, to our knowledge, in the largest series of biological including Rituximab in our country.

**Keywords**: Biologicals; Rituximab; Infliximab; Rapid drug desensitization

### P28 Successful desensitization to a high dose of methotrexate in a delayed type hypersensitivity reaction

#### Josefina Cernadas^1^, Leonor Carneiro-Leão^1^, Fabrícia Carolino^1^, Marta Almeida^2^

##### ^1^Serviço de Imunoalergologia, Centro Hospitalar de São João, Porto, Portugal; ^2^Serviço de Pediatria, Instituto Português de Oncologia do Porto Francisco Gentil, Porto, Portugal

**Correspondence**: Leonor Carneiro-Leão

*Clinical and Translational Allergy* 2016, **6(Suppl 3)**:P28

**Background**: Delayed type hypersensitivity reactions are mediated by cells rather than by antibodies. They can be life-threatening and classically constitute a contraindication to desensitization (DZT). However, in selected cases of uncomplicated exanthemas, when the culprit drug is irreplaceable or more effective, this option might be considered. Methotrexate (MTX) is a folic acid antagonist with a major role in the treatment of B cell acute lymphoblastic leukemia (ALL). The authors describe a successful DZT to a high dose of IV MTX associated to inthrathecal administration, following a delayed-type hypersensitivity reaction.

**Report**: We report a case of a 12 year old boy under B cell ALL treatment. He was scheduled to receive four cycles of MTX 8000 mg IV perfusion over 24 h (10 % on the 1st hour, 90 % over the remaining 23 h, with a constant concentration), followed by inthrathecal administration of MTX 12 mg, cytarabine 30 mg and prednisolone 10 mg. He complained of an intensely pruritic morbilliform exanthema in his abdomen arising 72 h after completion of the first treatment. The rash resolved with anti histamines and oral and topic corticosteroids, over a period of 10 days with severe residual hyperpigmentation. No renal or liver function analytical changes were found during this episode. Because no alternative treatment was available for his specific type of leukemia, a decision to perform DZT was made.

The patient (60 kg) was pretreated with montelukaste 10 mg, starting 2 days before chemotherapy (QT); prednisolone 60 mg was started the day before QT and maintained over 4 days in decreasing doses. He was admitted and a 14 step DZT protocol to MTX was performed. The doses administered were doubled in each step until the 14th step, with a cumulative dose of 1671 mg of MTX infused over a period of 4.5 h. The remaining dose, until the target dose of 8000 mg was infused at a small rate during 24.5 h. This procedure was followed by triple inthrathecal treatment, including MTX. The patient was able to reach the target dose without any reaction. He has now completed 3 of the 4 scheduled cycles.

**How this report contributes to current knowledge**: To our knowledge this is the first description of a successful DZT to such a high dose of MTX in a delayed type reaction. The IV DZT protocol also made possible the administration of inthrathecal MTX. Our findings support that DZT protocols might be suitable in selected cases, allowing these patients to safely continue with first line therapy.

**Consent**: Written informed consent was obtained from the patient for publication of this abstract and any accompanying images.

## Poster Walk 4: SJS (P29–P38)

### P29 Assessment of impact of infection on drug-induced severe cutaneous adverse reactions and rhabdomyolysis using the Japanese adverse drug event report database

#### Kimie Sai^1^, Takuya Imatoh^1^, Ryosuke Nakamura^1^, Chisato Fukazawa^2^, Yasushi Hinomura^2^, Yoshiro Saito^1^

##### ^1^National Institute of Health Sciences, Tokyo, Japan; ^2^Japan Pharmaceutical Information Center, Tokyo, Japan

**Correspondence**: Kimie Sai

*Clinical and Translational Allergy* 2016, **6(Suppl 3)**:P29

**Background**: Involvement of immune-mediated mechanism has been demonstrated in a certain type of serious adverse drug reactions (ADRs), such as severe cutaneous adverse reactions (SCAR) or rhabdomyolysis (RM), and thus, a possible potentiation of these ADRs by infection via activating immune reactions is postulated. In this study, to assess a role of infection in occurrence and severity of SCAR and RM in Japanese cases, we analyzed a relation of infection to reporting rates of these ADRs and severe outcomes or periods to onsets using the Japanese Adverse Drug Event Report database (JADER).

**Materials and methods**: A total of 302,641 reports regarding primary suspected drugs from 2009 to 2013 in JADER were used in our analysis. Of these, a total of 3167 SCAR reports (Stevens–Johnson syndrome, toxic epidermal necrolysis (TEN), or mucocutaneous ocular syndrome) and 1847 RM reports were included. Infection-Positive (Infect-P) was defined if the individual case report included data on anti-infectious drugs or infection-related diseases, either as a primary suspected drug (PS) or concomitant drugs/diseases (CO). Rates of infections for SCAR or RM among total reports, and ratios of severe phenotype (TEN for SCAR) and severe outcomes (no recovery, permanent damage and death) and period to onset of SCAR or RM were compared between Infect-P and Infection-Negative (Infect-N).

**Results**: The reporting ratios for Infect–P (vs. Infect-N) were significantly higher in SCAR (adjusted odds ratio [95 % CI]: 1.8 [1.7–2.0] for PS and 2.0 [1.8–2.2] for CO, respectively) and lower in RM (0.6 [0.5–0.7] for PS and 0.8 [0.7–0.9] for CO). The rate of TEN in SCAR was significantly higher in Infect-P (vs. Infect-N) (1.3[1.1–1.6] for PS and 2.2 [1.8–2.7] for CO). Higher rates of severe outcomes by Infect-P (vs. Infect-N) were observed for SCAR (1.6 [1.2–2.1] for PS and 1.9[1.4–2.5] for CO) and RM (1.3 [0.8–2.2] for PS and 1.7 [1.0–2.3] for CO). The mean period to onset of SCAR was shorter for PS (25 days) but marginal for CO (40 days) in Infect-P comparing with Infect-N (36 days), and earlier onset of RM was evident in Infect-P (47 days for PS and 191 days for CO) comparing with Infect-N (250 days).

**Conclusions**: This study indicated a substantial association between infection and occurrence/severity in SCAR as well as RM, showing much larger impacts on SCAR.

**Keywords**: Severe cutaneous adverse reactions; Rhabdomyolysis; Infection; Database

### P30 Characterization of erythema multiforme and severe cutaneous adverse reactions hospitalizations

#### Bernardo Sousa-Pinto, Cláudia Correia, Lídia Gomes, Sara Gil-Mata, Luís Araújo, Luís Delgado

##### Immunology Laboratory - Basic and Clinical Immunology, Faculty of Medicine, Porto, Portugal

**Correspondence**: Bernardo Sousa-Pinto

*Clinical and Translational Allergy* 2016, **6(Suppl 3)**:P30

**Background**: Erythema multiforme (EM) and severe cutaneous adverse reactions (SCARs) are immunologically-mediated delayed hypersensitivity reactions triggered after exposition to external agents such as drugs. We aim to characterize hospitalizations occurring in Portuguese public hospitals with associated diagnosis of drug-related EM and SCARs concerning patients’ demographic and clinical characteristics, in-hospital mortality and most frequently associated drug classes.

**Materials and methods**: We used a database containing all hospitalizations occurred in mainland Portugal public hospitals and analyzed all episodes with associated diagnosis of drug-related EM or SCARs—Stevens–Johnson syndrome (SJS), toxic epidermal necrolysis (TEN) and SJS/TEN overlap syndrome. These hospitalizations were compared concerning patients’ demographic and clinical characteristics (gender, age and comorbidities), length of stay, and in-hospital mortality. We also evaluated the drug classes most frequently associated with these diagnoses, estimating the number of episodes per million drug packages sold. A logistic regression was performed to evaluate for factors significantly associated with in-hospital mortality among SCARs.

**Results**: Between 2009 and 2014, there were 122 hospitalizations with associated diagnosis of EM and 132 with associated diagnosis of SCARs. Most EM (65 %) and SCARs (55 %) episodes occurred in women. The median age was 63 years old for both EM and SCARs. The frequency of HIV co-infection was higher among SCARs episodes (9 vs. 2 %; *p* = 0.009). In-hospital mortality varied between 7 % (EM) and 44 % (TEN). Advanced age (OR 1.06; 95 % CI 1.02–1.45), heart failure (OR 4.93; 95 % CI 1.01–23.99), liver disease (OR 7.39; 95 % CI 1.07–51.02) and TEN diagnosis (OR 14.66; 95 % CI 1.73–124.31) were all significantly associated with higher risk of in-hospital mortality among SCARs episodes. The highest numbers of EM and SCARs episodes per million drug packages sold concerned antiviral (1.5 and 8.7, respectively) and uric acid metabolism drugs (2.4 and 5.0, respectively).

**Conclusions**: SCARs are associated with higher mortality than EM. Advanced age, heart failure, liver disease and TEN diagnoses are associated with higher in-hospital mortality among SCARs episodes. Antiviral and uric acid metabolism drugs are the drug classes most frequently associated with SCARs hospitalizations.

**Keywords**: Erythema multiforme; Severe cutaneous adverse reactions; Epidemiology; Stevens–Johnson Syndrome; Toxic epidermal necrolysis

### P31 Effects of infection on incidence/severity of SJS/TEN and myopathy in Japanese cases analyzed by voluntary case reports

#### Ryosuke Nakamura^1^, Kimie Sai^1^, Takuya Imatoh^1^, Yoshimi Okamoto-Uchida^1^, Koji Kajinami^2^, Kayoko Matsunaga^3^, Michiko Aihara^4^, Yoshiro Saito^1^

##### ^1^National Institute of Health Sciences, Tokyo, Japan; ^2^Kanazawa Medical University, Ishikawa, Japan; ^3^Fujita Health University, Aichi, Japan; ^4^Yokohama City University, Kanagawa, Japan

**Correspondence**: Ryosuke Nakamura

*Clinical and Translational Allergy* 2016, **6(Suppl 3)**:P31

**Background**: Immunological mechanisms are supposed to be involved in the pathogenesis of certain types of serious adverse reactions (ADRs), such as severe cutaneous adverse reactions or rhabdomyolysis. Therefore, infection which activates immune system may affect incidence and severity of ADRs. In this study, we analyzed an association of concomitant infectious diseases with incidence/severity of Stevens–Johnson syndrome (SJS)/toxic epidermal necrolysis (TEN) and myopathy, based on Japanese voluntary case reports.

**Materials and methods**: Patients who experienced SJS/TEN (259 cases) or myopathy (a total of 129 cases) were recruited through a nationwide blood sampling network in Japan operated by the National Institute of Health Sciences under a cooperation of the Ministry of Health, Labour and Welfare, Pharmaceuticals and Medical Devices Agency, and Federation of Pharmaceutical Manufacturers’ Association of Japan. Written informed consent was obtained from all recruited patients. Using the case report data, we analyzed rates for concomitant infectious disease (tuberculosis, hepatitis, AIDS, influenza infection, and herpes simplex infection, etc.), and compared the severity (severe phenotypes or outcomes) and the mean period to onset of the ADRs between patients with and without infectios diseases.

**Results**: For SJS/TEN cases, the rate of infectious diseases was 51 %, of which 31 % was the cases caused by a primary suspected drug. The rates of severe phenotype TEN, ocular involvement and permanent damage were significantly higher (p < 0.05) and the mean period of onset of SJS/TEN was significantly shorter in the infectious patients’ group compared with non-infectious group. The rate of infectious diseases in myopathy was 23 %, of which 12 % was the cases caused by a primary suspected drug. The rates of severe phenotype and permanent damage were higher and the mean period of onset of myopathy was shorter in the myopathy patients with infectious disease than those without infection, although these differences were not statistically significant.

**Conclusions**: This study indicated that the incidence and the severity of SJS/TEN and myopathy are associated with the concomitant infectious disease, especially in the cases of SJS/TEN, which might reflect a greater contribution of immune system as a mechanism of occurrence.

**Keywords**: SJS; TEN; Myopathy; Infection; Adverse reaction

### P32 Efficacy of tumor necrosis factor—a antagonists in Stevens–Johnson syndrome and toxic epidermal necrolysis: a randomized controlled trial and immunosuppressive effects evaluation

#### Chuang-Wei Wang^1^, Shih-Chi Su^1^, Shuen-Iu Hung^2^, Hsin-Chun Ho^1^, Chih-Hsun Yang^1^, Wen-Hung Chung^1^

##### ^1^Department of Dermatology, Drug Hypersensitivity Clinical and Research Center, Chang Gung Memorial Hospital, Linkou And Keelung, Taiwan; ^2^Department and Institute of Pharmacology, School of Medicine, Infection and Immunity Research Center, National Yang-Ming University, Linkou, Taiwan

**Correspondence**: Chuang-Wei Wang

*Clinical and Translational Allergy* 2016, **6(Suppl 3)**:P32

**Background**: Stevens–Johnson syndrome (SJS) and toxic epidermal necrolysis (TEN) are rare, but life-threatening adverse drug reactions. While the clinical manifestations of SJS/TEN are well-defined, the optimal treatment for these diseases remains unavailable. We aim to evaluate the efficacy and safety of etanercept (anti-TNF-α agent) in patients with SJS/TEN.

**Materials and methods**: We enrolled 98 patients with SJS/TEN to perform an open, prospective, double-blind and randomized comparison trial of etanercept versus corticosteroid. We further investigated the immunologic effects of etanercept in SJS/TEN patients.

**Fig. 2:**
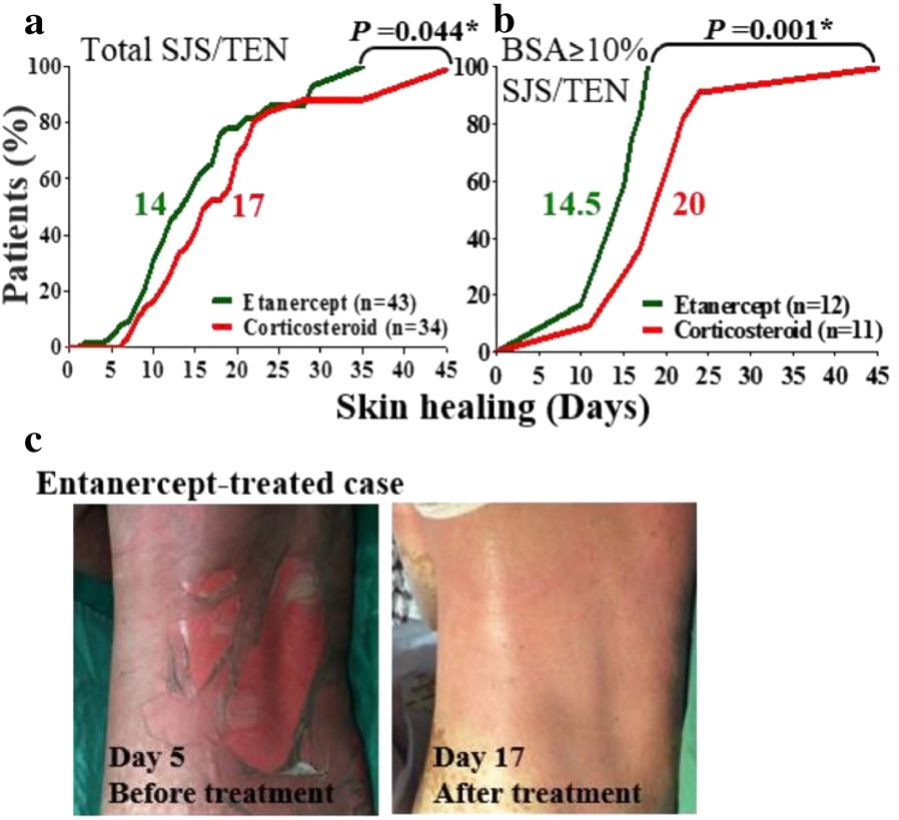
The clinical improvement in SJS/TEN patients after etanercept treatment

**Results**: This study demonstrated that etanercept had clinical improvements in patients with SJS/TEN. Etanercept revealed to be more decreased the SCORTEN-based predicted mortality rate (predicted rate = 17.47 %; observed rate = 8.33 %) than in corticosteroid use (predicted rate = 20.13 %; observed rate = 15.91 %). Compared with corticosteroid, etanercept also showed a shorter skin healing time (The median times are 14 days for etanercept and 17 days for corticosteroid; *P* < 0.05*) and less side effect for gastrointestinal hemorrhage (0 % after etanercept use and 11.8 % after corticosteroid use; *P* < 0.05*) in total SJS/TEN patients. For mechanism study, etanercept significantly decreased the granulysin and TNF-α expression in blister fluids and plasma (In blister fluids: granulysin = 62.5 % and TNF-α = 50.0 % decrease after 2 days treatment; in plasma: granulysin = 54.7 % and TNF-α = 69.2 % decrease after 15 days treatment, all *P* < 0.05*) and increased Treg cells population (twofold percentage increase after 15 days treatment, *P* < 0.01**) (Fig. 2).

**Conclusions**: This study indicates that etanercept (anti-TNF-α agent) not only has immunosuppressive effects but also serves as an alternative medicine for SJS/TEN treatment.

**Consent**: Written informed consent was obtained from the patient for publication of this abstract and any accompanying images.

### P33 Evolution of drug causality in Stevens–Johnson syndrome and toxic epidermal necrolysis in Europe: analysis of 10 years RegiSCAR-Study

#### Maren Paulmann^1^, Ariane Dunant^2^, Maja Mockenhaupt^1^, Peggy Sekula^3^, Martin Schumacher^3^, Sylvia Kardaun^4^, Luigi Naldi^5^, Teresa Bellón^6^, Daniel Creamer^7^, Cynthia Haddad^8^, Bruno Sassolas^9^, Bénédicte Lebrun-Vignes^10^, Laurence Valeyrie-Allanore^8^, Jean-Claude Roujeau^8^

##### ^1^Dokumentationszentrum schwerer Hautreaktionen, University Medical Center, Freiburg, Germany; ^2^Department of Biostatistics and Epidemiology Unit, Institut Gustave-Roussy, Villejuif, France; ^3^Institute of Medical Biometry and Medical Informatics, University Medical Center, Freiburg, Germany; ^4^Reference Center for Cutaneous Adverse Reactions, University Medical Center, Groningen, The Netherlands; ^5^Department of Dermatology, Papa Giovanni XXIII Hospital, Bergamo, Italy; ^6^Institute for Health Research, University Hospital La Paz–IdiPAZ, Madrid, Spain; ^7^Department of Dermatology, King’s College Hospital, London, United Kingdom; ^8^Reference Center for Toxic and Autoimmune Blistering Diseases, Hopital Henri Modor, University Paris-Est, Créteil, France; ^9^Department of Internal Medicine and Respiratory Diseases, Hôpital Cavale Blanche, Brest, France; ^10^Department of pharmacology, Hôpital Pitié-salpétrière, Paris, France

**Correspondence**: Maren Paulmann

*Clinical and Translational Allergy* 2016, **6(Suppl 3)**:P33

**Background**: Stevens–Johnson syndrome (SJS)/toxic epidermal necrolysis (TEN) is rare, but the most severe of all adverse drug reactions due to a high mortality, which is almost 50 % in TEN. Good evaluation of risk factors is essential for efficient pharmacovigilance.

**Materials and methods**: From January 2003 to December 2012 the international Registry of Severe Cutaneous Adverse Reactions (RegiSCAR) included 1096 cases of SJS/TEN collected in seven countries (France, Germany, Israel, Italy, Netherlands, Spain, United Kingdom). The diagnoses and dates of onset were validated by an expert group, blinded for any information on exposures. Causality was established by using the specific algorithm ALDEN that had previously shown a good correlation with the results of a case–control analysis in an earlier systematic collection of several hundred cases.

**Results**: In terms of overall causality assessment, at least one medication was evaluated as the «probable» or «very probable» cause in 744/1096 cases (68 %). A medication cause was determined as «possible» in 209 cases (19 %), as «unlikely» in 68 cases (6.2 %) and “very unlikely” in 57 cases (5.2 %). 18 patients (1.6 %) denied any exposure to medications. The five medications most often incriminated («probable» or «very probable» causality) were allopurinol (n = 187; 17 %), sulfamethoxazole (n = 80), lamotrigine (n = 76), carbamazepine (n = 51), phenytoin (n = 42). There were substantial differences between reactions developing among hospitalized and community patients, e.g. older age, exposure to a substantially higher number of drugs and a noticeable role for metamizole among inhospital cases in Germany. In both groups (inhospital and community cases) we identified a signal for proton pump inhibitors.

**Conclusions**: In spite of prior warnings in medical journals and towards regulatory agencies, allopurinol is still the principal cause of SJS/TEN in Europe, as it was 10 years ago. Off-label prescription of allopurinol for asymptomatic hyperuricemia has not decreased. Sulfamethoxazole and other anti-infective sulfonamides are still frequent inducers of SJS/TEN. Lamotrigine is now the third cause and the first one among antiepileptic drugs, independent of new indications such as bipolar disorder. Finally, it is important to note that at least 13 % of SJS/TEN-cases have no drug cause.

Investigation of other possible causes of such «idiopathic cases», e.g. infections, should be a priority.

### P34 Long-term sequelae in patients with Stevens–Johnson syndrome and toxic epidermal necrolysis: a 5-year analysis

#### Maren Paulmann^1^, Carmen Kremmler^1^, Peggy Sekula^2^, Laurence Valeyrie-Allanore^3^, Luigi Naldi^4^, Sylvia Kardaun^5^, Maja Mockenhaupt^1^

##### ^1^Dokumentationszentrum schwerer Hautreaktionen, University Medical Center, Freiburg, Germany; ^2^Institute of Medical Biometry and Medical Informatics, University Medical Center, Freiburg, Germany; ^3^Reference Center for Toxic and Autoimmune Blistering Diseases, Hopital Henri Modor, University Paris-Est, Créteil, France; ^4^Department of Dermatology, Papa Giovanni XXIII Hospital, Bergamo, Italy; ^5^Reference Center for Cutaneous Adverse Reactions, University Medical Center, Groningen, The Netherlands

**Correspondence**: Maren Paulmann

*Clinical and Translational Allergy* 2016, **6(Suppl 3)**:P34

**Background**: Stevens–Johnson syndrome (SJS) and toxic epidermal necrolysis (TEN) are rare, but severe reactions with blisters and erosions of skin and mucosae. Most often SJS/TEN is induced by drugs, less frequently by infections, whereas some cases are “idiopathic”. Severity is determined by skin detachment related to the body surface area (BSA): SJS < 10 %, TEN >30 %, and SJS/TEN-overlap 10–30 % of BSA. The high mortality rate is due to severity as well as age of the patient. Survivors frequently suffer from long-term sequelae, particularly of the mucosae.

**Materials and methods**: In order to evaluate frequency and extent of late sequelae in patients with SJS/TEN, a cohort study was performed. Patients with a validated diagnosis of SJS/TEN, who were included in the international Registry of Severe Cutaneous Adverse Reactions (RegiSCAR) between 2003 and 2007, were asked to answer a follow-up questionnaire.

**Results**: 112/233 completed follow-up questionnaires could be analyzed. 62 patients were diagnosed with SJS, 35 with SJS/TEN-overlap, and 15 with TEN. The mean age is 44 years, and approx. 60 % are women. More than 90 % of the patients suffer from sequelae after 5 years, while the percentage increases with severity of the reaction. Less than 50 % were able to completely return to their normal daily activities. Sequelae mainly affect skin (73 %), mucosa (57 %) and nails (52 %). Chronic sequelae of the eyes (67 %) are the main issue for the patients. They include increased photosensitivity, dry eyes, ingrowing eyelashes (trichiasis), excessive watery eyes (epiphora), inflammatory cicatrization up to blindness. Furthermore, patients describe sleep disturbances and nightmares (29 %). In addition, they are afraid of drug use (65 %), and 56 % of the patients avoid taking drugs, which may have a negative impact on their health. Although many patients consider professional psychological support helpful (54 %), only 9 % had received it.

**Conclusions**: This evaluation shows that the majority of patients who survived SJS/TEN suffer from long-term sequelae. An interdisciplinary approach is not only important in the acute stage of the disease, but also after discharge from the hospital, including regular check-ups by specialists for these rare severe reactions.

### P35 Major emotional complications and decreased health related quality of life among survivors of Stevens–Johnson syndrome and toxic epidermal necrolysis

#### Roni P. Dodiuk-Gad^1^, Cristina Olteanu^2^, Anthony Feinstein^3^, Rena Hashimoto^4^, Raed Alhusayen^4^, Sonia Whyte-Croasdaile^5^, Yaron Finkelstein^6^, Marjorie Burnett^7^, Shachar Sade^8^, Robert Cartotto^7^, Marc Jeschke^7^, Neil H. Shear^4^

##### ^1^Department of Dermatology, Ha’emek Medical Center, Afula, Israel; ^2^Faculty of Medicine, University of Toronto, Toronto, Canada; ^3^Department of Psychiatry, Sunnybrook Health Sciences Centre, Toronto, Canada; ^4^Division of Dermatology, Department of Medicine, Sunnybrook Health Sciences Centre, Toronto, Canada; ^5^SJS and TENS Group Canada-CAST International, Toronto, Canada; ^6^Paediatric Emergency Medicine, Clinical Pharmacology and Toxicology, The Hospital for Sick Children, Toronto, Canada; ^7^Ross Tilley Burn Centre, Sunnybrook Health Sciences Centre, Toronto, Canada; ^8^Department of Pathology, Sunnybrook Health Sciences Centre, Toronto, Canada

**Correspondence**: Roni P. Dodiuk-Gad

*Clinical and Translational Allergy* 2016, **6(Suppl 3)**:P35

**Background**: Stevens–Johnson Syndrome (SJS) and Toxic Epidermal Necrolysis (TEN) are life-threatening mucocutaneous reactions, predominantly drug-induced. In addition to these high mortality rates, early and late physical complications are common. No studies were conducted on the emotional status or the general Health-Related Quality of Life among survivors of SJS/TEN. We aimed to characterize the long-term emotional complications and health-related quality of life among SJS/TEN survivors.

**Materials and methods**: Patients older than 18 years who survived SJS/TEN were assessed using various parameters. Emotional assessment was conducted by three validated questionnaires: Impact of Events Scale-Revised, General Health Questionnaire, and Hospital Anxiety and Depression Scale. Health-Related Quality of Life was assessed by three validated questionnaires: Dermatology Life Quality Index, EQ-5D, Skindex-29 and one specially designed for the study.

**Results**: Our cohort consists of 17 patients with mean 51.6 ± 74.7 months (median = 9, range 1–228) following SJS/TEN. Eleven out of 17 (65 %) were found to have symptoms of post-traumatic stress (Impact of Events Scale-Revised, mean = 22.4 ± 19.9) and 5 (29 %) met the criteria for post-traumatic stress disorder, 12 (71 %) had psychological distress (General Health Questionnaire, mean total score = 4.6 ± 4.2) and 11 (65 %) had symptoms of a psychiatric clinical disorder (Hospital Anxiety and Depression Scale, mean total score = 14.5 ± 8.4). The Dermatology Life Quality Index indicated a moderate to extremely large effect on the lives of 9 (53 %) participants (mean total score = 6.9 ± 7.6). Skindex-29 indicated a mild-severe effect on Health-Related Quality of Life in 10 (59 %) participants (mean = 24.6 ± 21.5). Participants rated their general health at a mean of 66.2/100 ± 18.1 (EQ-5D VAS). Fourteen (82 %) participants reported that SJS/TEN decreased their current quality of life. Twelve (71 %) reported that SJS/TEN influenced their current emotional status. Eleven (65 %) reported that SJS/TEN influenced their current everyday activities.

**Conclusions**: Survivors of SJS/TEN suffer from severe long-term emotional complications and decreased health-related quality of life.

**Keywords**: Stevens–Johnson Syndrome; Toxic epidermal necrolysis; Emotional complications; SJS/TEN

### P36 Retrospective analysis of Stevens–Johnson syndrome and toxic epidermal necrolysis in Japanese patients: treatment and outcome

#### Naoko Takamura^1^, Yumiko Yamane^1^, Setsuko Matsukura^2^, Kazuko Nakamura^2^, Yuko Watanabe^1^, Yukie Yamaguchi^1^, Takeshi Kambara^2^, Zenro Ikezawa^1^, Michiko Aihara^1^

##### ^1^Department of Environmental Immuno-Dermatology## Yokohama City University, Yokohama, Japan; ^2^Department of Dermatology## Yokohama City University Medical Center, Yokohama, Japan

**Correspondence**: Naoko Takamura

*Clinical and Translational Allergy* 2016, **6(Suppl 3)**:P36

**Background**: Stevens–Johnson syndrome (SJS) and toxic epidermal necrolysis (TEN) are rare but severe adverse drug reactions with high mortality.

**Materials and methods**: To present the clinical characteristics of SJS and TEN in Japan and evaluate the efficacy of treatments, we retrospectively analyzed cases of SJS and TEN treated in Yokohama City University Hospital and Yokohama City University Medical Center between January 2000 and December 2015.

**Results**: 60 cases of SJS (24 males and 36 females; average age, 54.2 years) and 40 cases of TEN (19 males and 21 females; average age, 56.2 years) were included in this study.

31 cases of SJS (51.6 %) and all cases of TEN were caused by drugs. Hepatitis was the most common organ involvement in both SJS and TEN. Renal dysfunction, intestinal disorder, and respiratory disorder were also involved in some cases. The major complication was pneumonia and sepsis. All cases except for four cases were treated systemically with corticosteroids. In SJS 40 cases (66.6 %) were treated with corticosteroid alone, Steroid pulse therapy was performed in 40 % of SJS and 90 % of TEN. Plasmapheresis and/or immunoglobulin therapy was combined with steroid therapy performed 17 cases in SJS (42 %) and 26 cases in TEN (65 %). Average SCORTEN score was 1.13 in SJS and 2.32 in TEN. The mortality rate was 6 % and the rates for SJS and TEN were 1.7 and 12.5 %, respectively. The mortality rate was 0 % treated with combination of corticosteroid, plasmapheresis and immunoglobulin therapy in SJS and TEN. These were much lower than predicted mortality according to a severity-of-illness scoring system for TEN prognosis (SCORTEN) socre.

**Conclusions**: Treatment with steroid pulse therapy in combination with plasmapheresis and/or immunoglobulin therapy seems to have contributed to prognostic improvement in SJS/TEN.

**Keywords**: SJS; TEN

### P37 Severe physical complications among survivors of Stevens–Johnson syndrome and toxic epidermal necrolysis

#### Roni P. Dodiuk-Gad^1^, Cristina Olteanu^2^, Rena Hashimoto^3^, Hall Chew^4^, Raed Alhusayen^3^, Sonia Whyte-Croasdaile^5^, Yaron Finkelstein^6^, Marjorie Burnett^7^, Shachar Sade^8^, Robert Cartotto^7^, Marc Jeschke^7^, Neil H. Shear^3^

##### ^1^Department of Dermatology, Ha’emek Medical Center, Afula, Israel; ^2^Faculty of Medicine, University of Toronto, Toronto, Canada; ^3^Division of Dermatology, Department of Medicine, Sunnybrook Health Sciences Centre, Toronto, Canada; ^4^Department of Ophthalmology and Vision Sciences, Sunnybrook Health Sciences Centre, Toronto, Canada; ^5^SJS and TENS Group Canada-CAST International, Toronto, Canada; ^6^Paediatric Emergency Medicine, Clinical Pharmacology and Toxicology, The Hospital for Sick Children, Toronto, Canada; ^7^Ross Tilley Burn Centre, Sunnybrook Health Sciences Centre, Toronto, Canada; ^8^Department of Pathology, Sunnybrook Health Sciences Centre, Toronto, Canada

**Correspondence**: Roni P. Dodiuk-Gad

*Clinical and Translational Allergy* 2016, **6(Suppl 3)**:P37

**Background**: Stevens–Johnson Syndrome (SJS) and Toxic Epidermal Necrolysis (TEN) are considered the most severe types of cutaneous adverse reactions to drugs, with high morbidity and mortality rates. We aimed to characterize the long-term physical complications among SJS/TEN survivors.

**Materials and methods**: Patients older than 18 years who survived SJS/TEN were assessed by an interview and by skin, oral mucous membrane and detailed ophthalmic exam.

**Results**: Our cohort consists of 17 patients with mean 51.6 ± 74.7 months (median = 9, range 1–228) following SJS/TEN. The most commonly reported symptom among survivors was chronic fatigue/weakness (76 %). The most common cutaneous signs were post-inflammatory dyspigmentation in 77 % of participants, scars (46 %), and milia (15 %). The most common cutaneous symptoms were pruritus (53 %), photosensitivity (35 %), and dry skin (24 %). In the ophthalmic exam, dry eyes were the most common finding in 44 %. Other identified signs were: lid adhesions/symblepharon (33 %), chronic ocular surface inflammation (33 %), loss of visual acuity (22 %), chronic conjunctivitis (22 %), keratinization of the tarsal conjunctiva (22 %), lachrymal duct scarring (22 %), blindness (11 %), photophobia (11 %), ectropian and trichiasis (11 %), corneal abrasions/ulcers (11 %), conjunctival synechiae (11 %), and corneal neovascularization (11 %). The most commonly reported ocular symptoms were dry eyes in 47 % of participants; other symptoms included photophobia (35 %), loss of visual acuity (35 %), and ocular pain (24 %). Hair loss and nail loss were reported in 53 and 35 % of participants a few months after TEN, respectively. Other less common complications included genital synechiae in 18 % of female survivors, lupus (12 %), renal dysfunction (12 %), and fibromyalgia (6 %). Tinnitus, tenderness on soles of feet, abnormal ECG, and vocal cord dysfunction were each reported in 6 % of participants.

**Conclusions**: Survivors of SJS/TEN suffer from severe, long-term physical complications and require ongoing longitudinal medical follow-up.

**Keywords**: Physical complications; Stevens–Johnson Syndrome; Toxic epidermal necrolysis; Long-term complications

### P38 Stevens–Johnson syndrome/toxic epidermal necrolysis combined with haemophagocytic lymphohistiocytosis: a case report

#### Brittany Knezevic^1^, Una Nic Ionmhain^1^, Allison Barraclough^1^, Michaela Lucas^2^, Matthew Anstey^1^

##### ^1^Sir Charles Gairdner Hospital, Perth, Australia; ^2^Pathwest Laboratory Medicine, Queen Elizabeth II Medical Centre, Perth, Australia

**Correspondence**: Michaela Lucas

*Clinical and Translational Allergy* 2016, **6(Suppl 3)**:P38

**Background**: The combination of Stevens–Johnson syndrome/toxic epidermal necrolysis (SJS/TEN) with haemophagocytic lymphohistiocytosis (HLH) is life-threatening and usually fatal in adults. We report a rare case of a 36 year old male who survived these concomitant illnesses.

**Report**: The patient presented with a 1 week history of fevers and flu-like symptoms, with onset of a mucocutaneous rash on day five. He was admitted to a tertiary intensive care unit (ICU) with a diagnosis of SJS/TEN (20–25 % of total body surface area). The potentially implicated drugs (ibuprofen, codeine and amoxicillin) were promptly withdrawn, and he received immunosuppressive therapy with methylprednisolone, intravenous immunoglobulin and cyclosporin combined with supportive ICU care. He continued to systemically deteriorate at a time when his skin lesions stabilised. Recognition of hyperferritinemia (40,900 µg/l), unremitting fevers and pancytopenia in a critically ill patient, clinched the diagnosis of HLH which was confirmed on bone marrow biopsy. He received treatment with the HLH-94 chemotherapy protocol (including etoposide and dexamethasone). Sequential skin patch tests to ibuprofen and amoxicillin, performed 6 months post-discharge, were negative (codeine patches were not available). The patient’s negative patch tests were interpreted with caution, due to previous chemotherapy and the unavoidable delays to testing, both of which may have led to T lymphocyte depletion and false negative patch test results. The patient was advised to strictly avoid all non-steroidal anti-inflammatories, aspirin, codeine and amoxicillin (he had tolerated other opiates and beta-lactams in hospital).

**How this report contributes to current knowledge**: To our knowledge, this is the first report of an adult surviving a severe episode of HLH and SJS/TEN overlap (rather than SJS alone). This case highlights (1) the potential for HLH to coexist with SJS/TEN and the importance of early recognition and treatment, (2) the difficulties in interpreting patch tests in patients who have received marrow ablative therapies, and (3) the complexities of providing recommendations on drug avoidance to patients with severe cutaneous adverse drug reactions and negative patch test results. In vitro lymphocyte proliferation assays may be a useful alternative diagnostic tool in this clinical setting.

**Consent**: Written informed consent was obtained from the patient for publication of this abstract and any accompanying images.

## Poster Walk 5: Other organs/unexpected immune reactions (P39–P47)

### P39 A case report of patient with anti-tuberculosis drug-related severe liver failure

#### Toru Usui^1^, Xiaoli Meng^1^, John Farrell^1^, Paul Whitaker^2^, John Watson^2^, Neil French^1^, Kevin Park^1^, Dean Naisbitt^1^

##### ^1^MRC Centre for Drug Safety Science, Dept Molecular & Clinical Pharmacology, University of Liverpool, Liverpool, United Kingdom; ^2^Regional Adult Cystic Fibrosis Unit, St James’s Hospital, Leeds, United Kingdom

**Correspondence**: Toru Usui

*Clinical and Translational Allergy* 2016, **6(Suppl 3)**:P39

**Background**: Exposure to anti-tuberculosis drugs (ATDs) isoniazid (INH), ethambutol (ETB), pyrazinamide (PZA) and/or rifampicin (RIF) is associated with a mild elevation of liver enzymes that occasionally develops into severe liver injury. We have reported that INH-specific CD4+ T-cell clones circulate in patients with ATD-related liver injury, which suggests that the adaptive immune system is involved in the disease pathogenesis. This study details the nature of the drug antigen-specific T-cell response that develops in a fatal case of ATD-related liver failure.

**Materials and methods**: Peripheral blood mononuclear cells were isolated from a patient who had a severe liver reaction to ATD medications, ALT 514 IU/l and Bilirubin 30 mg/dl. A lymphocyte transformation test (LTT) and IFNγ-ELISPOT assay using ATDs were performed and clones were generated from ATDs by serial dilution. Drug-specific clones were identified in terms of cross-reactivity and dose-responsiveness and then characterized in terms of CD phenotype and cytokine secretion.

**Results**: Positive LTT against multiple drugs (INH, ETB, PZA, RIF, and activated ester of isonicotinic acid which forms isonicotinic amide adducts in the cell culture system) was observed. Over 700 T-cell clones were generated from INH, ETB, PZA, RIF or activated ester of isonicotinic acid treated peripheral blood mononuclear cells. Antigen-specific proliferative responses of CD4 +/CD8+ clones were identified with only ETB and RIF. Clones were highly specific and did not crossreact with other ATDs. Clones are activated to proliferate and secrete cytotoxic mediators (granzyme B, perforin) and effector cytokines (IFN-γ, Il-13).

**Conclusions**: In conclusion, cytotoxic ETB and RIF-specific T-cell clones have been identified in patient with ATD-related severe liver failure.

**Keywords**: Isoniazid; Drug-induced liver injury

### P40 Acute interstitial nephritis induced by ibuprofen

#### Ana Castro Neves, Susana Cadinha, Ana Moreira, J. P. Moreira Da Silva

##### Centro Hospitalar de Vila Nova de Gaia/Espinho, Vila Nova De Gaia, Portugal

**Correspondence**: Ana Castro Neves

*Clinical and Translational Allergy* 2016, **6(Suppl 3)**:P40

**Background**: Acute Interstitial Nephritis (AIN) is often drug induced and the most frequently implicated drugs are Non-steroidal anti-inflamatory drugs (NSAIDs) and antibiotics. We report the case of a patient with AIN induced by Ibuprofen.

**Report**: A 74 year-old-female was admitted at emergency department with a 10-day history of malaise, anorexia, nausea, diffuse myalgias, fever (38 °C) and flank pain, treated with Paracetamol, Ibuprofen and Ciprofloxacin for suspected Pyelonephritis, by her attending physician. At admission she was febrile (38 °C) and prostrated. Laboratory studies revealed: leukocytosis, acute kidney injury (serum creatinine 5.8 mg/dl, blood urea nitrogen 178 mg/dl) and positive C-reactive Protein; urinalysis with leucocituria, proteinuria and hematuria; negative blood culture. Chest X-ray and ultra sound of kidneys were normal. Treatment with Paracetamol, Ibuprofen and Ciprofloxacin was discontinued and she started Meropenem and intravenous fluids. Renal function improved and she was discharged after 3 days. One year later she was admitted to hospital with similar clinical presentation and diagnosed with AIN. Ten days prior to this admission she was treated with Ciprofloxacin and Ibuprofen for Phlebitis. After these two episodes she was again treated with Ciprofloxacin with good tolerance. She was then referred to our Drug Allergy Clinic for suspected hypersensitivity to Ibuprofen. Lymphocyte transformation test (LTT) with Ibuprofen was positive (stimulation index of 13.4 at 10 µg/ml). Drug provocation test (DPT) with the suspected drug was not performed. DPT and long-term challenge with alternative drugs (Paracetamol and Etoricoxib) were both negative.

**How this report contributes to current knowledge**: Renal biopsy is the only definitive method to establish the diagnosis of AIN. However, when diagnosis seems likely, a probable precipitating drug can be easily withdrawn or patient improves readily after withdrawal of the suspected drug, biopsy can be avoidable. Challenge with the suspected drug remains the gold standard for diagnosis of drug hypersensitivity. Since AIN is a severe hypersensitivity reaction, DPT with the suspected drug is contra-indicated. The obvious time relationship between Ibuprofen treatment and reaction development in both episodes, the reproducible clinical presentation and positive LTT, suggests Ibuprofen has been the culprit drug. In cases of severe reactions LTT seems to be a valuable diagnostic tool.

**Consent**: Written informed consent was obtained from the patient for publication of this abstract and any accompanying images.

### P41 Cetuximab induced acneiform rash—two case reports

#### Daniela Ledic Drvar^1^, Sandra Jerkovic Gulin^2^, Suzana Ljubojevic Hadzavdic^1^, Romana Ceovic^1^

##### ^1^Department of Dermatology and Venereology, University Hospital Center Zagreb and School of Medicine, Zagreb, Croatia; ^2^Department of Dermatology and Venereology, General Hospital Sibenik, Sibenik, Croatia

**Correspondence**: Sandra Jerkovic Gulin

*Clinical and Translational Allergy* 2016, **6(Suppl 3)**:P41

**Background**: Epidermal growth factor receptor inhibitors (EGFRIs) are used for treatment of advanced lung, pancreatic, colorectal, and head and neck cancers. Cetuximab is a monoclonal antibody that competitively binds to the extracellular domain of EGFR and blocks cytoplasmic-domain phosphorilation. It is characterized by frequent cutaneous adverse reactions (CARs) including acneiform rash (AR), desquamation, xerosis, pruritus, hair abnormalities, paronychia, changes in nails, mucositis, increased growth of facial hair and eyelashes, and teleangiectasias. AR usually appears between day 2 and week 6 after cetuximab Background. According to the *National Cancer Institute Common Toxicity Criteria*, severity of toxicity of AR is graded into five grades, and according to EGFRI-induced AR severity score into mild, moderate and severe toxicity.

Prophylactic antibiotic treatment with doxycycline 100 mg bid for the first 6 weeks is recommended. Therapeutic plan includes use of grade-based skin treatment algorithm: (a) mild toxicity—local therapy with corticosteroids or antibiotics, (b) moderate toxicity—local therapy with addition of oral antibiotic, and (c) severe toxicity—cetuximab dose lowering and therapy for moderate toxicity with an addition of a methyprednisolone dose pack. If therapy is ineffective within 2–4 weeks, it is necessary to discontinue cetuximab treatment.

**Report**: We present two male patients with metastatic colorectal cancer who developed papulopustular lesions on the sebum-rich areas of the face, scalp and trunk accompanied by pruritus and burning sensations (grade 2/3 or moderate toxicity), 1–3 weeks after Background of cetuximab. One patient developed desquamation of the palms. Comedones, primary acne lesions, were lacking. There was no sign of superinfection. Patients were treated with doxycycline 2 × 100 mg bid for 2 weeks, followed by 1 × 100 mg, until the end of cetuximab therapy. Local therapy included clindamycin lotion, hydrocortisone/oxytetracycline ointment, benzoyl peroxide suspension and emollients. After 3 weeks of treatment, significant improvement of AR severity was achieved and there was no need for cetuximab dose reduction.

**How this report contributes to current knowledge**: Studies have demonstrated positive correlation between treatment efficacy and CARs for EGFRI. Since frequency and severity of skin lesions are dose-dependent, a gradual increase in dose until CARs develop might be a good strategy to maximize the efficacy of EGFRI.

**Consent**: Written informed consent was obtained from the patient for publication of this abstract and any accompanying images.

### P42 Enteropathy associated with losartan

#### Ana Montoro De Francisco, Talía De Vicente Jiménez, Amelia García Luque, Natalia Rosado David, José Mª Mateos Galván

##### Hospital Central de la Defesa, IMIDEF, Madrid, Spain

**Correspondence**: Ana Montoro De Francisco

*Clinical and Translational Allergy* 2016, **6(Suppl 3)**:P42

**Background**: On August 2012 Rubio-Tapia et al. reported an association of olmesartan therapy with an unexplained enteropathy symptomatically resembling celiac disease or sprue. Olmesartan is an Angiotensin II Receptor Antagonist (ARA II) widely used in high blood pressure, heart disease and diabetics. On July 2013 the Food Drug Administration (FDA) approves label changes to include intestinal problems, Severe Spruelike Enteropathy (SSE) linked to blood pressure medicine olmesartan.

Losartan was the first ARA II commercialized in 1995 and it seems to produce similar Adverse Drug Reactions (ADR) that olmesartan.

**Report**: Method

Desing: Case report of enteropathy associated with losartan

Scope: Allergy Service, Hospital Central de la Defensa, Madrid.

Period: May 2015

Main variables assessed: demographic and clinical variables, diagnostic criteria, treatment, evolution, causal relationship between drug and enteropathy according to the modified Karch Lasagna algorithm.

The patient has given written informed consent for the publication research.

**Results:** A 81 years old woman, diagnosed of hypertension, treated for 3 years with losartan 100 mg/d. After starting treatment she refers chronic diarrhea and since 2 years ago worsening diarrhea, abdominal pain and weight loss. The patient was treated at the emergency service twice for 3–4 daily episodes of watery, nonbloody diarrhea associated with abdominal bloating. The patient failed conservative management including gluten-free diet, oral antibiotic and corticosteroid. Additionally to diarrheal syndrome, the patient showed hoarseness and cough.

Allergologic study: immediate and delayed prick test and specific IgE were negative for food.

A colonoscopy with colonic biopsies revealed evidence of microscopic colitis.

Treatment: Losartan withdrawal, achieving complete remission in 4 weeks.

The case has been reported to Spanish Postmarketing Surveillance System.

**How this report contributes to current knowledge**: The allergist should keep in mind this possible and rare ADR in patients treated with losartan (ARA II) showing enteropathy, as discontinuing losartan leads to clinical improvement.

This enteropathy case is very similar to SSE described in 2012 related to olmesartan.

**Consent**: Written informed consent was obtained from the patient for publication of this abstract and any accompanying images.

### P43 Granuloma annulare after therapy with canakinumab

#### Razvigor Darlenski

##### Tokuda Hospital Sofia, Sofia, Bulgaria

**Correspondence**: Razvigor Darlenski

*Clinical and Translational Allergy* 2016, **6(Suppl 3)**:P43

**Background**: Granuloma annulare (GA) is a benign inflammatory dermatosis affecting predominantly females. The disease etiology and pathophysiology remain unclear. Trauma, association with diabetes mellitus and medicamentous genesis have been suspected. Pathogenic mechanisms include cell-mediated immunity (type IV), macrophage activation, and cytokine-mediated degradation of connective tissue.

**Report**: Herein we present a 56-years-old male Caucasian patient who developed skin lesions on the back of the hand 3 months after initiation of therapy with canakinumab as a part of a clinical trail for treatment of coronary artery disease. Upon admission the skin changes involved the dorsum of the hand and were presented by annular papules and plaques, the largest 3 cm in diameter with depressed center and elevated and infiltrated borders. The patient had no other relevant history of present or past disease beyond the coronary artery diseases and elevated serum cholesterol levels. He took no other medication prior or during the disease onset.

Based on the clinical findings the diagnosis of GA was coined. Cryotherapy with liquid nitrogen was performed. Canakinumab was withdrawn. Skin lesions resolved within the 2 weeks later. No new lesions appeared during the 6 months follow up.

**How this report contributes to current knowledge**: Canakinumab is a IL-1 beta monoclonal antibody used in rheumatology and in the treatment of autoinflammatory syndrome. Recently it was tested in coronary artery disease, gout and COPD. As a new drug, its safety profile and unwanted adverse events are still to be challenged. In the described case we present the development of cutaneous reaction coinciding with the drug Background. Drug-induced GA has been described in the literature, e.g. after allopurinol, amlodipine, TNF-alpha inhibitors, gold, and interferon therapy. As far as we are aware no previous reports exist on the association between canakinumab therapy and GA.

**Consent**: Written informed consent was obtained from the patient for publication of this abstract and any accompanying images.

### P44 Hypersensitivity eosinophilic myocarditis or acute coronary syndrome? Case report

#### Dario Gulin^1^, Jozica Sikic^1^, Jasna Cerkez Habek^1^, Sandra Jerkovic Gulin^2^, Edvard Galic^1^

##### ^1^University Hospital Sveti Duh, Zagreb, Croatia; ^2^General Hospital Sibenik, Sibenik, Croatia

**Correspondence**: Sandra Jerkovic Gulin

*Clinical and Translational Allergy* 2016, **6(Suppl 3)**:P44

**Background**: Eosinophilic myocarditis (EM) is a potentially fatal disease if left untreated. There are numerous drugs that have been implicated in causing the hypersensitivity (HS) form of EM but frequently the cause of the disease remains unknown as it could have a delayed presentation for up to 2 years. In this case report we present an atypical clinical presentation of HS EM, possibly caused by hydrochlorothiazide (HCT), and significant coronary artery disease (CAD).

**Report**: A 63-year-old man presented to the emergency department with flu-like symptoms. He had a history of chronic bronchitis and arterial hypertension treated with budesonide/formoterol and HCT. His general physical examination was unremarkable, without any signs of heart failure. Complete blood count (CBC) revealed leucocytosis with slightly eosinophilia and elevated troponin and CRP. ECG showed no changes in ST/T segment. Echocardiography revealed minimal pericardial effusion for up to 4 mm. Initial right pneumonia was observed on X-ray. He was treated with amoxicillin/clavulanic acid, azithromycin and ibuprofen. Fifth day of hospitalization the signs of disease became more prominent: CBC revealed 19.2 × 10^9^/l leucocytes with 11.2 × 10^9^/l eosinophils and troponin level was doubled. Pulse doses of corticosteroids (CS) (methylprednisolone 500 mg i.v.) were started and other therapy was discontinued. Seven hours later patient was asymptomatic, leucocytes were normal with slightly eosinophilia. Pulse doses of CS were continued up to 3 days when converted to oral CS. Endomyocardial biopsy showed no inflammation. Coronarography revealed two-vessel CAD, treated with percutaneous coronary intervention.

**How this report contributes to current knowledge**: One of the most common cause of EM reported in literature is drug HS. In our patient HCT is a potential cause. Symptoms, levels of troponin, ECG and echocardiographic changes were more suggestive for EM than CAD. Endomyocardial biopsy revealed no EM but it was performed third day after pulse dosage of intra-venous CS, when symptoms completely vanished. It should be also noted that EM is a focal disease and biopsy doesn’t have to prove the diagnosis. It is more obvious that CAD was co-finding, as symptoms of CAD could not be resolved using CS, and troponin levels would not fall second day after initiating therapy. CBC with eosinophilic drop powers that hypothesis. Early administration of systemic CS is necessary regardless of underlying causes, as delayed treatment may result in fatal outcomes.

**Consent**: Written informed consent was obtained from the patient for publication of this abstract and any accompanying images.

### P45 Piperacillin-induced immune haemolytic anaemia: a severe and frequent complication of antibiotic treatment in patients with cystic fibrosis

#### Philip Specht^1^, Doris Staab^1^, Beate Mayer^2^, Jobst Roehmel^1^

##### ^1^Division of Cystic Fibrosis, Pediatric Pneumology and Immunology, Charité-Universitätsmedizin Berlin, Berlin, Germany; ^2^Institute for Transfusion Medicine, Charité - Universitätsmedizin Berlin, Berlin, Germany

**Correspondence**: Philip Specht

*Clinical and Translational Allergy* 2016, **6(Suppl 3)**:P45

**Background**: Two mechanisms of drug-induced haemolysis have been described. Usually both, extravascular and intravascular haemolysis, are caused by drug dependent antibodies (ddAb). Extravascular haemolysis presents with mild and less severe clinical symptoms of anaemia, whereas intravascular haemolysis usually manifests with a rapid destruction of red blood cells caused by antibody-mediated complement activation leading to acute clinical symptoms. Interestingly more than 50 % of all piperacillin-induced immune haemolytic anaemia (PIHA) cases investigated at a reference laboratory in Germany were patients with cystic fibrosis (CF). Therefore the investigated hypothesis of this study is a higher prevalence for PIHA in patients with CF as for the general population. Additionally risk factors for PIHA are systematically assessed.

**Materials and methods**: Included were all patients with CF older than 12 years who were admitted to the hospital for antibiotic treatment. Past transplantation was an exclusion criterion. Blood samples were obtained in the beginning and at the end of a given intravenous treatment. Direct antiglobulin test (DAT) and indirect antiglobulin test (IAT) were performed using standard techniques. For positive samples an antibody differentiation was done. DdAb were investigated using gel technique. Thus we prepared a 1 mg/ml drug solution or used ex vivo antigens, respectively and incubated it for 30 min in presence of patients’ plasma and group O RBCs. The individual prior exposure to piperacillin and information concerning atopy and microbiology are documented.

**Results**: In the ongoing trial 2 out of 20 patients developed a PIHA. This is a high prevalence compared to the general expectation of approximately 1:1,000,000. Both had ddAb but no complement activation. This is typical for extravascular haemolysis. Clinical symptoms of haemolysis were mild but there was a drop of haemoglobin by 4 mg/dl. The study will be finished by April 2016.

**Conclusions**: These results suggest a higher prevalence of PIHA in patients with CF than expected in the general population. This is backed by several case reports. Possibly this is due to the exposure to piperacillin. Therefore PIHA should be considered when treating patients with CF.

**Keywords**: Piperacillin; Autoimmune; Haemolysis; Cystic fibrosis

### P46 Progesterone triggered pemphigus foliaceus: case report

#### Sandra Jerkovic Gulin^1^, Caius Solovan^2^, Anca Chiriac^3^

##### ^1^Department of Dermatology and Venereology, General Hospital Sibenik, Sibenik, Croatia; ^2^Department of Dermatology, University of Medicine and Pharmacy, Tamisoara, Romania; ^3^Dermatology Department, Nicolina Medical Centre, Apollonia University, “P.Poni” Research Institute of Macromolecular Chemistry, Iasi, Romania

**Correspondence**: Sandra Jerkovic Gulin

*Clinical and Translational Allergy* 2016, **6(Suppl 3)**:P46

**Background**: Pemphigus foliaceus (PF) is a benign variety of pemphigus (P) characterized by acantholysis in the upper part of epidermis, induced by immunoglobulin G (IgG) autoantibodies (mainly IgG4 subclass) targeting desmoglein -1 (dsg-1). Clinical expression of PF is based on the presence of scaly and crusted erosions on an erythematous base, flaccid blisters, minor or no involvement of mucous membranes and with seborrheic distribution and aspect of the lesions in early stages of the disease. PF is classified into four types: endemic (fogo selvagem), idiopathic, erythematosus (Senear-Usher syndrome) and drug-induced. Drugs triggering or inducing PF reported in literature are penicillamine, bucilamine, captopril, lisinopril, enalapril, fosinopril, candesartan, tipronin and rifampicin.

**Report**: A 45-year-old woman presented with facial erythema, multiple erythematous papules and plaques, few scaly plaques, shallow erosions and superficial blisters on an erythematous base localized on the upper chest and upper back in “V” distribution, acompanied by pain and burning sensation. Mucous membranes were spared. Lesions appeared 2 months after initiating systemic therapy with medroxyprogesterone acetate 10 mg daily for 14 days per month for metrorrhagia. Biopsy revealed subcorneal cleft within the upper epidermis with acantholytic keratynocytes. Direct immunofluorescence (DIF) of perilesional skin showed intercellular, intraepithelial IgG. Indirect immunofluorescence (IIF) showed intercellular, intraepithelial IgG, with higher fluorescent intensity in the upper dermis. The diagnosis of possible drug induced PF was presumed based on clinical, histological and immunological grounds. The suspected culprit drug—progesterone was discontinued. Treatment with high-potency topical corticosteroid (betamethasone) in combination with gentamicin ointment twice daily was started. After 4 weeks of treatment, lesions resolved almost completely. Close follow-up was recommended.

**How this report contributes to current knowledge**: Although PF often occurs spontaneously, it may be triggered by certain medications. A case of localized PF induced by topical imiquimod treatment has been recently reported, based on an unknown mechanism of acantholysis, probably due to antibodies to dsg-1. Drug as a trigger must be suspected in every newly diagnosed P. Indentification and elimination of triggering drug may soothe the clinical symptoms and shorten the treatment.

**Consent**: Written informed consent was obtained from the patient for publication of this abstract and any accompanying images.

### P47 Ramipril: triggered generalized pustular psoriasis

#### Paola Djurinec^1^, Kresimir Kostovic^1^, Mirna Bradamante^1^, Sandra Jerkovic Gulin^2^, Romana Ceovic^1^

##### ^1^Department of Dermatology and Venereology, University Hospital Center Zagreb and School of Medicine Zagreb, Zagreb, Croatia; ^2^Department of Dermatology and Venereology, General Hospital Sibenik, Sibenik, Croatia

**Correspondence**: Sandra Jerkovic Gulin

*Clinical and Translational Allergy* 2016, **6(Suppl 3)**:P47

**Background**: Generalized pustular psoriasis (GPP) is an extreme and serious form of psoriasis (P) with tiny, sterile, yellow pustules on an erythematous base. The clinical presentation of drug-provoked P include more often generalized plaque P and erythroderma, and rarely palmoplantar pustulosis or GGP. Drugs can cause (a) precipitation of P de novo in predisposed or nonpredisposed individuals; (b) exacerbation of pre-existing psoriatic lesions; (c) induction of lesions in clinically normal skin in patients with P; and (d) development of treatment-resistant P. Angiotensin-converting enzyme inhibitors (ACEIs) are widely used to control hypertension and are considered to be possible triggering, exacerbating or inducing agents of P. Rarely, ACEIs have been reported in the literature as inductors of GPP. We present our first case of ramipril-triggered GPP.

**Report**: 46-year-old women presented with low-grade fever, erythema, small pustules 1–2 mm in size and exfoliation involving skin on trunk, extremities, palms and soles. She had no previous history of P or family history of P. She began taking the new antihypertensive—ramipril 2 months before the onset of symptoms. Laboratory findings showed elevated erythrocyte sedimentation rate and C-reactive peptide with other biochemical findings within normal limits. Gram stained smear examination of pustules were negative and the pus culture was sterile. We set up a working diagnosis of GPP based on history, laboratory findings and clinical picture and the main differential diagnosis was acute generalized exanthematous pustulosis. Biopsy was performed. Ramipril as suspected culprit drug was discontinued and replaced with valsartan. We started the treatment with acitretin 50 mg/day which resulted in good initial therapeutic response. On the basis of clinical picture, biopsy and satisfactory therapeutic outcome, the diagnosis of GPP was confirmed.

**How this report contributes to current knowledge**: Only one case of ramipril-induced GPP in patient with psoriatic arthritis has been published in the literature. According to our knowledge, we present a first case of ramipril-triggered GPP in an individual without personal and family history of P. As P is a common skin disorder, knowledge of the factors that are inducers, triggers, or aggravators of the disease is of utmost importance for clinicians. Drug cessation or replacement may play a crucial role in the treatment outcome so taking a detailed history is decisive.

**Consent**: Written informed consent was obtained from the patient for publication of this abstract and any accompanying images.

## Poster Walk 6: NSAIDs (P48–P56)

### P48 Aspirin desensitization in cardiovascular disease—Portuguese experience

#### Jose Pedro Almeida^1^, Joana Caiado^1^, Elisa Pedro^1^, Pedro Canas Da Silva^2^, Manuel Pereira Barbosa^1^

##### ^1^Immunoallergology Department, Centro Hospitalar Lisboa Norte/Hospital Santa Maria, Lisbon, Portugal; ^2^Cardiology Department, Centro Hospitalar Lisboa Norte/Hospital Santa Maria, Lisbon, Portugal

**Correspondence**: Joana Caiado

*Clinical and Translational Allergy* 2016, **6(Suppl 3)**:P48

**Background**: Several studies have demonstrated a reduction in adverse cardiovascular events with the administration of dual antiplatelet therapy (aspirin and clopidogrel) to patients (pts) with coronary artery disease, especially in the prevention of thrombosis after implantation of bare-metal and drug-eluting stents. However, when pts have aspirin hypersensitivity (AH) this treatment is limited, and they are maintained on monotherapy with clopidogrel. Aspirin desensitization (AD) is the alternative for these individuals. The aim is to describe the experience of a Portuguese Allergy Department in AD on cardiovascular pts, between April 2006 and January 2016.

**Materials and methods**: Thirty pts with previous history of AH who needed dual antiplatelet therapy due to cardiovascular disease (female-14, male-16), mean age 64 years-old, were desensitized to aspirin. The clinical presentation of AH was asthma (six pts), anaphylaxis (four pts), cutaneous rash (seven pts), angioedema (13 pts), in some cases with very remote reactions (>20 years ago). In most pts (22 out of 30), AD was performed immediately after percutaneous coronary intervention (PCI). Most AD were performed in Cardiology Department (27); three pts with planned AD were desensitized at the Allergy Day-Care Unit; oral doses were increased every 30–60 min (target dose 152.5 mg) under a 4.5-h protocol. Pre-medication was not administered.

**Results**: All pts successfully completed AD, although there were two reactions 30 min after the procedure: a 63 year-old man who developed acute rhinitis and conjuntivitis, eyelid angioedema and dry cough; and a 75 year-old man who developed acute generalized urticaria. All pts were monitored after AD, and are still on daily aspirin 100 mg (there were neither hypersensitivity reactions nor drop-outs).

**Conclusions**: Despite positive clinical history of AH, some pts had a remote reaction, and due to the emergency of the procedure we were not able to confirm their AH. This fact could in part explain the low incidence of reactions during AD; another reason could be the smaller dose of aspirin used as antiplatelet (100–150 mg) compared with the dose to which they previously reacted (>500 mg). Some authors advocate that AD should be performed before PCI, but PCI could provide greater hemodynamic stability and therefore allow a safer AD. Based on our experience, AD is safe and efficacious, even when performed after PCI, with all pts responding to the desensitization procedure.

**Keywords**: Aspirin hypersensitivity; Drug desensitization; Anaphylaxis

**Consent**: Written informed consent was obtained from the patient for publication of this abstract and any accompanying images.

### P49 Asthma and/or rhinitis to NSAIDs with good tolerance to ASA

#### Gador Bogas^1^, Natalia Blanca-López^2^, Diana Pérez-Alzate^2^, Inmaculada Doña^1^, José Augusto Agúndez^3^, Elena García-Martín^3^, José Antonio Cornejo-García^4^, Cristobalina Mayorga^4^, María José Torres^1^, Gabriela Canto^2^, Miguel Blanca^5^

##### ^1^Allergy Unit, IBIMA, Regional University Hospital of Malaga, UMA, Malaga, Spain; ^2^Allergy Service, Infanta Leonor University Hospital, Madrid, Spain; ^3^Department of Pharmacology, University of Extremadura, Caceres, Spain; ^4^Research Laboratory and Allergy Unit, IBIMA, Regional University Hospital of Malaga, UMA, Malaga, Spain; ^5^Allergy Unit, IBIMA, Regional University Hospital of Malaga, UMA, Malaga, Spain

**Correspondence**: Gador Bogas

*Clinical and Translational Allergy* 2016, **6(Suppl 3)**:P49

**Background**: Subjects with hypersensitivity reactions to NSAIDs with airways involvement can develop rhinitis and or asthma after the intake of ASA and other NSAIDs that are strong COX-1 inhibitors. Some cases may also respond to weak COX-1 and selective COX-2 inhibitors. The hallmark characteristic is that symptoms are triggered by asthma and that in most instances subjects have a pre-existing respiratory disease. We studied if subjects can be selective responders to some NSAIDs including weak COX-1 or selective COX-2 inhibitors having good tolerance to ASA.

**Materials and methods**: Subjects reporting rhinitis and/or asthma to a single NSAID with good tolerance to ASA were evaluated. An allergological evaluation was carried out plus a controlled challenge with ASA for verifying tolerance. In a second step we proceed to a controlled challenge with the culprit drug involved. FEV1 and acoustic nasal rhynomanometry were measured with values lower that 12 and 30 % respectively considered as a positive response. Also nasal and bronchial symptoms were recorded.

**Results**: We confirmed in 21 cases the appearance of nasal and/or bronchial symptoms by objective parameters (decrease in FEV1 and/or decrease in nasal volume cavity) after the administration of the drug. The drugs involved were ibuprofen in four cases, paracetamol in four cases, desketoprofen in one and etoricoxib in another.

**Conclusions**: Selective respiratory responses to a single NSAID with good tolerance to ASA occur. Although the underlying mechanism is not known, the classical involvement of the leukotriene’s pathway occurring is aspirin exacerbated respiratory disease seems to be discarded. This constitutes a new phenotype within the category of hypersensitivity reactions to NSAIDs.

### P50 Clinical characteristics of 196 patients with non-steroidal anti-inflammatory drug (NSAIDs) hypersensitivity

#### Sengül Aksakal^1^, Aytül Zerrin Sin^2^, Zeynep Peker Koç^2^, Fatma Düsünür Günsen^2^, Ömür Ardeniz^2^, Emine Nihal Mete Gökmen^2^, Okan Gülbahar^2^, Ali Kokuludag^2^

##### ^1^Karadeniz Technical University Medical Faculty Department of Clinical Immunology, Trabzon, Turkey; ^2^Ege University Medical Faculty Department of Allergy and Clinical Immunology, Izmir, Turkey

**Correspondence**: Aytül Zerrin Sin

*Clinical and Translational Allergy* 2016, **6(Suppl 3)**:P50

**Background**: Nonsteroidal antiinflammatory drugs (NSAIDs) as the most frequently prescribed classes of drugs have been reported to be the second most common cause of drug hypersensitivity reactions after beta-lactam antibiotics. In this study we aimed to evaluate the clinical characteristics of NSAID hypersensitivity based on European Network for Drug Allergy (ENDA)’s classification.

**Materials and methods**: 196 patients diagnosed as NSAIDs hypersensitivity between the years of 2013–2014 were enrolled to the study. The patients were questioned about the other allergic diseases (rhinitis, asthma, urticarial, personal and familial drug allergy) besides the demographic features. NSAID reactions were classified as follow: NSAID induced urticaria/angioedema (NIUA), single NSAID induced urticaria/angioedema, anaphylaxis or both (SNIUAA), NSAID exacerbated cutaneous disease (NECD), NSAIDs exacerbated respiratory disease (NERD), single NSAID induced delayed hypersensitivity reactions (SNIDR). Acetylsalicylic acid, diclofenac, paracetamol and meloxicam were the most frequently preferred drugs for oral provocation test to distinguish of cross-reactive and IgE-mediated reactions.

**Results**: Hypersensitivity to NSAIDs was three times common in female (77 %) than male (23 %). The patients with NERD had atopic history the most frequently (50 %). 6.6 % of patients have drug allergy in their family. The frequency of hypersensitivities was found respectively as follows: NIUA (46 %), SNIUAA (24 %), NECD (15 %), NERD (8 %), SNIRD (7 %). The patients who had cross-reactive hypersensitivity to many of NSAIDs could be tolerated paracetamol, meloxicam and nimesulid. Diclofenac (11 cases 23 %), Flurbiprofen (seven cases 15 %), Metamizol (seven cases 15 %) were induced SNIUAA respectively, But according to their chemical structure propionic acid derivatives had been caused severe anaphylaxis mostly. Commonly seen delayed reactions due to NSAIDs are maculopapular eruptions and fixed drug eruption.

**Conclusions**: NSAIDs were caused NIUA, SNIUAA and NECD most frequently (85 %) although NERD and delayed reactions were quite rare. There are still unexplained points in these cases. Therefore there are needed more clinical studies.

**Keywords**: Non-steroidal anti-inflammatory drug hypersensitivity

### P51 Development of immediate hypersensitivity to several NSAIDs maintaining good tolerance to ASA

#### Natalia Pérez-Sánchez^1^, Natalia Blanca-López^2^, Diana Pérez-Alzate^2^, Gador Bogas^1^, Inmaculada Doña^1^, María Salas^1^, María José Torres^1^, Miguel Blanca^1^, Gabriela Canto^2^

##### ^1^Allergy Unit, Malaga Regional University Hospital-IBIMA, Malaga, Spain; ^2^Allergy Service, Infanta Leonor Hospital, Madrid, Spain

**Correspondence**: Natalia Pérez-Sánchez

*Clinical and Translational Allergy* 2016, **6(Suppl 3)**:P51

**Background**: Subjects responding to several NSAIDs including ASA are considered cross-hypersensitive, whereas those responding to only one NSAID with tolerance to other chemically unrelated NSAIDs are considered selective responders. However, in some cases, patients that develop immediate reactions to several NSAIDs are classified as cross-hypersensitive without assessing ASA tolerance.

**Report**: A 70 year-old woman, without allergic history, referred that in 1996 she developed two episodes of urticaria less than an hour after paracetamol intake. Three years after, she had developed three episodes of urticaria 90 min after dipyrone intake. At that time she was allergologically evaluated: intradermal test (ID) with dipyrone (2 mg/ml) and paracetamol (0.1 mg/ml) were positive. She was challenged with ASA showing tolerance at full therapeutic doses (500 mg). In 2012, she suffered from an episode of facial angioedema and pruritus in the tongue after celecoxib intake. She reported previous tolerance to this drug on many occasions. In September 2014, she was re-evaluated: ID to paracetamol was negative (0.1 mg/ml), and ID and basophil activation test (BAT) to dipyrone were positive. Drug challenges: 60 min after achieving a cumulative dose of paracetamol 300 mg, she developed generalized pruritus and urticaria. Two weeks later, she presented pruritus in the tongue and ears, nasal itching and obstruction, and wheals in the neck after provocation with a cumulative dose of 100 mg of celecoxib, with no changes in FEV1 values. After another interval of 2 weeks tolerance to etoricoxib was observed at full therapeutic doses. Two weeks later the patient tolerated ASA and ibuprofen during controlled challenge until therapeutic doses. In addition, around 2 months later the patient received two independent 2 days treatment courses with these drugs, separated by a 2 weeks time interval.

**How this report contributes to current knowledge**: Subjects may develop an immediate response to a number of different NSAIDs, including some COX-1 and/or selective COX-2 inhibitors but tolerate strong COX-1 inhibitors. This case shows the importance of testing for ASA tolerance when confronted by a patient who responds to several chemically unrelated NSAIDs.

**Consent**: Written informed consent was obtained from the patient for publication of this abstract and any accompanying images.

### P52 Diagnosis of hypersensitivity reactions to paracetamol in a large serie of cases

#### Inmaculada Doña^1^, Maria Salas^1^, Francisca Gomez^1^, Natalia Blanca-Lopez^2^, Diana Perez-Alzate^2^, Gador Bogas^3^, Esther Barrionuevo^1^, Maria Jose Torres^1^, Inmaculada Andreu^4^, Miguel Ángel Miranda^4^, Gabriela Canto^2^, Miguel Blanca^1^

##### ^1^Regional Hospital of Málaga-IBIMA, Málaga, Spain; ^2^Infanta Leonor Hospital, Madrid, Spain; ^3^Regional Hospital of Málaga, Málaga, Spain; ^4^Dpto. Química/Instituto de Tecnología Química –UPV-CSIC, Valencia, Spain

**Correspondence**: Inmaculada Doña

*Clinical and Translational Allergy* 2016, **6(Suppl 3)**:P52

**Background**: The most important drug involved in hypersensitivity drug reactions (HDR) are NSAIDs. Two mechanisms are involved: immunological sensitization (specific IgE or T-cell) and pharmacological (COX-1 inhibition). Paracetamol, one of the drug most highly consumed all over the world, has been implicated in HDR. The contribution these mechanisms in HDR to paracetamol is not well known. We studied a large group of patients who developed one/several episodes indicative HDR to paracetamol.

**Materials and methods**: Patients with reactions to paracetamol were classified as cross-hypersensitivity (CH) if they responded to two others NSAIDS not chemically related including ASA. If only paracetamol was implicated, drug provocation test (DPT) with ASA was required: if positive, they were included as CH and if negative, as selective responders (SR). In cases who reported respiratory symptoms, nasal provocation test with L-ASA were performed. Atopy status was also assessed with prick test to inhalant allergens.

**Results**: A total of 350 patients with confirmed diagnosis of HDR to paracetamol were included: 288 (82.28 %) resulted to be CH and 62 SR (17.71 %). Within the CH group, following the ENDA classification, 181 (62.84 %) were NIUA, 25 (8.68 %) NERD, 16 (5.55 %) NECD, and in 60 (20.83 %) blended reactions. A total of 46 (25.41 %) patients required a positive DPT for diagnosis. Nasal challenge with L-ASA was positive in 19 (82.6 %) patients with clinical history suggestive of NERD. Within SR group, 56 cases (90.32 %) were SNIUAA (reaction less than 24), being anaphylaxis the most frequent clinical entity (29 (51.78 %), followed by urticaria/angioedema (16; 28.57 %), angioedema (6; 10.71 %), urticaria (4; 7.14 %) and fixed drug eruption (1; 1.78 %). A total of 18 (32.14 %) patients required a positive DPT to paracetamol for diagnosis. The remaining six (9.67 %) cases were SNIDHR as they had the reaction more than 24 h after paracetamol administration: four (66.66 %) maculopapular exanthema and two (33.33 %) exanthema with desquamation. One patient had respiratory symptoms and was confirmed as SR.

**Conclusions**: These data indicate that when patients develop a HDR to paracetamol after carried out the allergological work-up, the 80 % of patients who developed a positive response were CH with a lower proportion being SR. DPT was needed in more than 20 % of cases to achieve the diagnosis.

### P53 Hypersensitivity to paracetamol according to the new classification of hypersensitivity to NSAIDs

#### Gabija Didžiokaite^1^, Olesia Gaidej^2^, Simona Kašinskaite^3^, Violeta Kvedariene^3^

##### ^1^Vilnius University, Faculty of Medicine, Vilnius, Lithuania; ^2^Vilnius University Hospital Santariskiu Klinikos, Centre of Internal Medicine, Vilnius, Lithuania; ^3^Center of Pulmonology and Allergology, Clinic of Infectious, Chest diseases, Dermatology and Allergology, Vilnius University Hospital Santariskiu Klinikos, Vilnius, Lithuania

**Correspondence**: Gabija Didžiokaite

*Clinical and Translational Allergy* 2016, **6(Suppl 3)**:P53

**Background**: Paracetamol is often proposed as a safe alternative to the patients with hypersensitivity to NSAIDs. However, reactions induced by this drug can manifest in a wide variety of symptoms.

**Aim**: To evaluate hypersensitivity to paracetamol in Lithuanian adult population according to the new classification of hypersensitivity to NSAIDS.

**Materials and methods**: We followed 183 adult patients (211 clinical histories) of suspected hypersensitivity to paracetamol in the Pulmonology and Allergology Centre of Vilnius University Hospital Santariskiu Klinikos. The median age of patients was 42.5 [32.75–53.25] years. Most of the patients were females 144 (78.7 %). 14 medications containing paracetamol were reported. ENDA questionnaires were completed, patch and drug provocation tests (DPTs) were performed to confirm the diagnosis.

**Results**: We adapted our clinical cases to the new classification of hypersensitivity to NSAIDs and divided our patients into five categories. 53 cases (47 patients) were nonimmunologically mediated (cross-reactive) hypersensitivity reactions to NSAIDs: four cases of “NSAIDs-exacerbated respiratory disease”, four cases of “NSAIDs-exacerbated cutaneous disease” and other 45 cases (39 patients) of “NSAIDs-induced urticaria/angioedema (NIUA)”. 86 cases (75 patients) were immunologically mediated (non-cross-reactive) hypersensitivity reactions to NSAIDs: 63 cases (55 patients) of “Single-NSAID-induced urticaria/angioedema or anaphylaxis (SNIUAA)” and 23 cases (20 patients) of “Single-NSAID-induced delayed hypersensitivity reactions (SNIRD)”. 10 cases of bronchospasm without underlying chronic airway respiratory diseases and 62 cases of anaphylaxis, maculopapular rash and other hypersensitivity reactions induced not only by paracetamol but also by other NSAIDs could not be classified according to the criteria of this classification.

Only 25 (13.7 %) patients (mostly SNIUAA (ten patients) and SNIRD (seven patients)) underwent further allergological work-up with suspected causative drug. Only seven (28 %) of them (three SNIUAA, two NIUA and two SNIRD), had true paracetamol hypersensitivity. For one patient hypersensitivity was demonstrated by patch test, and for six by DPTs. 6 out of 7 positive test results occurred as skin reactions.

**Conclusions**: True hypersensitivity to paracetamol is rare. In our research, only patients with skin reactions in their clinical history, mostly SNIUAA, were confirmed to be hypersensitive to this drug.

**Keywords**: Paracetamol; Diagnostics; Provocation tests; Drug hypersensitivity

### P54 Ibuprofen and other aryl propionic derivates can induce immediate selective hypersensitivity responses

#### Diana Perez-Alzate^1^, Natalia Blanca-López^1^, Maria Isabel Garcimartin^1^, Inmaculada Doña^2^, Maria Luisa Somoza^1^, Cristobalina Mayorga^3^, Maria Jose Torres^4^, Gador Bojas^2^, Jose Antonio Cornejo-Garcia^3^, Maria Gabriela Canto^1^, Miguel Blanca^5^

##### ^1^Allergy Unit, Infanta Leonor University Hospital, Madrid, Spain; ^2^Allergy Unit, Regional University Hospital of Malaga, Malaga, Spain; ^3^Research Laboratory and Allergy Unit, IBIMA, Regional University Hospital of Malaga, Malaga, Spain; ^4^Allergy Unit, Regional University Hospital of Malaga, Madrid, Spain; ^5^Allergy Unit, Regional University Hospital of Malaga, Malaga, Spain, Malaga, Spain

**Correspondence**: Diana Perez-Alzate

*Clinical and Translational Allergy* 2016, **6(Suppl 3)**:P54

**Background**: Arylpropionic acid derivatives are the most commonly prescribed and consumed NSAIDs and very often obtained without medical prescription. From these the most common is ibuprofen. Within the group of hypersensitivity reactions to NSAIDs, these drugs are progressively involved and include those mediated by specific immunological mechanisms and those classically classified as cross-intolerance reactions. Within the first category, IgE mediates or T cell specific responses may occur. We present a series of cases with an immediate selective response to ibuprofen and other arylpropionic derivatives confirmed by drug provocation tests (DPT). In addition to demonstrate selectivity of the response to these drugs we assessed cross-reactivity between them.

**Materials and methods**: Subjects who reported symptoms indicative of an hypersensitivity reaction to an NSAIDs were evaluated. A DPT with ASA was carried out to rule out cross-intolerance (non allergic hypersensitivity). Imputability of the different aryl propionic derivatives and assessment of cross-reactivity was carried out by drug provocation tests. Serum tryptase was quantized in peripheral blood at 2 and 24 h post episode and in the affected skin by immunohistochemistry at the moment of the appearance of the reaction.

**Results**: All subjects included had good tolerance to ASA. After proceeding with the aryl propionic acid derivative challenge, 42 cases were classified as selective immediate responders. Ibuprofen was the drug most frequently involved, followed by naproxen and desketoprofen. We found cases than only responded to one single drug and others who responded to several, confirming cross-reactivity. Quantitation of tryptase levels in peripheral blood and skin biopsies were indicative of an immediate selective response with the involvement of mast cell activation.

**Conclusions**: Ibuprofen and other aryl propionic acid derivatives can induce selective responses in subjects with NSAIDs hypersensitivity. Further studies are in progress for identifying the possible adducts (hapten-carrier complexes) implicated and the existence of cross-reactivity.

**Keywords**: Aryl-propionic derivates; Ibuprofen; Immediate selective responses

### P55 Subjects developing immediate responses to several NSAIDs can be selective with good tolerance to ASA

#### Natalia Blanca-Lopez^1^, Diana Pérez-Alzate^1^, Francisco Javier Ruano Perez^1^, Inmaculada Doña^2^, Maria Luisa Somoza^1^, Inmaculada Andreu^3^, Miguel Angel Miranda^3^, Cristobalina Mayorga^4^, Maria Jose Torres^2^, Jose Antonio Cornejo-Garcia^4^, Miguel Blanca^2^, Maria Gabriela Canto^1^

##### ^1^Allergy Unit, Infanta Leonor University Hospital, Madrid, Spain; ^2^Allergy Unit, Regional University Hospital of Malaga, Malaga, Spain; ^3^Universidad Politécnica de Valencia, Valencia, Spain; ^4^Research Laboratory and Allergy Unit, IBIMA, Regional University Hospital of Malaga, UMA, Malaga, Spain

**Correspondence**: Natalia Blanca-Lopez

*Clinical and Translational Allergy* 2016, **6(Suppl 3)**:P55

The published version of this abstract can be found at [1].

**Reference**

1. Blanca-Lopez NB, Perez-Alzate D, Dona I, Somoza ML, Mayorga C, Jose Torres M, Carnejo-Garcia JA, Blanca M, Canto G. Multuple selective responders should not be confouned with cross-intolerance to Nsaids. J Allergy Clin Immunol. 2016;137(2, Supplement):AB82.

### P56 Utility of low-dose oral aspirin challenges for diagnosis of aspirin exacerbated respiratory disease

#### Elina Jerschow^1^, Teresa Pelletier^2^, Zhen Ren^3^, Golda Hudes^4^, Marek Sanak^5^, Esperanza Morales^6^, Victor Schuster^1^, Simon D. Spivack^4^, David Rosenstreich^7^

##### ^1^Albert Einstein College of Medicine/Montefiore Medical Center, Bronx, USA; ^2^Albert Einstein College of Medicine, Bronx, USA; ^3^Jacobi Medical Center, NY, USA; ^4^Albert Einstein College of Medicine/Montefiore Medical Center, NY, USA; ^5^Jagiellonian University Medical College, Bronx, Poland; ^6^Ferkauf Graduate School of Psychology at Yeshiva University, NY, USA; ^7^Albert Einstein College of Medicine/Montefiore Medical Center, Krakow, USA

**Correspondence**: Elina Jerschow

*Clinical and Translational Allergy* 2016, **6(Suppl 3)**:P56

The published version of this abstract can be found at [1].

**Reference**

1. Jerschow E, Ren Z, Hudes G, Sanak M, Morales E, Schuster V, Spivack SD, Rosenstriech D. *Ann Allergy Asthma Immunol* 2016. 116(4): 321–8.

## Poster Walk 7: NSAID 2 (P57–P65)

### P57 Alternate regulation of cyclooxygenase-2 (COX-2) MRNA expression may predispose patients to aspirin-induced exacerbations

#### Renato Erzen, Mira Silar, Nissera Bajrovic, Matija Rijavec, Mihaela Zidarn, Peter Korosec

##### University Hospital for Respiratory Diseases and Allergy Golnik, Golnik, Slovenia

**Correspondence**: Renato Erzen

*Clinical and Translational Allergy* 2016, **6(Suppl 3)**:P57

**Background**: Exposure to acetylsalicylic acid (ASA) and other nonsteroidal anti-inflammatory drugs (NSAID) may exacerbate respiratory disease (asthma, rhinitis), cutaneous diseases (urticaria and angioedema in patients with chronic urticaria, trigger urticaria, angioedema and anaphylaxis in patients without underlying diseases.

We made a hypothesis that alternate regulation of cyclooxigenase-2 (COX-2) mRNA expression may predispose patients to ASA-induced exacerbations.

**Materials and cons**: We performed a prospective study of 40 subjects (mean age 49 years, 27 women) who had suspected hypersensitivity to ASA or other NSAID. 20 subjects had asthma (10 of them also nasal polyposis). We performed provocation tests (nasal and/or oral) with ASA in all of them. In 14 subjects tests were positive (eight nasal). Gene expression was analyzed in whole blood sample using real-time RT PCR just before ASA provocation and 4 h after provocation. We also included six Hymenoptera venom allergic patients which were followed after systemic reaction (SR) during VIT failure and four healthy control subjects.

**Results**: We found significantly important increase of COX-2 mRNA expression in patients with ASA provocation test (p = 0.004). There was no difference in ASA tolerant patients. COX-1 expression was comparable between ASA tolerant and hypersensitive patients and showed no dynamic during provocation. There were no changes in COX-2 expression in subjects with SR during VIT or in healthy control subjects.

**Conclusions**: Main findings in our study are: (1) after provocation with ASA COX-2 expression is increased in subjects with Aspirin intolerance in comparison with subjects who tolerate Aspirin; (2) during allergic reaction like SR in VIT failure we found no difference in COX-2 expression; (3) all subjects that were positive on ASA provocation test had asthma and/or nasal polyposis.

**Keywords**: Asthma/rhinitis; Hypersensitivity to acetylsalicylic acid; COX-2 MRNA expression

### P58 Anaphylaxis to diclofenac: what about the underlying mechanism?

#### Leonor Carneiro-Leão, Fabrícia Carolino, Luís Amaral, Carmen Botelho, Eunice Dias-Castro, Josefina Cernadas

##### Serviço de Imunoalergologia, Centro Hospitalar de São João, Porto, Portugal

**Correspondence**: Leonor Carneiro-Leão

*Clinical and Translational Allergy* 2016, **6(Suppl 3)**:P58

**Background**: Diclofenac is a phenylacetic acid derivative belonging to the group of arylcarboxylic acids. NSAIDs have been reported as the 2nd most frequent cause of drug-induced anaphylaxis and diclofenac was the only NSAID significantly associated with anaphylaxis. However, some doubts remain about the pathophysiology of these reactions. Here we describe a case series of anaphylaxis probably induced by diclofenac.

**Materials and methods**: Retrospective analysis of patients with suspected anaphylaxis to diclofenac studied in our DAU, who underwent skin tests (ST). Records were analyzed for clinical signs and symptoms, severity of reactions, ST and oral challenges (OC). ST were performed with commercially available IV formulation of diclofenac, in a concentration of 25 mg/ml, diluted from 1 × 10^−4^ to 1/1 for IDT and 1/1 for SPT.

**Results**: A total of 33 patients were enrolled in the final analysis. Mean age was 50(± 12.75) years, 22(68.8 %) were women, 6(18.2 %) were atopic. The time elapsed until the reaction was less than 1 h in 26(83.2 %) cases. Seventeen patients had grade 4 anaphylaxis and ten had history of re-exposure with reproducible symptoms.

Nineteen patients (62.7 %) reported reactions only to diclofenac (single-reactors) and 12 to other NSAIDs (multiple reactors) (eight to acetylsalicylic acid, two to aceclofenac). Twelve single-reactors (63.2 %) had positive ST (two with systemic symptoms during the procedure). Only four multiple reactors (33.3 %) had positive tests.

Only one diagnostic OC was performed in order to clarify a doubtful history and it was positive. OC with alternative drugs were performed in 29 patients, with meloxicam in 17 and etoricoxib in 9. Two patients had skin symptoms during the alternative OC and both were from the multiple reactors group.

**Conclusions**: We report a large series of anaphylaxis to diclofenac assessed by skin tests. As the majority of NSAIDs hypersensitivity reactions are considered to be due to a non-immunologic mechanism, non-irritative concentrations to IDT and specific IgE’s are not available. Characteristics such as the severity and time of reaction, single-reactor status and systemic symptoms during skin tests suggest IgE-dependent hypersensitivity in this study. The value of intradermal tests and their non-irritative concentrations remain to be determined doubtful history and it was positive.

**Keywords**: Diclofenac anaphylaxis mechanism

### P59 COX-2 inhibitors: are they always a safe alternative in hypersensitivity to nonsteroidal anti-inflammatory drugs?

#### Luis Amaral, Fabricia Carolino, Eunice Castro, Josefina Cernadas

##### Serviço de Imunoalergologia, Centro Hospitalar de São João E.P.E., Porto, Portugal

**Correspondence**: Luis Amaral

*Clinical and Translational Allergy* 2016, **6(Suppl 3)**:P59

**Background**: Nonsteroidal anti-inflammatory drugs (NSAID) are one of the most frequent causes of drug-induced hypersensitivity reactions worldwide. It is generally assumed that inhibition of cyclooxygenase 1 (COX-1) enzyme by the offending drugs plays a relevant pathogenic role in multiple NSAID reactors. It is generally expected tolerability to preferential/selective COX-2 inhibitors among patients with NSAIDs hypersensitivity.

**Materials and methods**: We reviewed the medical records and selected patients referred to our drug allergy unit in the past 5 years, with history of reproducible hypersensitivity reactions to NSAIDs COX-1 inhibitors and positive oral challenge (OC) with etoricoxib, a selective COX-2 inhibitor, and/or to meloxicam, a preferential COX-2 inhibitor.

**Results**: Inclusion criteria were met in 15 patients, aged 28–68 (47 ± 13 years-old), 12 females. Seven patients were atopic. Eight patients had asthma, five chronic rhinosinusitis and nasal polyposis and three patients had chronic urticaria. The primary NSAIDs hypersensitivity reactions were: eight urticaria and angioedema; four anaphylaxis and three asthma exacerbation. Eight patients presented a positive OC with meloxicam: four with immediate urticaria and angioedema; three with late-onset of angioedema and one with asthma exacerbation. One patient with late-onset angioedema to meloxicam also had a late-onset of angioedema with etoricoxib. Eight subjects had positive OC with etoricoxib: five with immediate urticarial, two with late-onset angioedema and one with anaphylactic reaction.

**Conclusions**: Based on the results of previous studies, it could be tempting to prescribe COX-2 inhibitors in cases of multiple reactors to NSAIDs COX-1 inhibitors, without establishing its tolerance in a proper setting. However, these data strongly suggest that, before prescribing an alternative NSAIDs, a provocation test should always be performed.

### P60 Management of patients with history of NSAIDs reactions prior to coronary angioplasty

#### Mona Al-Ahmad^1^, Tito Rodriguez^2^

##### ^1^Microbiology Department, Faculty of Medicine, Kuwait University, Kuwait, Kuwait; ^2^Al Rashed Allergy Center, Kuwait, Kuwait

**Correspondence**: Tito Rodriguez

*Clinical and Translational Allergy* 2016, **6(Suppl 3)**:P60

**Background**: History of NSAIDs hypersensitivity is common in some patients with coronary artery disease who are in need for coronary angioplasty. These patients require dual antiplatelet therapy to avoid in-stent thrombosis, and full evaluation of NSAIDs allergy. We present a cohort of patients with acute coronary syndrome undergoing aspirin desensitization to evaluate the short- and long-term efficacy and safety

**Materials and methods**: We developed a dynamic protocol that is based on both the patient characteristics and onset of reaction after NSAIDs. This challenge/desensitization protocol is shorter than previously published ones. The objective is to asses short and long-term efficacy and safety prior to stent placement

**Results**: A total of 19 patients with history of NSAIDs allergy were challenged with different doses of Aspirin. All patients tolerated the oral protocol at different timings of 30–45–90–120 min. The dosage given ranges between (21, 41, 81 and 162 mg) of aspirin given by mouth after premedication with montelukast. We had two patients reacting during the procedure and one during a 6 h-follow-up. All reactions were limited to the skin. All patients tolerated the required dose of aspirin within 60–150 min. Those requiring urgent catheterization were desensitized within 90 min.

**Conclusions**: Our protocol provide an effective and safe short and long-time administration of Aspirin 81 mg dose in patients with history of NSAIDs allergy

**Keywords**: Aspirin; Desensitization; Coronary

### P61 Oral drug challenge with non-steroidal anti-inflammatory drug under spirometric control: clinical series of 110 patients

#### João Pedro Azevedo, Emília Faria, Beatriz Tavares, Frederico Regateiro, Ana Todo-Bom

##### Centro Hospitalar e Universitário de Coimbra, Coimbra, Portugal

**Correspondence**: João Pedro Azevedo

*Clinical and Translational Allergy* 2016, **6(Suppl 3)**:P61

**Background**: Oral drug challenge (ODC) is the gold standard for the diagnosis of hypersensitivity reactions to non-steroidal anti-inflammatory (NSAIDs). If suspicion of respiratory symptoms, the ODC might include a lung function evaluation (LFE). Aim: To evaluate the lung function volumes during ODC to NSAIDs performed in our department between 2010 and 2015.

**Materials and methods**: One-hundred and ten ODCs with NSAIDs under LFE were performed. An alternative drug was tested in 77 of the patients (70 %) whereas 33 were diagnostic tests (30 %). The drugs tested were meloxicam (n = 46, 41.8 %); acetylsalicylic acid (19, 17.4 %); etoricoxib (16, 14.6 %); nimesulide (15, 13.6 %); ibuprofen (4, 3.6 %); celecoxib (3, 2.7 %); metamizol (3, 2.7 %); acetaminophen (3, 2.7 %) and diclofenac (1, 0.9 %). The criteria for a positive ODC were: a decrease in FEV1 ≥ 20 % of baseline, a decrease of MMEF75/25 ≥ 25 % of baseline or when major symptoms occurred. Statistical analysis based on independent sample t test and paired t test.

**Results**: Seventy-nine patients were female (71.8 %) (44.8 ± 15.6 years) and 31 were male (28.2 %) (40.5 ± 16.4 years). Sixty-two patients had a previous diagnosis of asthma (56.4 %), 18 of which with diagnosis of NSAID-exacerbated respiratory disease (NERD) (16.4 %).

ODC was positive in seven cases (four by decrease of volumes and three by reported symptoms): five alternatives and two diagnostic (four with meloxicam; one ibuprofen, one metamizol; one etoricoxib), two patients with no previous asthma diagnosis.

Taking into consideration the whole population tested, we observed statistically significant decreases in FEV1 and FVC values at 30 and 120 min after medication versus baseline; and also a decrease in volumes at 30, 60, 120, 180 and 240 min after the second dose *vs* baseline (p < 0.05).

When comparing alternative versus diagnostic ODCs, we found statistically significant differences in the measurement of FEV1 at 1 h after the second dose (p < 0.05).

**Conclusions**: The number of positive ODCs was low (6.36 %). Patients with asthma have an increased risk of reaction even with alternative NSAIDs. There was a statistically significant decrease in FEV1 at almost all timepoints after NSAIDs compared to baseline, even including negative challenges in the analysis. This suggests that NSAIDs might have a general effect to reduce FEV1.

**Keywords**: NSAID; NSAID-exacerbated respiratory disease; Asthma

### P62 Prevalence and incidence of analgesic hypersensitivity reactions in Colombia

#### Pablo Andrés Miranda^1^, Bautista De La Cruz Hoyos^2^

##### ^1^Universidad Nacional de Colombia, Cartagena, Colombia; ^2^Centro de Especialistas Santo Domingo - Alergologia, Cartagena, Colombia

**Correspondence**: Pablo Andrés Miranda

*Clinical and Translational Allergy* 2016, **6(Suppl 3)**:P62

**Background**: The most common drug hypersensitivity reactions (DHR) involve analgesic such as aspirin and other non steroidal anti-inflammatory drugs.

**Materials and methods**: Records with personal history of allergy analgesics diagnosis (ICD-10 code Z886) of Information System of Social Protection (SISPRO) between 2010 and 2014 were included. To determine the prevalence and incidence of AHR, population estimates from the National Statistics Department of Colombia (DANE) were used.

**Results**: 1657 cases with personal history of AHR between 2010 and 2015 were identified. 223 cases were confirmed news diagnoses in the same period. On average 331 cases of AHR per year were estimated. The estimated annual prevalence AHR were seven cases per million (2010 = 6; 2011 = 7.1; 2012 = 8.1; 2013 = 6.8 and 2014 = 7.2). The estimated annual incidence AHR were 0.9 cases per million (2010 = 0.8; 2011 = 1.1; 2012 = 0.9; 2013 = 0.8 and 2014 = 1). Most cases range between 19 and 59 years age (Table 1).

**Table 1:** Prevalence and incidence AHR Colombia 2010–2014

	2010 % (n)	2011 % (n)	2012 % (n)	2013 % (n)	2014 % (n)
*Prevalence AHR Colombia* Cases per million					
1–5 años	0.4 (1)	5.1 (17)	2.4 (9)	5.2 (17)	3.8 (13)
6–9 años	5.4 (15)	6.9 (23)	4 (15)	5.2 (17)	3.2 (11)
10–14 años	8 (22)	6.3 (21)	9.2 (35)	10.8 (35)	12.1 (42)
15–18 años	8.3 (23)	6.9 (23)	7.4 (28)	7.7 (25)	10.8 (37)
19–26 años	14.9 (41)	13.9 (46)	19.8 (75)	18.8 (61)	16.8 (58)
27–44 años	34.8 (96)	32 (106)	29.8 (113)	26.2 (85)	27.7 (96)
45–59 años	19.2 (53)	19 (63)	16.4 (62)	16.6 (54)	14.5 (50)
>60 años	9.1 (25)	9.7 (32)	11.1 (42)	9.5 (31)	11.3 (39)
Total	100 (276)	100 (331)	100 (379)	100 (325)	100 (346)
*Incidence AHR Colombia* Cases per million					
1–5 años	0 (0)	0 (0)	0 (0)	2.5 (1)	8.2 (4)
6–9 años	5.4 (3)	0 (0)	2.4 (1)	5 (2)	10.2 (5)
10–14 años	8 (4)	9.3 (5)	9.5 (4)	2.5 (1)	10.2 (5)
15–18 años	8.3 (2)	7.4 (4)	7.1 (3)	10 (4)	18.4 (9)
19–26 años	14.9 (4)	20.4 (11)	21.4 (9)	22.5 (9)	20.4 (10)
27–44 años	34.8 (10)	35.2 (19)	26.2 (11)	25 (10)	16.3 (8)
45–59 años	19.2 (8)	18.5 (10)	23.8 (10)	22.5 (9)	8.2 (4)
>60 años	9.1 (7)	9.3 (5)	9.5 (4)	10 (4)	8.2 (4)
Total	100 (38)	100 (54)	100 (42)	100 (40)	100 (49)

**Conclusions**: Both under-diagnosis and over-diagnosis AHR are common in the world. Population studies with confirmatory tests AHR in Colombia are required.

**Keywords**: Analgesic; Allergy; Hipersensitivity

### P63 Recent endoscopic sinus surgery lessens reactions during aspirin challenge in patients with aspirin exacerbated respiratory disease

#### Teresa Pelletier^1^, Waleed Abuzeid^2^, Nadeem Akbar^2^, Marc Gibber^2^, Marvin Fried^2^, Weiguo Han^1^, Taha Keskin^2^, Robert Tamayev^2^, Golda Hudes^2^, Simon D. Spivack^2^, David Rosenstreich^2^, Elina Jerschow^2^

##### ^1^Albert Einstein College of Medicine, Bronx, USA; ^2^Albert Einstein College of Medicine/Montefiore Medical Center, Bronx, USA

**Correspondence**: Elina Jerschow

*Clinical and Translational Allergy* 2016, **6(Suppl 3)**:P63

**Background**: Aspirin-exacerbated respiratory disease (AERD) is diagnosed clinically through a positive oral graded aspirin challenge. Upon confirming aspirin hypersensitivity, aspirin desensitization and treatment has been proven to reduce recurrence of nasal polyposis and asthma symptoms. However, negative aspirin challenges have been reported to occur in otherwise aspirin allergic individuals (“silent challenges”) after intake of antihistamines, leukotriene blockers, or oral corticosteroids. We sought to determine whether recent endoscopic sinus surgery (ESS) also affects reactions to aspirin challenge in patients with AERD.

**Materials and methods**: 19 AERD patients underwent two aspirin challenges: one before and the other after (within 8 weeks) their most recent ESS. Reactions to aspirin were evaluated by changes in forced expiratory volume in 1 s (FEVl, percent predicted), nasal peak flow (NPF), and fraction of exhaled nitric oxide (FeNO).

**Results**: Decreased time between ESS and challenge was associated with decreased frequency of positive reactions: all 19 patients with a history averaging 32 months (IQR 9–72) since last ESS, reacted to aspirin. In contrast, only 10 (52.6 %) of these patients, challenged on average of 1.1 months (IQR 0.8–1.4) after ESS, reacted to aspirin. Nine patients (47.4 %) had no clinically apparent reaction to aspirin challenge after recent ESS. In the 10 patients who had a positive challenge after recent ESS, the decrease in FEV1 during this challenge was less than during the baseline challenge before ESS: −8.9 ± 1.2 vs. −19.7 ± 3.7 % (*p* < 0.01), respectively. NPF decrease was also smaller during the challenge after recent ESS than before recent ESS, −11.1 ± 5.1 vs. −25.5 ± 6.2 % (p < 0.01), respectively. FeNO significantly decreased in all patients during the first challenge (−22.2 % (IQR −50.6 to −20.9)) and decreased only in those who had positive challenges after recent ESS (−32.5 % (IQR −35.1 to −22.6; *p* = 0.3).

**Conclusions**: Recent ESS could contribute to a false-negative aspirin challenge. Patients who reacted to aspirin during the challenge after recent ESS had milder reactions comparing to the challenge before ESS. This presents a clinical dilemma: while appearing safer, aspirin challenges after a recent ESS may have a decreased diagnostic sensitivity so that some AERD patients could be misclassified as aspirin-tolerant. Clinicians should consider the possibility of false-negative challenges after recent ESS.

**Keywords**: Aspirin exacerbated respiratory disease; Oral graded aspirin challenge; Endoscopic sinus surgery; Silent challenge

### P64 Safe use of imidazole salycilate in a case of multiple NSAIDs induced urticaria-angioedema

#### Elisa Boni, Marina Russello, Marina Mauro

##### Hospital Sant’Anna, Como, Italy

**Correspondence**: Elisa Boni

*Clinical and Translational Allergy* 2016, **6(Suppl 3)**:P64

**Background**: Hypersensitivity to non-steroidal anti-inflammatory drugs (NSAIDs) has been classified by Ayuso et al. into different phenotypes. Multiple NSAID-induced urticaria-angioedema (MNSAID-UA) is presumably related to cyclo-oxygenase 1 (COX -1) inhibition. Patients with MNSAID-UA have reactions to multiple chemically unrelated molecules while usually they tolerate low COX-1 inhibitors.

**Report**: We report the case of a 32 years old man who experienced several episodes of angioedema of lips and face after intake of acetylsalicylic acid (ASA), in one occasion associated to the intake of paracetamol. Diagnosis of MNSAID-UA was confirmed by oral drug provocation test (DPT) to ibuprofen, a strong COX-1 inhibitor chemically unrelated to ASA, that induced labial angioedema. DPT with alternative drugs, defined as low COX-1 inhibitors, was then performed. Angioedema also appeared with nimesulide and paracetamol. Instead, the patient tolerated imidazole salicylate.

**How this report contributes to current knowledge**: Low COX-1 inhibitors as paracetamol and nimesulide not always are tolerated by patients with MNSAID-UA. This report confirms the safe use of imidazol salicylate in patients with hypersensitivity to ASA presumably due to non-interference with the cyclo-oxygenase pathway.

**Consent**: Written informed consent was obtained from the patient for publication of this abstract and any accompanying images.

### P65 Selective hypersensitivity reactions to ibuprofen—seven years experience

#### Marta Ferreira Neto

##### CHLN-HSM, Lisbon, Portugal

**Correspondence**: Marta Ferreira Neto

*Clinical and Translational Allergy* 2016, **6(Suppl 3)**:P65

**Background**: Immunologically mediated (non cross-reactive) hypersensitivity reactions to nonsteroidal anti-inflammatory drugs (NSAIDs) are classified in two groups: Single NSAID-induced urticaria/angioedema or anaphylaxis and Single NSAID-induced delayed hypersensitivity reactions. In these entities patients have hypersensitivity to NSAIDS belonging to the same chemical group and tolerate chemically nonrelated ones. Usually there isn’t an underlying chronic disease like asthma or urticaria. Ibuprofen (Ib), belonging to Propionic acid derivatives (Pad) group, is one of the most frequently used NSAIDs, with frequent hypersensitivity drug reactions reports.

**Materials and methods**: We retrospectively analysed all patients (Pt) referred to our drug outpatient visit, from January 2008 to December 2015, with a selective history of hypersensitivity reaction to Ib. Oral drug challenges (ODC) were performed, in order to establish the diagnosis.

**Results**: A total of 10 Pt, aged between 18 and 79, 90 % female were included. After intake of Ib, 10(100 %) Pt reported cutaneous symptoms: 2(20 %) Urticaria (U) and 8(80 %) U/Angioedema. Besides Ib, Flurbiprofen, Dexcetoprofen and Naproxen were also the culprit drugs in three different Pt. All 10 Pt tolerated Acetaminophen after the reaction. Hypersensitivity reactions were immediate in 3(30 %) Pt (<1 h), late (1–6 h) in 1(10 %) and accelerated (8–24 h) in 6(60 %). ODC were performed with Ibuprofen, Acetylsalicylic acid (ASA) and Nimesulide (N) in 6(60 %) patients, and in the remaining 4(40 %) only with ASA and N. All ODC (100 %) with Ib were positive, with reproductible symptoms, even in the non immediate reactions. All Pt (100 %) had negative ODC with ASA and N.

**Conclusions**: Single NSAID-induced urticaria/angioedema to Ib was established in 10 (100 %) Pt and to chemically related compounds of Pad group in three (30 %) Pt. Women represent the majority, cutaneous symptoms are the only clinical presentation and 70 % are non-immediate reactions. Other strong Cox1 inhibitors (except Pad group), Nimesulida and Acetaminophen are the alternatives in the 10 patients.

**Keywords**: Selective; Ibuprofen; Oral challenges

## Poster Walk 8: Epidemiological methods (P66–P72)

### P66 Allopurinol hypersensitivity: a 7-year review

#### Lise Brosseron, Daniela Malheiro, Susana Cadinha, Patrícia Barreira, J. P. Moreira Da Silva

##### Centro Hospitalar Vila Nova de Gaia/Espinho, EPE, Vila Nova De Gaia, Portugal

**Correspondence**: Lise Brosseron

*Clinical and Translational Allergy* 2016, **6(Suppl 3)**:P66

**Background**: Allopurinol, an antihyperuricaemic agent, is used as first-line treatment of chronic gout. Allopurinol hypersensitivity (AH) is a rare but important cause of hypersensitivity reactions, ranging from mild cutaneous manifestations to life-threatening severe cutaneous adverse reactions (SCAR). Despite several risk factors have been proposed, the underlying mechanisms remain unknown. Our aim was to characterize a series of patients with suspected AH referred to our drug allergy department during a 7-year period (2009–2015).

**Materials and methods**: A retrospective analysis was performed, assessing demographic and clinical data. AH was confirmed by a positive drug provocation test (DPT), positive lymphocyte transformation test (LTT) or reaction upon desensitization, and considered probable based only on a suggestive clinical history.

**Results**: Among a total of 954 patients, 29 (3 %) had suspected AH. The mean age was 69 ± 10 years and 16 (55 %) were male. Allopurinol was prescribed for gout in 14 (48 %), asymptomatic hyperuricemia in 12 (41 %) and malignancies in 3 (10 %) patients; 20 (69 %) were polymedicated (≥4 drugs). Cutaneous reactions were reported by 27 (93 %) subjects (14 exanthema, 5 urticaria/angioedema, 2 fixed drug eruption, 3 vasculitis and 3 DRESS). Twenty-six (90 %) reported delayed reactions (DR), 1 reported immediate reaction and 2 were unable to recall onset. SPT (3) and patch tests (25) were negative in all patients tested. LTT performed in 12 patients (five exanthema, one urticaria/angioedema, three vasculitis and three DRESS) was positive in four, negative in five, doubtful in two and undetermined in one. DPT was positive in 2 out of 11 and long term challenge in 2 out of 8. Four patients were submitted to desensitization: three developed reaction during the procedure, confirming diagnosis; two were able to tolerate treatment. AH was confirmed in 10 (35 %), considered probable in six (21 %), excluded in seven (24 %) and inconclusive in six patients. Among confirmed AH patients, all were DR and 80 % had started Allopurinol recently (≤10 days).

**Conclusions**: As previously described in the literature, our study suggests that AH is rare, usually presents with delayed cutaneous symptoms and can be related to recent Background of Allopurinol. In the diagnostic workup of suspected AH, DPT remains the gold-standard while patch tests appear to be unhelpful. We discuss the usefulness of TTL, which seems promising, especially in SCAR where DPT is contraindicated.

### P67 Antibiotic allergy labelling is associated with increased hospital readmission rates in Australia

#### Brittany Knezevic^1^, Dustin Sprigg^1^, Michelle Trevenen^2^, Jason Seet^1^, Jason Trubiano^3^, William Smith^4^, Yogesh Jeelall^2^, Sandra Vale^5^, Richard Loh^6^, Andrew Mclean-Tooke^7^, Michaela Lucas^7^

##### ^1^Sir Charles Gairdner Hospital, Perth, Australia; ^2^The University of Western Australia, Perth, Australia; ^3^Austin Health, Melbourne, Australia; ^4^Royal Adelaide Hospital, Adelaide, Australia; ^5^Australian Society of Clinical Immunology and Allergy, Balgowlah, Australia; ^6^Princess Margaret Hospital, Perth, Australia; ^7^Pathwest Laboratory Medicine, Queen Elizabeth II Medical Centre, Perth, Australia

**Correspondence**: Michaela Lucas

*Clinical and Translational Allergy* 2016, **6(Suppl 3)**:P67

**Background**: Patients frequently report antibiotic allergies, however only about 10 % of labelled patients have a true allergy. This study investigates the documentation of antibiotic “allergy” labels (AALs) and the effect of labelling on clinical outcomes in an adult tertiary hospital in Australia.

**Materials and methods**: We performed a retrospective single-centre cross-sectional analysis of 737 inpatients in a major teaching hospital in Western Australia. All patients were captured in the 2013 and 2014 National Antimicrobial Prescribing Surveys (NAPS). NAPS is an annual audit of Australian health services, led by The Australian Commission on Safety and Quality in Health Care, to assess volume and appropriateness of antimicrobial prescribing. Data collected by detailed chart review, included antibiotic adverse drug reactions (ADRs), antibiotic cost, prescribing appropriateness, prevalence of multi-drug resistant organisms, length of stay, intensive care admission and readmissions.

**Results**: Complete data were captured for 687 patients. 278 (40 %) were aged 70 or above, 322 (47 %) were female and 279 (41 %) were prescribed antibiotics at the time of audit. AALs were recorded in 122 (18 %) of all patients. The majority of AALs were penicillin labels (n = 87; 71 %). Details of the clinical reactions to the culprit antibiotic were documented for 80 of 141 (57 %) individual allergy labels: 61 described symptoms consistent with drug allergies and 19 were non-specific symptoms. Five patients were receiving an antibiotic that would be considered contraindicated according to their documented allergy status. Females and older patients were significantly more likely to have an AAL (gender: OR 2.54, 95 % CI = 1.69–3.82, p < 0.001; for a one standard deviation (19.6 years) increase in age: OR 1.31, 95 % CI = 1.06–1.60, p = 0.007). Patients with AALs had significantly more hospital readmissions within 4 weeks (p = 0.001) and 6 months (p = 0.025) of discharge, compared with unlabelled patients independent of age and gender. The majority of patients with AALs (84 %) who were readmitted had a diagnosis of an infection.

**Conclusions**: This first Australian study shows that purported AALs are common but poorly documented in hospital records. Patients with AALs are significantly more likely to require readmissions. There may be a role for antibiotic allergy delabelling to mitigate the clinical and associated economic burdens for patients with invalid allergy labels.

**Keywords**: Antibiotic; Allergy; Adverse drug reactions; Delabelling

### P68 Experts’ opinions on severe cutaneous adverse drug reactions-report of a survey from the 9th international congress on cutaneous adverse drug reactions 2015

#### Roni P. Dodiuk-Gad^1^, Cristina Olteanu^2^, Wen-Hung Chung^3^, Neil H. Shear^4^

##### ^1^Department of Dermatology, Ha’emek Medical Center, Afula, Israel; ^2^Faculty of Medicine, University of Toronto, Toronto, Canada; ^3^Department of Dermatology, Drug Hypersensitivity Clinical and Research Center, Chang Gung Memorial Hospitals, Taipei, Taiwan; ^4^Division of Dermatology, Department of Medicine, Sunnybrook Health Sciences Centre, Linkou, Canada

**Correspondence**: Cristina Olteanu

*Clinical and Translational Allergy* 2016, **6(Suppl 3)**:P68

**Background**: On June 8th, 2015, the 9th International Congress on Cutaneous Adverse Drug Reactions (cADR) was held at the 23rd World Congress of Dermatology. Eighty participants attended the congress from 23 countries; a variety of specialists with different areas of expertise included dermatology, regulatory agencies and public health, clinical pharmacology, immunology, infectious disease, oncology, pediatrics, etc. During the Congress, surveys were conducted to learn about the participants’ experiences and perspectives regarding four major topics: pharmacogenomics and severe cutaneous adverse drug reactions (SCAR), drug reaction with eosinophilia and systemic symptoms (DRESS)/drug-induced hypersensitivity syndrome (DIHS), Stevens–Johnson syndrome and toxic epidermal necrolysis (SJS/TEN), and acute generalized exanthematous pustulosis (AGEP).

**Materials and methods**: Participants answered survey questions anonymously through an electronic voting system.

**Results**: In a survey of the pharmacogenomics of SCAR, 21 % of participants regularly conducted genetic screening prior to treatment with carbamazepine (N = 53) and 15 % of participants regularly conducted genetic screening prior to administration with allopurinol (N = 52). Seventy-eight percent reported that there is a lack of knowledge among physicians regarding genetic screening for Human Leukocyte Antigens (HLAs) for preventing SCAR in their country (N = 51). In a survey of SJS/TEN, systemic corticosteroids was the preferred treatment in 45 % followed by only supportive treatment in 18 %, intravenous immunoglobulin in 16 %, and cyclosporine in 14 % (N = 51). In a survey of DRESS/DIHS, 36 % regularly assess reactivation of human herpesvirus-6 (HHV-6) in their management of patients with DRESS/DIHS (N = 53), and 33 % monitor late autoimmune complications in these patients (N = 54). In a survey of AGEP, 67 % of participants always conduct assessment for systemic involvement (N = 47).

**Conclusions**: There is a lack of implementation of regulatory agencies recommendations’ regarding genetic screening prior to treatment with carbamazepine or allopurinol. There is still no international consensus for the management of patients with SJS/TEN. Assessing reactivation of HHV-6 and monitoring late autoimmune complications in patients with DRESS/DIHS is not commonly done. Assessing systemic involvement in patients with AGEP was found to be commonly conducted.

**Keywords**: SJS/TEN; DRESS; AGEP

### P69 HLA-A*31-positive AGEP with carbamazepine use and other severe cutaneous adverse drug reactions (SCARs) detected by electronic medical records screening

#### Sabine Müller^1^, Ursula Amstutz^2^, Lukas Jörg^3^, Nikhil Yawalkar^4^, Stephan Krähenbühl^1^

##### ^1^Department of Clinical Pharmacology and Regional Pharmacovigilance Center, Inselspital, University Hospital Bern, Bern, Switzerland; ^2^University Institute of Clinical Chemistry, Inselspital, University Hospital Bern and University of Bern, Bern, Switzerland; ^3^Department of Rheumatology, Clinical Immunology and Allergology, Inselspital, University Hospital Bern, Bern, Switzerland; ^4^Department of Dermatology, Inselspital, University Hospital Bern, Bern, Switzerland

**Correspondence**: Sabine Müller

*Clinical and Translational Allergy* 2016, **6(Suppl 3)**:P69

**Background**: Pharmacovigilance (PV) programs based on spontaneous reporting of adverse drug reactions (ADRs) are compromised by underreporting and inconstant data quality. Underreporting of severe adverse drug reactions by hospitals is in the range of 95 % (Drug Saf 2006;29:385–96). This study aims to assess underreporting and to evaluate the potential of electronic chart screening for SCARs in two departments (dermatology and allergology) as a proactive case detection method regarding detection rate and data quality.

**Materials and methods**: Electronic reports from January 2014 to December 2015 of the allergology and dermatology units at the Inselspital Bern (Switzerland) were reviewed monthly for diagnosis of SCARs (AGEP, SJS/TEN, DRESS) and compared to SCARs reported to the PV service of the clinical pharmacology unit. Cases were evaluated for standard PV parameters and results of allergologic and HLA testing if available.

**Results**: During this 2-year period, 27 SCARs were registered at our PV unit. 2 SCAR cases were reported spontaneously (one DRESS, one SJS), while 25 SCARs were detected proactively (13 AGEP, 9 DRESS, 3 SJS). Interestingly, among the screened charts, a patient with carbamazepine-induced AGEP was a carrier for HLA-A*31 (HLA-A*3101 to be confirmed), possibly associated with carbamazepine-induced AGEP (Epilepsia 2014;55:496–506).

**Conclusions**: Spontaneous reporting is insufficient, even for severe ADRs with well-established causality. Combination of chart screening with standard PV programs is a promising method to enhance detection rate and signal quality for rare idiosyncratic ADRs.

### P70 Patients with suspected drug allergy: a specific psychological profile?

#### Eunice Dias-Castro^1^, Ana Leblanc^1^, Laura Ribeiro^2^, Josefina R. Cernadas^1^

##### ^1^Allergy and Clinical Immunology Department, Centro Hospitalar S. João EPE, Porto, Portugal; ^2^Biochemistry Department, Medical Education and Simulation Department, Faculty of Medicine, University of Porto, Porto, Portugal

**Correspondence**: Eunice Dias-Castro

*Clinical and Translational Allergy* 2016, **6(Suppl 3)**:P70

**Background**: There are several studies demonstrating an important association between allergic diseases and the psychological characteristics of the patients. The majority involves skin or respiratory diseases and only a few drug allergy (DA). Patients with adverse reactions to drugs appear to have more psychological disturbances than those with asthma/rhinitis.

**Materials and methods**: We evaluated psychological characteristics of 115 consecutively patients >16 years-old, studied in our Department for suspected DA. Four self-completed validated questionnaires (anxiety, depression, alexithymia, personality type) were used. Evaluation of patient emotions at the time of the reaction was also performed based on a numerical scale to quantify fear/panic and an open question. The control group included 55 patients from the outpatient clinic without any history of DA. Description of variables was done through absolute and relative frequencies. Chi square and Fisher test were used to evaluate the association between variables and groups. The magnitude of the associations was measured by the Odds Ratios. The significance of the difference between the medium intensity of fear/panic at the time of the reaction by gender was evaluated by a student t-test for independent samples. All the variables with a proof value <=0.20 in the univariate analysis were considered for the multivariate analysis. The level of significance was considered 5 %. Statistical analysis used the SPSS, version 20.0.

**Results**: There were no significant differences between the two groups concerning demographic or social characteristics, except for age (p = 0.025). Allergic diseases were significantly more prevalent in the control group (51.1 vs. 87.3 %; p < 0.001) but other diseases were significantly higher in the patient group (67.5 vs. 27.8 %; p < 0.001). The majority of the controls were on regular medication (68.4 vs. 94.2 %; p < 0.001), although psychiatric medication was more frequently used in the case group (32.0 vs. 5.8 %; p < 0.001). After adjustment for age, allergic disease, other diseases and daily medication, the suspicion of DA did not show any association with psychological characteristics. The differences between the medium intensity of fear/panic at the time of the reaction by gender were statistically significant (p = 0.022), with males having a lower result (2.7 points vs. 3.6).

**Conclusions**: The suspicion of DA did not show any association with psychological characteristics of the patients. The analysis of those with confirmed DA is still ongoing.

### P71 Use of an electronic device and a computerized mathematic algorithm to detect the allergic drug reactions through the analysis of heart rate variability

#### Arantza Vega^1^, Raquel Gutierrez Rivas^2^, Ana Alonso^1^, Juan Maria Beitia^1^, Belén Mateo^1^, Remedios Cárdenas^1^, Juan Jesus Garcia-Dominguez^2^

##### ^1^Hospital Universitario de Guadalajara, Guadalajara, Spain; ^2^Universidad de Alcalá, Alcalá De Henares, Spain

**Correspondence**: Arantza Vega

*Clinical and Translational Allergy* 2016, **6(Suppl 3)**:P71

**Background**: Challenge test (CT) is the gold-standard for the diagnosis of drug allergy. Its implementation involves a high consumption of time and resources and implies the risk of severe adverse reactions apparence.

Currently there is not a diagnostic tool able to perform an early detection of the allergic reactions, thus reducing the length of the challenge test and decreasing the onset of severe allergic reactions.

Heart rate variability has been used for help to the diagnosis of several illnesses. It has been recently used for the detection of allergic reactions. We explore the use of a heart rate variability monitoring device to detect in real time the allergic reactions in patients undergoing a CT.

**Materials and methods**: Patients, older than 12 years, with suspected drug allergy that, following the normal diagnostic protocol, were undergoing a CT and agreed to participate. A remote monitoring device called Shimmer was placed from 10 min before the star of the test until Its completion. Increasing doses of the drug were administered at 30 or 60 min interval until the end of the test or until the onset of allergic symptoms. Heart rate variability was analysed by the proposed mathematic algorithm, and Its results were compared to the CT results.

**Results**: One hundred and twenty thee patients were studied: 81 women, 42 men, mean age 41.8 years (14–92). Drugs tested included NSAID (50), Betalactamics (40), other antibiotics (8) and others (25). The drug CT was positive in seven of them (5.6 %).

The algorithm detection showed a positive result in nineteen patients and a negative one in 104. Its sensitivity and specificity were 57 and 87 % respectively. When NSAIDs CT were ruled out the results improved reaching 100 % sensitivity and 96 % specificity. The global negative predictive value was 97 %.

The detection took place at least 90 min before the onset of symptoms.

**Conclusions**: The use of a heart rate variability device with a mathematic algorithm can be a useful tool in drug allergy diagnosis. Further studies are needed.

**Keywords**: Challenge test; Heart rate variability; Electronic device

### P72 Variation in ERAP influences risk for HLA-B*57:01 positive abacavir hypersensitivity

#### Rebecca Pavlos^1^, Kaija Strautins^1^, Ian James^1^, Simon Mallal^2^, Alec Redwood^1^, Elizabeth Phillips^2^

##### ^1^Murdoch University, Perth, Australia; ^2^Vanderbilt University, Nashville, USA

**Correspondence**: Rebecca Pavlos

*Clinical and Translational Allergy* 2016, **6(Suppl 3)**:P72

**Background**: Abacavir (ABC) binds non-covalently to the floor of the peptide-binding groove of HLA-B*57:01, altering the chemistry and shape of the antigen binding cleft. This allows previously untolerized self-peptides to be presented by HLA-B*57:01 to T cells. Endoplasmic reticulum aminopeptidases (ERAPs) trim peptides for MHC Class I presentation, influencing the degree and specificity of the CD8+ T-cell response. Genetic variation within ERAP adds to the positive predictive value (PPV) of the HLA class I risk allele in autoimmune diseases such as HLA-B27 positive ankylosing spondylitis. Considering the altered peptide repertoire mechanism of ABC HSR we hypothesize that variation in ERAP may help explain why 45 % of patients carrying HLA-B*57:01 can tolerate ABC.

**Materials and methods**: 3′UTR, intron and exon encoded SNPs which characterise gene haplotypes were examined for ERAP1 in HLA-B*57:01 + ABC patch test positive (PT +) patients [n = 53] and HLA-B*57:01 + ABC tolerant controls [n = 22] with sequence-based typing. Rs2248374, a tag SNP for functional ERAP2 haplotypes was also examined. Haplotype A is tagged by the (A) allele, while haplotype B is tagged by rs2248374(G). Fisher exact tests and multiple logistic regressions were used to compare genotypes between ABC HSR PT+ and tolerant groups.

**Results**: HLA-B*57:01 + ABC tolerance was associated with rs27434(GG) (18/22(82 %) vs 24/53(45 %) in ABC HSR PT+, p = 0.005). This SNP maps to the active site within ERAP1 (AA356). For an HLA-B*57:01 positive population, with 45 % of cases tolerant to abacavir, the estimated chances of tolerance given rs27434 genotype are AA (0 %), AG (13 %) and GG (43 %). A missense mutation within the domain junction (rs30187(C)) important in conformational change of ERAP1 (AA528), was overrepresented in HLA-B*57:01 + ABC tolerant individuals (p = 0.04). Analysis indicated linkage between rs27044 and rs30187, rs17482078 and rs2287987, and between rs30187 and rs27434 (all p < 0.0001). In a multivariable model with rs27434(GG), the ERAP2 SNP (rs2248374(G)) that tags haplotype B, characterized by a truncated protein, was decreased in tolerant individuals (p = 0.005).

**Conclusions**: ERAP variants are important in the development of ABC HSR. ERAP activity may influence the repertoire of peptides presented by HLA-B*57:01 or influence early changes in immunodominant epitope selection. This provides a potential pathogenic mechanism for the development of ABC HSR or ABC tolerance in HLA-B*57:01 carriers.

**Keywords**: ERAP; Abacavir; HLA-B*57:01

## Poster Walk 9: DRESS/AGEP (P73–P81)

### P73 A clinical case of DRESS syndrome in a child after administration of amoxicillin-clavulanic acid

#### Rita Aguiar^1^, Anabela Lopes^1^, Ana Neves^2^, Maria Do Céu Machado^2^, M. A. Pereira-Barbosa^1^

##### ^1^Immunoallergology Department, Hospital de Santa Maria-Centro Hospitalar Lisboa Norte, Lisbon, Portugal; ^2^Pediatrics Department, Hospital de Santa Maria-Centro Hospitalar Lisboa Norte, Lisbon, Portugal

**Correspondence**: Rita Aguiar

*Clinical and Translational Allergy* 2016, **6(Suppl 3)**:P73

**Background**: Drug rash with eosinophilia and systemic symptoms (DRESS) syndrome is an uncommon but serious hypersensitivity drug reaction. It is characterized by a polymorphic disseminated eruption with fever and multiple organ dysfunction. DRESS syndrome is a rare entity in children.

The authors describe a case of a children admitted to our hospital for amoxicillin-clavulanate induced DRESS syndrome.

**Materials and methods**: **case report**: Male, 31 months-old, presented to the emergency department with diffuse erythema involving trunk and extremities, sparing palms of hands and feet, angioedema of the tongue and fever after treatment with amoxicillin/clavulanate for cutaneous infection.

Laboratory findings revealed lymphocytosis, eosinophilia (600/µl), and elevated serum transaminases AST 139 U/l, ALT 440 U/l, LDH 976 U/l. No previous drug allergies were reported at the time of presentation.

On the second day of hospitalization, the patient’s rash was still persistent ant it appeared petechiae and hemorrhagic suffusion suggestive of vasculitis on the face, forehead and periorbital without worsening throughout the hospitalization.

Infectious markers and serological tests were negative. Corticosteroid treatment was started after exclusion of other potentially serious conditions including infections and hematologic malignancies. The results of the skin biopsy taken from the lesions revealed superficial perivascular dermatitis involving spongiotic eosinophils compatible with spongiotic drug eruption. A dramatic improvement was observed and his clinical and laboratory findings were recovered on the 5th day of the treatment.

The high fever, lymphocytosis and eosinophilia were resolved (eosinophilia rate was 1.8 %). ALT 114, AST 57, without changes of cholestasis (GGT 28, LDH 567, total bilirubin 0.2 mg/dl). Abdominal ultrasound unchanged.

Specific IgE β-lactams were negative.

Patch tests with β-lactams revealed positive reaction to amoxicillin.

**Conclusions**: Given the significant morbidity early recognition of drug reaction with eosinophilia and systemic symptoms syndrome and initiation of appropriate therapy are imperative in limiting morbidity.

In this study, patch testing was a safe and useful method in confirming the culprit drug in DRESS induced β-lactam. The pathogenesis of DRESS is not yet entirely clarified, but positive patch tests suggest a drug-dependent delayed hypersensitivity mechanism.

**Keywords**: DRESS Syndrome; Amoxicillin-clavulanate; Patch testing

**Consent**: Written informed consent was obtained from the patient for publication of this abstract and any accompanying images.

### P74 Acute generalized exanthematous pustulosis (AGEP) induced by mesalazine, reliable and oftenly used drug to treat inflammatory bowel disease

#### Ceyda Tunakan Dalgiç, Emine Nihal Mete Gökmen, Fatma Düsünür Günsen, Gökten Bulut, Fatma Ömür Ardeniz, Okan Gülbahar, Ali Kokuludag, Aytül Zerrin Sin

##### Ege University Medical Faculty, Department of Allergy and Clinical Immunology, Izmir, Turkey

**Correspondence**: Emine Nihal Mete Gökmen

*Clinical and Translational Allergy* 2016, **6(Suppl 3)**:P74

**Background**: Acute generalized exanthematous pustulosis (AGEP) is a rare eruption characterized by acute, extensive formation of sterile nonfollicular pustules on edematous- erythematous skin. It is accompanied by fever, peripheral blood leucocytosis neutrophilia, and sometimes by facial edema, hepatitis and eosinophilia. Most cases of AGEP (90 %) is caused by drugs and acute infections.

**Report**: 34 year- old female patient with ulserative colitis had been taken mesalazine tablets 2400 mg per day for 1 month. After 1 month therapy, a cutaneous reaction characterized by disseminated pustules on an erythematous base happened and mesalazine was stopped. During the period, lesions was localised to extremities, so topical therapy used. After the reaction was recovered, because of severe ulserative colitis, 15 days later mesalazine was restarted. Due to the second therapy, after the first dosage, she developed multiple disseminated sterile pustules on an erythematous background, associated with fever and neutrophilia. Laboratory examinations showed the elevation of white blood cells, neutrophils, erythrocyte sedimentation rate and C-reactive protein (white blood cells, 9110/mm^3^; (64.2 % neutrophils, 21.4 % lymphocytes, 2.7 % eosinophils, 11.4 % monocytes), erythrocyte sedimentation rate 13 mm/h, C-reactive protein level 3.27 mg/dl). Punch biopsy from pustules revealed subcorneal pustular formation, perivascular infiltration rich in leukocyte in dermis and acanthosis in epidermis. No accumulation was detected in immunofluorescence analyses. Clinical and histopathological findings were considered as AGEP. She was treated with intravenous corticosteroids, oral antihistamines and topical corticosteroids. The patient’s rashes decreased within the following 7 days and then diminished with desquamation.

**How this report contributes to current knowledge**: We report a patient with AGEP after the use of mesalazine. It’s important to consider AGEP in the differential diagnosis of acute pustular rashes and drugs should be investigated as causative agents. Knowledge of the clinical features are necessary to be distinguished from other entities. It’s the first AGEP case due to 5-ASA derived mesalazine in the literature. As mesalazine has been oftenly using for inflammatory bowel diseases, clinicians should be aware of its potential about developing AGEP and be careful about it. 

**Consent:** Written informed consent was obtained from the patient.

### P75 Changes of blood plasmacytoid dendritic cells, myeloid dendritic cells, and basophils during the acute stage of drug reaction with eosinophilia and systemic symptoms (DRESS) and other drug eruptions

#### Shao-Hsuan Hsu, Yung-Tsu Cho, Che-Wen Yang, Kai-Lung Chen, Chia-Yu Chu

##### Department of Dermatology, National Taiwan University Hospital and National Taiwan University College of Medicine, Taipei, Taiwan

**Correspondence**: Shao-Hsuan Hsu

*Clinical and Translational Allergy* 2016, **6(Suppl 3)**:P75

**Background**: Drug reaction with eosinophilia and systemic symptoms (DRESS) is a severe cutaneous drug reaction characterized by exanthematous skin rash, fever, lymphadenopathies, eosinophilia, atypical lymphocytosis and internal organ involvement. Reactivation of human herpes virus (HHV)-6 and decreased plasmacytoid dendritic cell (pDC) in the peripheral blood were putatively related to the disease, though the causality remained inconclusive.

**Materials and methods**: We recruited 18 patients from July 2013 to May 2014 with the diagnosis of drug eruptions including maculopapular eruptions (MPE), Stevens–Johnson syndrome (SJS), fixed drug eruption (FDE) and DRESS. The peripheral blood pDC (lin^−^CD123^+^CD11c^−^), myeloid DC (mDC, lin^−^CD123^−^CD11c^+^) and basophils (lin^−^HLA-DR^−^CD123^+^CD11c^+^) were simultaneously labeled and processed by 4-color assay method, with the frequencies of such cells counted by flow cytometry. HHV-6 reactivation was determined by the presence of viral DNA in the whole blood sample or the elevation of anti-HHV-6 IgG in the serum during the convalescent stage.

**Results**: Decreased frequencies of pDC were significantly associated with the diagnosis of DRESS (p = 0.004), presence of atypical lymphocytosis (p = 0.023) and HHV-6 reactivation (p = 0.043), while those of mDC and basophils were unrelated to the type of drug eruption, presence of atypical lymphocytosis, eosinophilia or HHV-6 reactivation. The reactivation of HHV-6 was only found in DRESS patients, in which the pDC frequencies consistently showed a decreasing trend.

**Conclusions**: In this study, we found that decreased pDC in the peripheral blood during the acute stage is more common in DRESS patients than the other drug eruptions, while only part of the patients demonstrated measurable HHV-6 reactivation. mDC and basophils did not show remarkable trend among different drug eruptions. The decrement of peripheral blood pDC might be the preceding, or might be the causative, event before HHV-6 reactivation.

**Keywords**: Drug reaction with eosinophilia and systemic symptoms; Plasmacytoid dendritic cell; Myeloid dendritic cell; Basophil; Human herpes simplex virus-6

### P76 Characterization of isoniazid/rifampicin-specific t-cell responses in patients with DRESS syndrome

#### Young-Min Ye^1^, Gyu-Young Hur^2^, Hae-Sim Park^1^, Seung-Hyun Kim^1^

##### ^1^Department of Allergy & Clinical Immunology, Ajou University School of Medicine, Suwon, South Korea; ^2^Department of Internal medicine, College of Medicine, Korea University, Seoul, South Korea

**Correspondence**: Seung-Hyun Kim

*Clinical and Translational Allergy* 2016, **6(Suppl 3)**:P76

**Background**: Antituberculosis drugs (ATDs) including isoniazid, rifampicin, pyrazinamide and ethambutol are commonly used for the treatment of tuberculosis, but occasionally associated with drug- induced hypersensitive reactions such as *drug* rash with *eosinophilia* and systemic symptoms (DRESS) syndrome and hepatitis. The culprit drug and mechanistic basis of the hypersensitive reaction has not been defined. The aim of this study was to find whether drug-responsive T cell response was detectable in patients with ATD-related DRESS and characterize the mechanistic features of the T-cell response.

**Materials and methods**: A lymphocyte transformation test (LTT) was performed using peripheral blood mononuclear cells (PBMCs) from ATD-induced DRESS patients. Subsequently, drug-specific T-cell clones were generated from the hypersensitive patients. We measured the drug-specific proliferative responses and interferon-gamma (IFN-γ) secretion. Anti-human class I and class II blocking antibodies were used to analyze HLA-restricted T-cell response.

**Results**: Strong proliferative responses to isoniazid or rifampicin were detectable in the patient with DRESS by LTT. Isoniazid/rifampicin- specific T-cell clones were generated from blood of the patients, but not pyrazinamide or ethambutol. The T cell clones proliferated and secreted IFNγ when stimulated with isoniazid or rifampicin. They did not cross-react with each other. T-cell responses were blocked in the presence of anti-human class II antibodies.

**Conclusions**: This study identifies isoniazid/rifampicin- responsive T-cells in peripheral blood of certain patients with ATD-induced DRESS. It highlights an important role of drug-responsive T-cell immune responses in ATD-induced DRESS syndrome.

**Keywords**: Antituberculosis drugs; Drug rash with eosinophilia and systemic symptoms; T cell response; HLA

### P77 DRESS syndrome secondary to sulfasalazine with delayed TEN: a case presentation

#### Syed Ali^1^, Michaela Lucas^2^, Peter N. Hollingsworth^3^, Andrew P. C. Mclean-Tooke^3^

##### ^1^Department of Immunology, Perth, Australia; ^2^Department of Immunology, Pathwest; QE2 Medical Centre; SMP, PALM, UWA; IIID, Murdoch University, Perth, Australia; ^3^Department of Immunology, Pathwest; QE2 Medical Centre, Perth, Australia

**Correspondence**: Syed Ali

*Clinical and Translational Allergy* 2016, **6(Suppl 3)**:P77

**Background**: Drug reaction with eosinophilia and systemic symptoms (DRESS) is a rare and potentially life threatening condition. DRESS preceding toxic epidermal necrolysis (TEN) has only been reported with three cases to date.

**Report**: A 31 year old lady presented with a 3 day history of fever, morbilliform rash, cervical lymphadenopathy and facial oedema. 5 weeks prior she had been diagnosed with rheumatoid arthritis (RA) and commenced on hydroxychloroquine and sulfasalazine. Investigations showed an eosinophilia, elevated liver function tests (LFTs) and high inflammatory markers. Infectious screen including viral tests was negative. A clinical diagnosis of DRESS was made, all RA drugs were ceased and she was started on high dose oral corticosteroids. During admission she developed a marked lymphocytosis, neutrophilia and eosinophilia with worsening LFTs and coagulopathy. The rash, LFTs and leukocytosis gradually improved and she was discharged on day 9 to continue on high dose oral steroids.

She presented 3 days later with worsening of her rash, high fevers and right upper quadrant abdominal pain associated with worsening of LFTs, anaemia and thrombocytopenia. 3 doses of IV methylprednisolone were administered. Haemophagocytic lymphohistiocytosis was considered given a high ferritin and hypofibrinogenaemia although bone marrow examination showed reactive changes but no evidence of haemophagocytosis. There was again improvement in rash, facial swelling and laboratory markers, and she was discharged to continue high dose steroids.

She re-presented 7 days later with return of fevers, rash, jaundice and deterioration in her LFTs. Cyclosporin was added but ceased after 4 days due to concerns regarding worsening LFTs. Liver biopsy showed submassive central necrosis and prominent bile duct damage. Ganciclovir was added following an equivocal PCR for HHV-6 on the liver biopsy tissue and she was considered for liver transplant if her liver failure worsened. On day 40 her LFTs had improved but she developed mucosal ulceration involving the mouth, eyes and genitals with bullous skin lesions involving 80 % of the body surface which was Nikolsky sign positive. Skin biopsy was consistent with TEN. She was transferred to the ICU for aggressive management and intravenous immunoglobulin but deteriorated and passed away the next day.

**How this report contributes to current knowledge**: Here, we present a fatal case of DRESS with fulminant liver failure followed by extensive TEN despite immunosuppression and broad anti-microbial cover.

**Consent**: Written informed consent was obtained from the patient for publication of this abstract and any accompanying images.

### P78 Drug rash with eosinophilia and systemic symptoms (DRESS) features according to the culprit drug

#### Zohra Chadly, Nadia Ben Fredj, Karim Aouam, Haifa Ben Romdhane, Naceur A. Boughattas, Amel Chaabane

##### Faculty of Medicine/University Hospital/University of Monastir, Monastir, Tunisia

**Correspondence**: Karim Aouam

*Clinical and Translational Allergy* 2016, **6(Suppl 3)**:P78

**Background**: The aim of this study was to evaluate the clinical and chronological Drug Rash with Eosinophilia and Systemic Symptoms (DRESS) characteristics according to the culprit drug.

**Materials and methods**: We carried out a retrospective study including all cases of DRESS notified to the Pharmacovigilance Unit of Monastir during 11 years. Diagnosis of DRESS was based on European *RegiSCAR* criteria. Imputability was established according to *Begaud’s* method. Skin tests were performed according *ENDA* recommendations.

**Results**: Fourty seven cases of DRESS were included in our study: 27 men and 20 women, with a mean age of 47 years ± 19. Antiepileptics drugs (18 cases: 15 carbamazepine, 2 phenobarbital, and 1 lamorigine) were the most frequent responsible drugs in our study followed by antibiotics (12: 6 betalactams, 4 glycopeptides, 1 cotrimoxazole and 1 ethambutol), allopurinol (10) and salazopyrin (7). All patients had pruritic maculopapular rash involving more than 50 % of their body surface area. Mucosal involvement was observed mainly with antiepileptics drugs (five cases) and allopurinol (four cases). Lymphadenopathy was more frequent with salazopyrin and antiepileptics drugs (55 % each). Eosinophilia was observed in 90 % of cases with allopurinol, 66 % with antibiotics and antiepileptics drugs, and 57 % with salazopyrin. Atypical lymphocytosis was observed only in eight cases. Liver was the most common organ affected (74.5 %) in our series. The most frequent type of liver injury was: cytolytic with antibiotics and allopurinol (58 and 30 % respectively) and mixed with salazopyrin and allopurinol (57 and 50 % respectively). Renal failure was observed in all cases induced by allopurinol. Pulmonary involvement was observed in five cases (three with salazopyrin and two with antiepileptic drugs). The mean incubation period was similar in the four groups of incriminated drugs. The outcome was favorable after drug withdrawal in 95.7 % of cases. Two patients with DRESS induced by allopurinol died because of multiple organ failure. Skin tests (patch or intradermal tests) were done in 33 cases. 75 % of skin tests with antiepileptics drugs and antibiotics were positive. Skin tests with salazopyrin and allopurinol were all negative.

**Conclusions**: Throughout this study, we point out the variability of DRESS clinical characteristics according to the culprit drug in one hand and the usefulness of skin tests with salazopyrin and allopurinol in the other hand.

### P79 Drug reaction with eosinophilia and systemic symptoms induced by allopurinol: not always easy to diagnose

#### Marina Lluncor Salazar^1^, Beatriz Pola^1^, Ana Fiandor^2^, Teresa Bellón^3^, Elena Ramírez^4^, Javier Domínguez Ortega^2^, Santiago Quirce^5^, Rosario Cabañas^2^

##### ^1^Allergy Department, La Paz Hospital Institute for Health Research (IdiPAZ), Madrid, Spain; ^2^Allergy Department, La Paz Hospital Institute for Health Research (IdiPAZ), Consorcio Piel en RED, Madrid, Spain; ^3^Immunology Department, La Paz Hospital Institute for Health Research (IdiPAZ), Consorcio Piel en RED, Madrid, Spain; ^4^Department of Clinical Pharmacology, Hospital La Paz Health Research Institute (IdiPAZ), School of Medicine, Universidad Autónoma de Madrid, Madrid, Spain. Consorcio Piel en RED, Madrid, Spain; ^5^Allergy Department, La Paz Hospital Institute for Health Research (IdiPAZ), Madrid, Spain

**Correspondence**: Marina Lluncor Salazar

*Clinical and Translational Allergy* 2016, **6(Suppl 3)**:P79

**Background**: DRESS is a life threatening hypersensitivity reaction. Allopurinol is a frequent cause. Lymphocyte transformation test (LTT) and epicutaneous tests with allopurinol are usually negative. We report a case with positive LTT to allopurinol and its metabolite, oxypurinol.

**Materials and methods**: A 37-year-old female attended the emergency room complaining of asthenia, cough, and generalized edema. She had a history chronic renal insufficiency stage 4 secondary to focal segmental glomerulosclerosis, high blood pressure and migraine. She was admitted with respiratory infection, acute kidney injury, and desquamative skin lesions that had appeared 20 days before. Initially she had erythema and face swelling, difficulty swallowing, painful papular lesions on face, scalp and upper trunk, abdomen and thighs. She had been treated with antihistamines, topical and systemic low dose corticosteroids with clinical improvement. She was on treatment with omeprazole, valsartan, prednisone, acetazolamide, allopurinol and ezetimibe/simvastatin. DRESS was suspected. A team of specialists (dermatologist, allergologist, pharmacologist, nephrologist) evaluated the patient. Laboratory tests and herpes virus serology were performed. After discharge she was evaluated on Allergy Unit. LTT was performed 2 months later according to Pichler et al. LTT is positive if stimulation index (SI) ≥2

**Results**: Tests revealed WBC 11,400, 700 eosinophils/mm^3^ (6.2 %), creatinine 4.38 mg/dl (baseline 2.8 mg/dl), CRP 498, GPT 483, GGT 1264 and hyperamilasemia. Serology was only positive for herpes 6(1/320). According to Kardaun score, “probable” DRESS diagnosis was established (four points). She was included in Piel en Red Registry. Causality assessment according the Spanish Pharmacovigilance System Algorithm (SFEV) results were: allopurinol +7(probable) and Conditional (Unrelated) the other drugs. Allopurinol treatment was stopped. She received a bolus of metylprednisolone intravenously three consecutive days, antihistamines and antibiotics with improvement. She was discharged 15 days later with corticosteroid treatment (reducing dose) during 14 weeks. Patch tests were negative with allopurinol, ezetimibe and simvastatine. LTT was positive to allopurinol and to its metabolite oxypurinol at different concentrations with the highest stimulation index for oxypurinol (SI 14.84)

**Conclusions**: We report a case of DRESS by allopurinol in which the diagnosis was difficult to establish. LTT has been useful to identify the etiological agent.

**Keywords**: DRESS; Allopurinol; Oxypurinol; LTT

**Consent**: Written informed consent was obtained from the patient for publication of this abstract and any accompanying images.

### P80 Drug reaction with eosinophilia and systemic symptoms syndrome induced by two drugs simultaneously: a case report

#### Krasimira Baynova, Marina Labella, Manuel Prados

##### HUVR Seville, Seville, Spain

**Correspondence**: Krasimira Baynova

*Clinical and Translational Allergy* 2016, **6(Suppl 3)**:P80

**Background**: Drug reaction with eosinophylia and systemic symptoms (DRESS) is a severe potentially life-threatening drug hypersensitivity reaction with delayed onset (2–8 weeks after beginning a drug intake). It is characterized by rash, fever, blood abnormalities and/or internal organ involvement. Most frequently implicated drugs in DRESS are anti-epileptic drugs. We present a case of DRESS induced by carbamzepine and paracetamol simultaneously.

**Report**: A 84 year-old Caucasian woman with no previous drug hypersensitivity history, complained of generalized maculopapular itchy purplish rash (trunk, abdomen, upper and lower extremities), peeling skin, fever and general weakness. A month before she was diagnosed of trigeminal neuralgia and started a treatment with pregabalin, carbamazepine and paracetamol. Previously the patient had used paracetamol without adverse reactions. Pregabalin and carbamazepine were used for the first time. Symptoms disappeared when the three drugs were interrupted and oral corticoid treatment was established.

Peripheral blood test was done when patient was symptomatic. Skin patch test and lymphocyte activation test (LAT)with paracetamol, carbamazepine and pregabalin were performed 4 weeks after complete recovering. In blood test was observed eosinophylia (1000/mm^3^) and leucocytosis (>11,000/mm^3^). Kidney and liver function were normal. Skin patch test (interpretation in 48 and 96 h) and lymphocyte activation test were positive to paracetamol and carbamazepine, and negative to pregabalin.

Pregabalin treatment was continued without adverse reactions. A few months later our patient took a pill of paracetamol and experienced eosinophylia and maculopapular rush in abdomen and lower extremities.

**How this report contributes to current knowledge**: DRESS is a type IV drug hypersensitivity reaction and is commonly induced by aromatic anticonvulsants as carbamazepine. Nevertheless any drug intake could induce this severe reaction, including “innocuous” drugs as paracetamol. The allergy work-out should be carried out 4–6 weeks after the complete recovering and should include all involved drugs.

**Consent**: Written informed consent was obtained from the patient for publication of this abstract and any accompanying images.

### P81 The drug reaction with eosinophilia and systemic symptoms (DRESS) induced by the second-line antituberculosis drugs and Epstein–Barr virus infection

#### Agne Ramonaite, Ieva Bajoriuniene, Brigita Sitkauskiene, Raimundas Sakalauskas

##### Department of Pulmonology and Immunology, Lithuanian University of Health Sciences, Kaunas, Lithuania

**Correspondence**: Agne Ramonaite

*Clinical and Translational Allergy* 2016, **6(Suppl 3)**:P81

**Background**: Drug reaction with eosinophilia and systemic symptoms (DRESS) is a severe drug-induced reaction that involves both skin and viscera. Antiepileptic agents, allopurinol and sulfonamides are the most frequently reported causes. Other causal factors such as drug metabolites, genetic factors and viral infections have been also reported.

**Report**: 31-year-old female was admitted to the hospital for the treatment of multidrug-resistant pulmonary tuberculosis. She was treated with second-line antituberculosis drugs: moxifloxacin, kanamycin, cycloserine, prothionamide, para-aminosalicylic acid. After 3 weeks of therapy she developed high fever (>39 °C), lymphadenopathy in the cervical and axillary regions and pruritic maculopapular eruption all over the body. Hematologic abnormalities such as leukocytosis with eosinophilia (1.81 × 10^9^/l) and monocytosis (1.85 × 10^9^/l) were detected in peripheral blood of the patient. Hepatitis was asymptomatic and detected using the liver function tests: serum aspartate aminotransferase (AST 1379 IU/l) and alanine aminotransferase (ALT 1221 IU/l) levels were increased by approximately 30–40-fold above the normal limits. The positive diagnosis of Epstein–Barr infection was based on an increase in the anti Epstein–Barr immunoglobulin G titer, implicating an Epstein–Barr virus reactivation. Based on the clinic and laboratory findings diagnosis of DRESS was suspected, and all the drugs were discontinued. Patient’s condition improved after 8 weeks. The skin patch tests with moxifloxacin, kanamycin, cycloserine, prothionamide and para-aminosalicylic acid were done 2 months after the hypersensitivity syndrome resolved. The patch tests showed a positive reaction to prothionamide and para-aminosalicylic acid.

**How this report contributes to current knowledge**: This case reports the development of DRESS caused by late type of hypersensitivity to second-line antituberculosis drugs (prothionamide and para-aminosalicylic acid) in association with Epstein–Barr virus infection.

**Consent**: Written informed consent was obtained from the patient for publication of this abstract and any accompanying images.

## Poster Walk 10: Miscellaneous drug hypersensitivity (P82–P91)

### P82 A case of cycloserine-induced lichenoid drug eruption confirmed with a lymphocatye transformation test

#### Jae-Woo Kwon^1^, Shinyoung Park^2^

##### ^1^Department of Allergy and Clinical Immunology, Kangwon National University School of Medicine, Chuncheon, Korea; ^2^The Research Department, Kangwon Regional Cancer Center, Kangwon National University Hospital, Chuncheon, Korea

**Correspondence**: Jae-Woo Kwon

*Clinical and Translational Allergy* 2016, **6(Suppl 3)**:P82

**Background**: LDE (Lichenoid drug eruption) is a rare form of delayed type drug eruptions. Among anti-Tb (antituberculosis) drugs, ethambutol is one of the most common causative drugs to induce LDEs and cycloserine has been reported known as a rare causative drug of the LDEs.

**Report**: 38 years old man presented with pururitus, lichenoid skin lesion on whole body, and blood eosinophilia (16,824/µl). He had been treated with isoniazid, rifampicin, ethambutol, and pyrazinamide for 2 months and then with ethambutol, levofloxacin, cycloserine for next 2 months because of elevated liver enzymes. Mild pruritus developed at the start of anti-Tb medications and aggravated with development of lichenoid skin lesion 1 month ago. Pruritus, skin lesions, and eosinophilia were improved since anti-Tb medications were stopped. Patch test showed mild reaction for ethambutol and strong reaction for cycloserine. Then we performed an LTT (lymphocyte transformation test) and successfully confirmed cycloserine as the offending drug.

**How this report contributes to current knowledge**: This suggests that the cycloserine should be considered as a possible causative drug of LDE and an LTT could be an option for the diagnosis of lichenoid drug eruption due to cycloserine.

**Consent**: Written informed consent was obtained from the patient for publication of this abstract and any accompanying images.

### P83 Allergic reaction to topical eye drops: 5 years’ retrospective study in a drug allergy unit

#### Diana Silva^1^, Leonor Carneiro Leão^1^, Fabricia Carolino^2^, Eunice Castro^2^, Josefina Cernadas^2^

##### ^1^Allergy and Clinical Immunology Department, São João Medical Center; Laboratory of Basic & Clinical Immunology, Faculty of Medicine, Porto University, Porto, Portugal; ^2^Allergy and Clinical Immunology Department, São João Medical Center, Porto, Portugal

**Correspondence**: Diana Silva

*Clinical and Translational Allergy* 2016, **6(Suppl 3)**:P83

**Background**: Ophthalmic products are widely used and long-term application is frequently needed. This might lead to ocular surface changes and increase the frequency of adverse reactions. However, the underlying mechanisms and the causal agent are usually difficult to ascertain. We aimed to evaluate the patients referred to our drug allergy unit with suspected hypersensitivity reaction to topical eye drops.

**Materials and methods**: A cross-sectional, retrospective analysis of the clinical files of all patients studied in the Drug Allergy Unit of a University Hospital, in the last 5 years (January 2010 to December 2015) was made. Those with a suspected hypersensitivity reaction to topical eye drops were selected. Demographic, clinical history and diagnostic procedures data were collected.

**Results**: Four patients, two children (2 and 11 years of age) and two adults (49 and 72 years), were referred due to a suspicion of hypersensitivity reaction to topical eye drops. Both children reacted after atropine conjunctival application. The two adults reacted with each of the following topical drugs: timolol and moxifloxacin with tobramycin. All showed local symptoms of conjunctival erythema and ocular/facial edema immediately after drug administration. The patient who reacted to timolol presented dyspnea. For diagnostic study, the 2-year-old child performed conjunctival provocation test (CPT) with increasing doses of atropine (1; 5 and 10 mg/ml), with positive reaction with facial erythema at 10 mg/ml dose; the 11-year-old child was submitted to skin prick and intradermal tests with atropine, which were negative, and waits for CPT to complete the study. The two adult patients performed only diagnostic CPT. The 49-year-old woman, which reacted with timolol, did CPT with spirometry control, without a significant change in FEV1 or any clinical symptoms; the 72-year-old male performed two separate CPT with moxifloxacin and tobramycin, both with negative results.

**Conclusions**: Hypersensitivity reactions to topical eye drops are probably underreported in our clinical practice. In three out of four patient’s hypersensitivity reaction was excluded after diagnostic work-up. Facial erythema is a frequent dose-dependent adverse effect to topical atropine, graded conjunctival challenge is important for establishing the diagnosis. Awareness should be increased for unneeded avoidance of topical eye drops.

**Keywords**: Drug allergy; Ocular allergy

### P84 Allergy to heparins

#### Diana Perez-Alzate, Natalia Blanca-López, Maria Luisa Somoza Alvarez, Maria Garcimartin, Maria Vazquez De La Torre, Francisco Javier Ruano Pérez, Elisa Haroun, Gabriela Canto Diez

##### Hospital Universitario Infanta Leonor, Madrid, Spain

**Correspondence**: Diana Perez-Alzate

*Clinical and Translational Allergy* 2016, **6(Suppl 3)**:P84

The published version of this abstract can be found at [1].

**Reference**

1. Javier Ruano Perez F, Perez Alzate D, Blanca-Lopez N, Somoza ML, Vazquez de la Torre M, Garcimartin MI, Haroun E, Canto G. Allergy to heparins. J Allergy Clin Immunol. 2016;137(2, Supplement):AB46.

### P85 Allopurinol-induced adverse drug reactions

#### Katinka Ónodi-Nagy^1^, Ágnes Kinyó^2^, Lajos Kemény^1^, Zsuzsanna Bata-Csörgo^1^

##### ^1^Department of Dermatology and Allergology, Szeged, Hungary; ^2^Department of Dermatology, Venereology and Oncodermatology, Pécs, Hungary

**Correspondence**: Katinka Ónodi-Nagy

*Clinical and Translational Allergy* 2016, **6(Suppl 3)**:P85

**Background**: Allopurinol, a xanthine oxidase inhibitor, is the most widely administered urate lowering drug in the long-term management of chronic hyperuricemia and gout. In 2 % of the treated patients adverse drug reactions, life-threatening cutaneous and systemic symptoms can develop. Human leukocyte antigen (HLA) genes play central role in immune reactions. Important association between HLA-B*5801 allele and allopurinol-induced severe cutaneous adverse drug reactions have been reported.

**Materials and methods**: In the past few years an increase in allopurinol-induced adverse drug reaction have been experienced among patients at our clinic (University of Szeged). Therefore we decided to investigate the clinical characteristics of these patients.

**Results**: Between 2002 and 2008, 81 patients were sent to our laboratory center with suspected allopurinol hypersensitivity for Lymphocyte Transformation Test (LTT). This number rose to 222 between 2009 and 2015. LTT was positive in four cases out of the 81 patients and in 20 cases out of the 222 patients respectively, indicating 4.9 and 9 % sensitivity. Of all these patients we were able to obtain a complete clinical history of 35 patients (mean age 68 years), 4 patients in the first period and 31 patients in the second period. They presented generalized maculopapular exanthems in 37.14 %, drug reaction with eosinophilia and systemic symptoms in 31.43 %, Stevens–Johnson syndrome in 8.57 %, erythema multiforme in 5.71 %, vasculitis in 5.71 %, bullous drug exanthems in 2.86 %, toxic epidermal necrolysis in 2.86 %, acute generalized exanthematous pustulosis in 2.86 % and erythroderma in 2.86 %. The primary indication of the treatment was asymptomatic hyperuricemia in 88.6 % of the patients. We concluded that the concomitant use of allopurinol and certain diuretics, furosemide and/or hydrochlorothiazide in 28 cases of our 35 patients, and impaired renal function enhance allopurinol toxicity increasing the risk of adverse drug reaction developments. Evaluation of the HLA-B*5801 studies are in progress.

**Conclusions**: Our data show that the use of allopurinol and thus the number of the resulting hypersensitivity reactions is increasing. The more and more common hypersensitivity reactions may be the result of the improper drug prescription, indication and advanced age. Based on our results LTT is not sensitive enough in proving allopurinol-induced adverse drug reactions.

**Keywords**: Allopurinol; Adverse reactions; Clinical characteristics

### P86 Analysis of a population with immediate hypersensitivity to corticosteroids: an 11 year review

#### Joana Sofia Pita^1^, Emília Faria^1^, Rosa Anita Fernandes^1^, Ana Moura^1^, Nuno Sousa^2^, Carmelita Ribeiro^1^, Carlos Loureiro^1^, Ana Todo Bom^1^

##### ^1^Centro Hospitalar e Universitário de Coimbra, Coimbra, Portugal; ^2^Centro Hospitalar de Leiria, Leiria, Portugal

**Correspondence**: Joana Sofia Pita

*Clinical and Translational Allergy* 2016, **6(Suppl 3)**:P86

**Background**: Due to their anti-inflammatory and immunomodulatory properties, corticosteroids are highly prescribed. The prevalence of hypersensitivity (HS) reactions is estimated at <1 %. The aim of our study was to characterize a population of patients referred to our outpatient clinic for suspected immediate HS to corticosteroids.

**Materials and methods**: Retrospective analysis including 61 patients sent to our Drug Allergy consultation for suspected corticosteroid HS, from January 2005 to December 2015. We proceeded to the patients’ clinical evaluation and analysis of the results of skin prick (SPT) and intradermal tests (IDT) of the following drugs: prednisolone succinate 125 mg/ml, dexamethasone sodium phosphate 5 mg/ml, methylprednisolone sodium succinate 40 mg/ml, hydrocortisone 100 mg/ml, betamethasone dipropionate 5 mg/ml. IDT were performed with the following dilutions: 1:1000, 1:100 and 1:10. The tests were read at 20 min, and at 24–48 h.

**Results**: We analysed 61 patients with suspected immediate HS to corticosteroids, in which 77 % was female, with a median age of 47 years. Twelve patients had positive tests results (19 %). The population with positive results was mainly female, 58 %, with a median age of 39 (± 16 years). Half of this population had associated atopic disease (asthma and/or rhinitis). The clinical manifestations after the drug administration were: anaphylactic reactions in 50 %, urticaria and/or angioedema in 41 % and syncope in 8 %. In these 12 patients we obtained 6 % of positive SPT, and 12 % of positive IDT. Thirty percent of positive reactions occurred with methylprednisolone, followed by dexamethasone (26 %), prednisolone (17 %), hydrocortisone (13 %) and betamethasone (13 %). Eight patients (66 %) presented HS to more than one corticosteroid.

**Conclusions**: We obtained 19 % positive prick and intradermal tests to corticosteroids, being anaphylaxis the most common reaction in these patients. There was a high frequency of sensitization to methylprednisolone and dexamethasone. 66 % of patients had HS to more than one corticosteroid, which probably correlates with the presence of cross-reactivity among these drugs.

**Keywords**: Hypersensitivity; Corticosteroids; Skin tests

### P87 Anaphylaxis against mivacurium in a 12-months old boy at first-time exposure

#### Wolfgang Pfützner

##### Department of Dermatology and Allergology, Philipps University Marburg, Marburg, Germany

**Correspondence**: Wolfgang Pfützner

*Clinical and Translational Allergy* 2016, **6(Suppl 3)**:P87

**Background**: Since sensitization against an allergen is an important requirement for the development of anaphylaxis, drug hypersensitivity reactions are very uncommon in infants. We report on a 12-months old boy who experienced a severe anaphylactic reaction during perioperative anaesthesia.

**Materials and methods**: About 5 min after receiving fentanyl, propofol and mivacurium for the induction of anaesthesia, a persistent rush was noticed, followed by a fall of blood pressure down to 70/40 mm Hg, tachycardia (f = 150/min) and respiratory distress. Emergency treatment was initiated by i.v.-application of epinephrine, after which the little boy fully recovered. Two months later he was referred to our department for allergological evaluation, including both laboratory analysis and skin tests with different drugs.

**Results**: Total IgE was 25.5 kU/l with no allergen-specific IgE-antibodies detectable against pholcodine, latex, egg white and soja, and basal serum mast cell tryptase was 2.77 µg/l (Thermo Fisher, Germany). Skin prick tests revealed a positive result to mivacurium but showed negative reactions against fentanyl, remifentanyl, propofol, rocuronium, and cis-atracurium. Thus, diagnosis of drug allergy against mivacurium was established, together with cross-reactive sensitization against another neuromuscular blocking agent (NMBA), cis-atracurium. Surgery was rescheduled utilizing rocuronium for muscle relaxation, which was well tolerated.

**Conclusions**: NMBAs are the major cause of perioperative anaphylaxis, accounting for about 70 % of all cases, with IgE-antibodies directed against quaternary ammonium compounds (QAC). Hypersensitivity reactions against NMBAs without prior exposure to this drug class have been reported previously, suggesting sensitization induced by other QAM-containing substances like disinfectants, food or industrial materials. However, we are not aware about reports of anaphylaxis against NMBAs in infants of such a young age. Thus, this case underlines both the risk of allergic reactions against NMBAs at first exposure and the necessity of comprehensively testing these drugs even in very small children with the history of perioperative anaphylaxis.

**Keywords**: Anaphylaxis; Mivacurium; Infant; Anaesthesia

**Consent**: Written informed consent was obtained from the patient for publication of this abstract and any accompanying images.

### P88 Antihistamine-exacerbated chronic spontaneous urticaria: a paradox?

#### Nadine Marrouche, Clive Grattan

##### Norfolk and Norwich University Hospital, Norwich, United Kingdom

**Correspondence**: Nadine Marrouche

*Clinical and Translational Allergy* 2016, **6(Suppl 3)**:P88

**Background**: Histamine released from mast cells plays a key role in the pathogenesis of chronic spontaneous urticaria (CSU). However, it is unlikely that histamine alone is the only mediator of the disease. From clinical experience we know that H1-antihistamines, even at high doses, are ineffective in at least 30 % of patients. Antihistamine hypersensitivity has been reported in the literature but CSU exacerbation by multiple antihistamines in the same patient is rare.

**Report**: A 38-year old female patient presented with a 1-year history of recurrent itchy hives. The clinical history was suggestive of CSU. She was prescribed various antihistamines including chlorphenamine, loratadine, fexofenadine, and cetirizine. The patient noticed significant worsening of her urticaria within an hour of taking any antihistamine. Her urticaria exacerbations responded well to systemic steroids. The patient was re-challenged with cetirizine when her CSU was in remission. Within 90 min, she developed generalized itchy wheals which lasted several days. A skin biopsy showed typical urticaria with unusual prominence of eosinophils suggestive of urticaria with superimposed drug reaction (Figure 1). The patient was subsequently challenged with oral acrivastine and a similar reaction was observed.

**How this report contributes to current knowledge**: The underlying mechanism of antihistamine hypersensitivity remains unclear. Our patient had a positive oral challenge to multiple antihistamines with at least a 90-min delay which suggests that the underlying mechanism is likely non-immunological. In one case report it was suggested that, in some patients, antihistamines could paradoxically shift the H1 histamine receptor to the active confirmation leading to adverse reactions after dosing [*]. Our case highlights the rare possibility of a drug most commonly used to treat urticaria acting as the causal agent itself.

*Urticaria induced by antihistamines. González de Olano D, et al. J Investig Allergol Clin Immunol. (2006)

**Consent**: Written informed consent was obtained from the patient for publication of this abstract and any accompanying images.

### P89 Anti-osteoporotic agents-induced cutaneous adverse drug reactions in Asians

#### Yu-En Chen^1^, Chun-Bing Chen^2^, Wen-Hung Chung^2^, Yu-Ping Hsiao^3^, Chia-Yu Chu^4^

##### ^1^College of Medicine, Chung Shan Medical University Hospital and Chung Shan Medical University, Taichung, Taiwan; ^2^Department of Dermatology, Drug Hypersensitivity Clinical and Research Center, Chang Gung Memorial Hospitals, Linkou, Taipei, Keelung, Taiwan; ^3^Department of Dermatology, Chung Shan Medical University Hospital and Chung Shan Medical University College of Medicine, Taichung, Taiwan; ^4^Department of Dermatology, National Taiwan University Hospital and National Taiwan University College of Medicine, Taipei, Taiwan

**Correspondence**: Yu-En Chen

*Clinical and Translational Allergy* 2016, **6(Suppl 3)**:P89

**Background**: New medications such as bisphosphonates and strontium ranelate (a strontium salt of ranelic acid) have been introduced in the market for the treatment of osteoporosis and there are few case reports of severe cutaneous adverse reactions (SCAR) related to anti-osteoporotic agents. Therefore, we tried to identify the association between anti-osteoporotic agents and cutaneous adverse drug reaction (cADR) and the appropriate selection of alternative drugs.

**Fig. 3:**
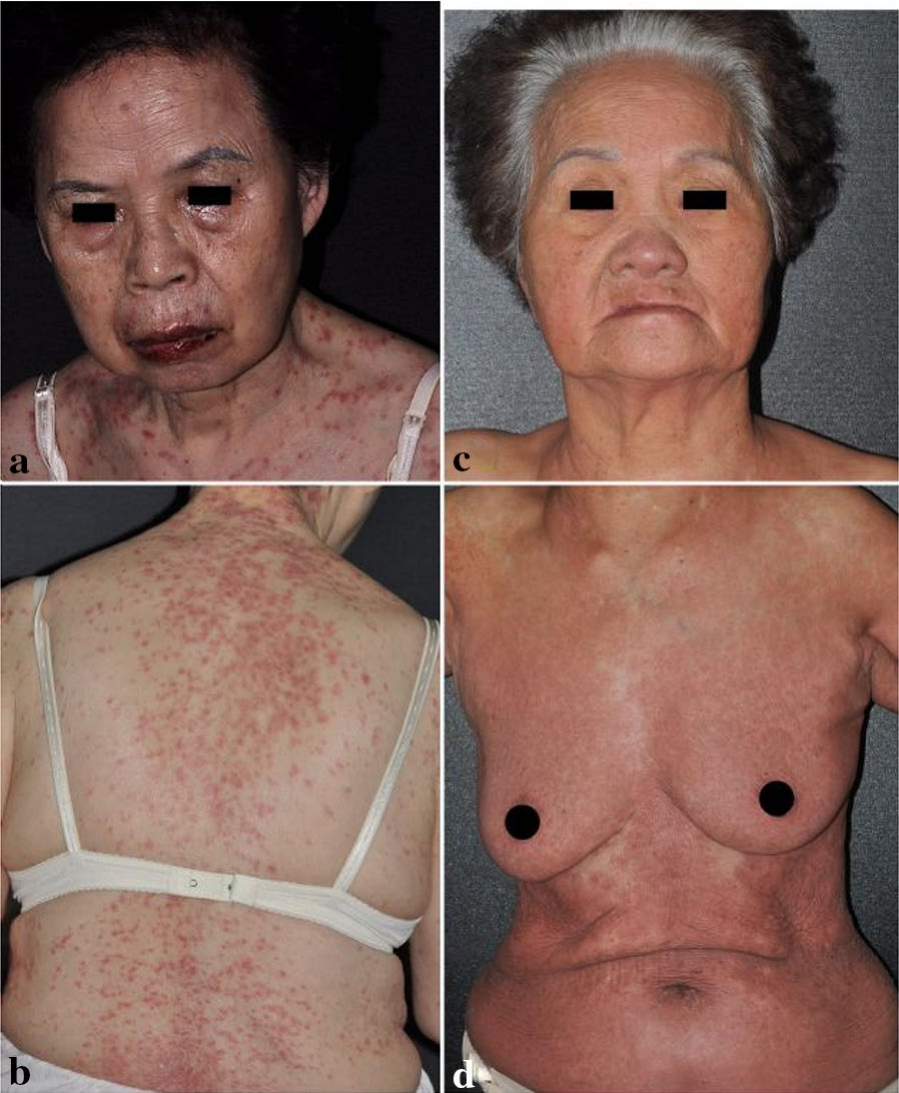
Clinical presentations of strontium ranelate-induced severe cutaneour adverse drug reactions. A case of Stevens–Johnson syndrome (SJS) had necrotic erosive lesions on the lips and scattered dusky red macules over the face and neck (**a**), and close up view of the nape and back with dusky macules/patches and some necrotic epidermal detachment (**b**). Another case of drug reaction with eosinophilia and systems symptom presented with facial edema (**c**), and extensive confluent infiltrative erythematous eruption disseminated to the truck (**d**)

**Materials and methods**: We retrospectively analyzed cADRs, including maculopapular exanthema (MPE),Stevens–Johnson syndrome (SJS), drug rash with eosinophilia and systemic symptoms (DRESS), related to use of anti-osteoporotic agents in Taiwan and Hong Kong from 2011 to 2015. We analyzed the causative anti-osteoporotic agents, clinical characteristics, outcomes and further assessed patients’ tolerability to alternative anti-osteoporotic agents after the development of anti-osteoporotic agents-related cADRs. We also review the literatures of anti-osteoporotic agents-related SCAR (Table 2; Fig. 3).

**Table 2:** Strontium ranelate–induced severe cutaneous adverse reactions

Region	Asia		Europe
Case series	This study	Tan et al. [1]/Lee et al. [2]	Cacoub et al. [3]
Country	Taiwan and Hong Kong	Singapore	France etc.
Culprit anti-osteoporotic agent	SR^1^	SR	SR
Indication to anti-osteoporotic agent	OPO	PM OPO^2^	OPO
Patients’ profile			
Ethnicity	Chinese	Chinese	Caucasian
Gender	F (83.3)	F (100.0)	F (100.0)
Average age (year)	64.7	69.5	68.7 (in DRESS group)
No. of SCARs patients	7	2	52
Phenotype			
SJS/TEN^3^			
Total case no.	6 (85.7)	2 (100.0)	5 (9.6)
Average latent period (days)	29.2	16	Not mentioned
Eye involvement^a^	1 (16.7)	1 (50)	Not mentioned
Orogenital involvement	5 (83.3)	2 (100.0)	Not mentioned
DRESS^4^			
Total case No.	1 (14.3)	0	47(90.4)
Average latent period (days)	90.0	0	33.5
Liver involvement^b^	0 (0)	0 (0)	37 (79.0)
Kidney involvement^c^	0 (0)	0 (0)	12 (25.0)
Eosinophilia^d^	1 (100.0)	0 (0)	43 (91.0)
Clinical sequelae	Nil	Nil	Persistent DRESS symptoms (21.3) and relapse of DRESS (2.0)
Mortality rate	0 %	0 %	8.5 % (in DRESS group)

Abbreviations: ^1^ *SR* Strontium ranelate, ^2^ *PM OPO* postmenopausal osteoporosis, ^3^ *SJS/TEN* Stevens–Johnson syndrome/toxic epidermal necrolysis, ^4^ *DRESS* drug rash with eosinophilia and systemic symptoms

*data are n(%) of patients unless otherwise specified

^a^Eye involvement: corneal ulcer or symblepharon, ^b^ Liver involvement: twofold increase from normal or baseline levels of serum glutamic oxaloacetic transaminase (GOT),glutamic pyruvate transaminase (GPT) or total bilirubin

^c^Kidney involvement: >1.5-fold elevation of serum creatinine from the normal range

^d^Eosinophilia: eosinophils count >500/μL

**References**

1. Tan KW, Wang YS, Tay YK. Stevens–Johnson syndrome due to strontium ranelate. Ann Acad Med Singap. 2011;40(11):510–1.

2. Lee HY, et al. Strontium ranelate-induced toxic epidermal necrolysis in a patient with post-menopausal osteoporosis. Osteoporos Int. 2009;20(1):161–2.

3. Cacoub P, et al. Drug rash with eosinophilia and systemic symptoms (DRESS) in patients receiving strontium ranelate. Osteoporos Int. 2013;24(5):1751–7.

**Results**: There were 14 cases of anti-osteoporotic agents-related cADRs, including 6 SJS, 1 DRESS and 7 MPE. The most common causative agents were strontium ranelate and bisphosphonates. Strontium ranelate was found to be related to most SCAR cases, including six SJS and one DRESS. For MPE, there were three cases caused by bisphosphonates, three cases caused by strontium ranelate and one case caused by teriparatide. There was no mortality and long-term sequelae of all SCAR cases. There was no cross hypersensitivity among strontium ranelate, bisphosphonates, and denosumab (a monoclonal antibody). Patients with strontium ranelate-related cADRs were tolerant of alternative bisphosphonates and denosumab. One case of bisphosphonate-induced MPE was also tolerant of denosumab.

**Conclusions**: Most of the anti-osteoporotic agents-related cADRs are usually mild. In Asia, strontium ranelate seems to cause more SJS/TEN than DRESS in comparison with that in Europe. No mortality was reported among patient with SCARs. Owing to the structural difference in anti-osteoporotic agents, denosumab was well tolerated in patients allergic to either strontium ranelate or bisphosphonates as alternative drug.

**Keywords**: Severe cutaneous adverse reactions; Anti-osteoporotic agents; Bisphosphonates; Strontium ranelate; Denosumab

**Consent**: Written informed consent was obtained from the patient for publication of this abstract and any accompanying images.

### P90 Diagnosis of allergic reactions to eye drops

#### Maria Vazquez De La Torre, Natalia Blanca-Lopez, Diana Perez-Alzate, Maria Isabel Garcimartin, Francisco Javier Ruano^1^, Maria Luisa Somoza, Elisa Haroun, Gabriela Canto

##### Hospital Infanta Leonor, Madrid, Spain

**Correspondence**: Natalia Blanca-Lopez

*Clinical and Translational Allergy* 2016, **6(Suppl 3)**:P90

The published version of this abstract can be found at [1].

**Reference**

1. Poster discussion session PDS 1. Allergy 2015;70:113–279. doi:10.1111/all.12717.

### P91 Diagnostic approach in suspected hypersensitivity reactions to corticosteroids

#### Fabrícia Carolino, Eunice Dias De Castro, Josefina R. Cernadas

##### Serviço de Imunoalergologia, Centro Hospitalar São João E.P.E., Porto, Portugal

**Correspondence**: Fabrícia Carolino

*Clinical and Translational Allergy* 2016, **6(Suppl 3)**:P91

**Background**: Hypersensitivity reactions (HSR) to corticosteroids (CS) are rare although there are a growing number of reports both to systemic and topical CS. There are no standardized procedures for diagnostic skin testing with these drugs but a panel is recommended to assess cross-reactivity between steroids. Incremental drug challenge is still necessary for diagnostic or tolerance assessment purposes, but in this point safety issues may overcome.

**Materials and methods**: Retrospective analysis of consecutive patients evaluated in our Drug Allergy Unit for suspected CS HSR, during a 5-years-period. Skin prick tests (SPT) and intradermal tests (IDT) were performed with commercially available sterile CS formulations—betamethasone (7 mg/ml), budesonide (0.5 mg/ml), dexamethasone (4 mg/ml), hydrocortisone (100 mg/ml), methylprednisolone (62.5 mg/ml) and/or prednisolone (25 mg/ml). Oral solution of deflazacort (22.75 mg/ml) and nasal-spray suspension of fluticasone (27.5 μg/dose) were also used for SPT. Patch tests (PT) with a standard and/or complementary CS series were performed. Drug challenges (DC) used a selected-CS dose ranging between approximately 0.5 and 1.5 mg/kg/day.

**Results**: A total of 31 patients were assessed (74.2 % females, mean ± SDage 36.8 ± 23.2 years) for suspected CS HSR. The main implicated CS were oral deflazacort (n = 11) and oral betamethasone (n = 5). 45.2 % had immediate reactions and 38.2 % late-onset symptoms; 51.6 % presented skin/mucosal manifestations. IDT were performed in 12 patients and were positive to at least one of the tested drugs in four (two with anaphylaxis and two with late-onset skin/mucosal involvement); two patients tested positively in IDT for more than one CS (dexamethasone/hydrocortisone and methylprednisolone/hydrocortisone). IDT were also performed in non-atopic controls. Patch tests in 10 patients revealed positive results (including the suspected CS) in two of them. DC was undertaken with the suspected systemic CS in eight patients (no positive challenges and two doubtful); the remaining patients were tested for an alternative CS.

**Conclusions**: Proper validated skin tests may provide the necessary diagnostic evidence in drug allergy. They are particularly useful in more severe index reactions when re-challenging is not an option. The accuracy of skin tests needs to be further established with larger studies.

**Keywords**: Drug hypersensitivity; Corticosteroids; Skin tests

